# *In Silico* Identification and Experimental Validation of Distal Activity-Enhancing Mutations in Tryptophan Synthase

**DOI:** 10.1021/acscatal.1c03950

**Published:** 2021-10-28

**Authors:** Miguel A. Maria-Solano, Thomas Kinateder, Javier Iglesias-Fernández, Reinhard Sterner, Sílvia Osuna

**Affiliations:** † CompBioLab Group, Institut de Química Computacional i Catàlisi (IQCC) and Departament de Química, Universitat de Girona, Maria Aurèlia Capmany 69, Girona 17003, Spain; ‡ Institute of Biophysics and Physical Biochemistry, Regensburg Center for Biochemistry, University of Regensburg, Universitätsstrasse 31, Regensburg 93053, Germany; § ICREA, Pg. Lluís Companys 23, Barcelona 08010, Spain; ∥ Global AI Drug Discovery Center, College of Pharmacy and Graduate School of Pharmaceutical Science, Ewha Womans University, Seoul 03760, Republic of Korea; ⊥ Nostrum Biodiscovery, Carrer de Baldiri Reixac, 10-12, Barcelona 08028, Spain

**Keywords:** tryptophan synthase, distal mutations, allostery, enzyme design, shortest path map, ancestral sequence reconstruction

## Abstract

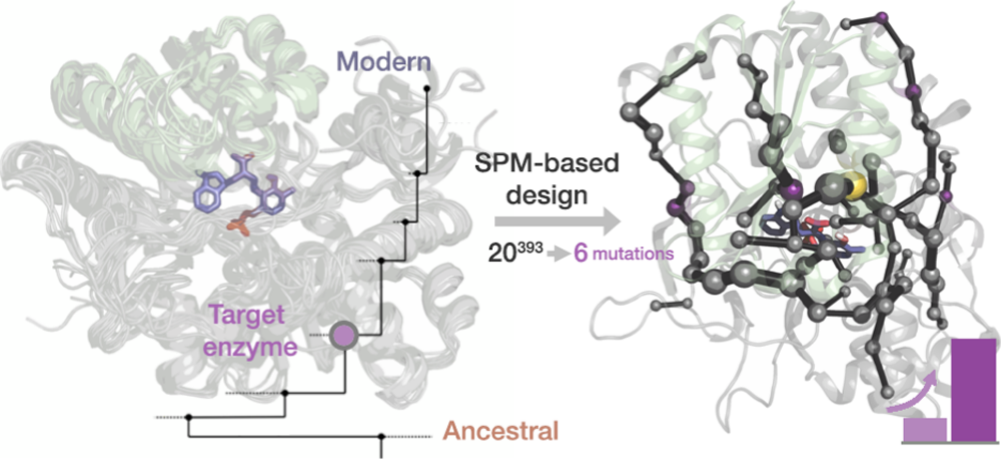

Allostery is a central mechanism for the regulation of multi-enzyme complexes. The mechanistic basis that drives allosteric regulation is poorly understood but harbors key information for enzyme engineering. In the present study, we focus on the tryptophan synthase complex that is composed of TrpA and TrpB subunits, which allosterically activate each other. Specifically, we develop a rational approach for identifying key amino acid residues of TrpB distal from the active site. Those residues are predicted to be crucial for shifting the inefficient conformational ensemble of the isolated TrpB to a productive ensemble through intra-subunit allosteric effects. The experimental validation of the conformationally driven TrpB design demonstrates its superior stand-alone activity in the absence of TrpA, comparable to those enhancements obtained after multiple rounds of experimental laboratory evolution. Our work evidences that the current challenge of distal active site prediction for enhanced function in computational enzyme design has become within reach.

## Introduction

Enzymes are some of the most sophisticated biomolecules that exist on Earth. They achieve impressive rate accelerations, thanks to their highly preorganized active site pockets, while exhibiting remarkable conformational flexibility key for their function, regulation, and evolution.^1−7^ Enzymes are dynamic biological entities, whose catalytic activities are directly related to their structure and the broad ensemble of conformations they sample in solution.^4−6^ This conformational equilibrium can be shifted, for example, by the binding of a ligand to a given site. This in turn influences the binding or the turnover of a substrate at the active site of the enzyme, a phenomenon that is called “allostery.”^8−10^ Likewise, the introduction of an amino acid substitution in the protein sequence not only induces an evident structural change but also a redistribution of the conformational ensemble, which in turn can potentially impact catalytic activity.^4,6,11,12^ Indeed, it has been proven that allosteric effects are not restricted to effector binding, but instead single point mutations or covalent attachment (e.g., phosphorylation), among others, can induce similar responses.^9,13,14^

Identifying mutations that modulate enzyme activity is the primary goal of enzyme engineering. One approach to enzyme engineering is directed evolution (DE), which has been applied to a myriad of enzyme systems successfully identifying active site and distal mutations, providing access to impressive tailor-made enzyme variants at the expense of large and expensive screening efforts.^15−18^ Rational design emerged as an attractive alternative to decrease the screening efforts to a reduced number of promising enzyme variants based on prior structural knowledge and computational approaches.^19−22^ Given the sophisticated nature of enzyme catalysis, multiple computational strategies and protocols have been developed in recent years for computational enzyme design.^21^ The evaluation of the conformational landscape of enzymes along distinct natural and DE evolutionary pathways has evidenced that the introduced mutations progressively tune the conformational ensemble, stabilizing key conformational states for the novel function.^4,6,11,21^ Of note is that the mutations introduced with DE are often located distal from the active site pocket, which, given the vast sequence space, are computationally challenging to predict.^21,23,24^ In addition to that, the computational prediction of which remote mutations can induce the desired population shift to favor the key conformational ensemble for novel functionality is an extremely difficult task.^21^ Our group has recently shown that active site and distal positions targeted by DE can be computationally identified through the coupling of MD simulations with cross correlation methods, such as the shortest path map (SPM).^21,25^ SPM has been applied for identifying DE mutations in the retro-aldolase, monoamine oxidase, and tryptophan synthase (TrpS) enzymes, suggesting its potential application for the rational design of enzyme variants.^21,25^

**Figure 1:**
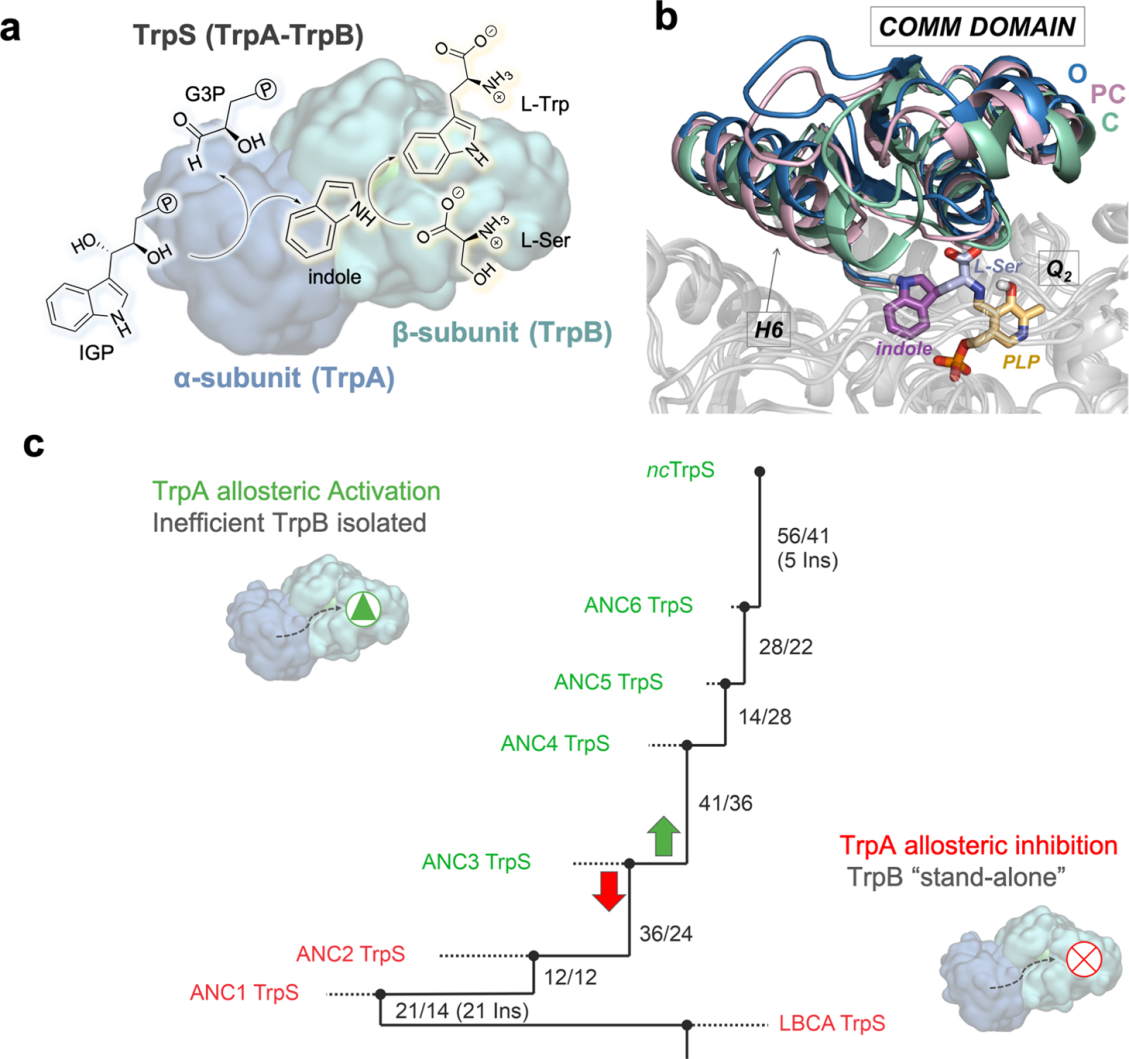
Overview of TrpS enzyme. (a) Functional unit of TrpS consists of a heterodimer, which is formed by TrpA (blue) and TrpB (green). TrpA catalyzes the cleavage of IGP to G3P and indole, which in TrpB reacts with activated l-Ser in a multistep mechanism to yield l-Trp (see Scheme S1). (b) Overlay of *pf*TrpS metastable conformations from previous computational exploration showing the transition of the COMM domain (residues 97–184) from an open (blue, O), to a partially closed (pink, PC) to a closed conformation (green, C). Highlighted are the α-helix H6 of the COMM domain (residues 174–164) and the reaction intermediate *Q*_2_ in the active site. The parts of the *Q*_2_ intermediate are colored depending on the respective precursor molecule (PLP cofactor in orange, l-Ser in blue, and indole in purple).^32^ (c) Phylogenetic tree shows the path from the LBCA TrpS over six intermediate nodes (ANC1 TrpS to ANC6 TrpS) to the extant *Neptuniibacter caesariensis* TrpS.^43^ Numbers next to each edge indicate the number of mutations accumulated in TrpA and TrpB with respect to the previous node. While LBCA TrpB gets deactivated by TrpA and exhibits stand-alone function, the allosteric effect of TrpA is reverted along the phylogenetic tree with a switch between ANC2 TrpS and ANC3 TrpS to an allosteric activation, as observed in extant *nc*TrpS.

TrpS is an excellent model system for studying allosteric properties. TrpS is a heterodimeric enzyme complex formed by α(TrpA) and β(TrpB) subunits in an αββα arrangement. The functional unit is formed by a TrpA, and an associated TrpB subunit (Figure 1a).^26,27^ TrpA catalyzes the retro-aldol cleavage of indole-3-glycerol phosphate (IGP) producing glyceraldehyde-3-phosphate (G3P) and indole, which diffuses along an internal tunnel toward the TrpB active site.^28^ TrpB is a pyridoxal phosphate (PLP) cofactor-dependent enzyme that catalyzes the production of l-tryptophan (l-Trp) by condensation of indole and l-serine (l-Ser) in a multistep reaction mechanism, which mainly comprises: (1) formation of a Schiff base intermediate (Ain) at the resting state by covalent attachment of the PLP cofactor to the catalytic lysine, (2) transamination with l-Ser, (3) indole coupling, and (4) formation of several quinonoid intermediates (*Q*) to finally release l-Trp. This complex multi-step mechanism involves multiple proton donor/abstraction steps assisted by the catalytic lysine (Scheme S1).^29^ Of relevance is the tight allosteric coupling between TrpA and TrpB along the catalytic itinerary.^30,31^ TrpA and TrpB catalyze different reactions that are synchronized (i.e., TrpA tunes the TrpB conformational ensemble and vice versa). This fine tuning of the conformational ensemble involves open-to-closed (O-to-C) transitions of the rigid COMM domain that forms a lid covering the TrpB active site (Figure 1b) and an active site loop of TrpA, as shown by X-ray and computational data.^27,32,33^ Given the tight allosteric communication exerted between subunits, both TrpA and TrpB are much less efficient when isolated, which hampers TrpB industrial application for non-canonical amino acid production.^34−39^ Arnold and co-workers addressed this limitation by applying DE to optimize activity of TrpB from the TrpS of *Pyrococcus furiosus* for stand-alone function (i.e., recovery of the catalytic activity in the absence of the allosteric protein partner TrpA).^34,35^ Interestingly, the most evolved variant (*pf*TrpB^0B2^) was even more efficient than the original *pf*TrpS complex (2.9-fold increase in *k*_cat_), and 5 out of its 6 mutations were located distal from the active site. This manifests that the recovery of activity exerted by the distal mutations is induced through allosteric effects.^34,35^ Intrigued by the allosteric regulation induced by distal mutations, we explored the conformational energy landscape of the *pf*TrpS enzyme complex, the *pf*TrpB isolated enzyme and the stand-alone *pf*TrpB^0B2^ evolved variant.^32^ Free-energy calculations revealed that the DE mutations in *pf*TrpB^0B2^ recovered the allosterically driven conformational ensemble of the p*f*TrpS complex, allowing the exploration of open, partially closed (PC), and closed conformations of the COMM domain, which is required for the multi-step catalytic pathway. The *pf*TrpB stand-alone activity was thus achieved though the recovery of the conformational ensemble present in the p*f*TrpS complex. In fact, the allosterically driven conformational ensemble was not only recovered but also improved as a higher stability of catalytically productive closed states was found in the case of *pf*TrpB^0B2^. This explained the *pf*TrpB^0B2^ superior activity with respect to the *pf*TrpS complex. In contrast, isolated *pf*TrpB showed a restricted COMM domain conformational heterogeneity and catalytically unproductive closed states. Careful analysis of the *pf*TrpS conformational ensemble through SPM correlation-based tools elucidated the enzyme pathways most contributing to the TrpS conformational dynamics, which interestingly included some important DE positions.^21,32^ This suggests that positions that were identified with the SPM method can potentially alter the conformational dynamics of the enzyme, and thus, its stand-alone activity. However, multiple positions are identified and there is a lack of information on which specific amino acid substitution should be introduced for achieving an efficient conformational ensemble for stand-alone function. This study aims to address these constraints by combining SPM with ancestral sequence reconstruction (ASR).^40−42^

ASR is an orthogonal *in silico* method to analyze functional transitions in enzyme evolution. In a previous work, we used ASR to reconstruct the TrpS phylogenetic tree and identified a shift in the allosteric modulation exerted by TrpA on TrpB activity.^43,44^ The analysis of the steady-state kinetic parameters of the last bacterial common ancestor (LBCA) revealed high stand-alone activity of LBCA TrpB and its allosteric inhibition in the presence of TrpA. Along the phylogenetic tree, this inhibition was gradually inversed toward allosteric activation existing in modern TrpB (Figure 1c).

This inversion of the allosteric effect exerted by TrpA on TrpB along the phylogenetic path provides a perfect starting point for an SPM-based approach. Specifically, we wanted to identify residues within the allosteric network of TrpB that are able to rescue the missing allosteric activation from TrpA and predict mutations that convey stand-alone function in the context of the catalytically inefficient ANC3 TrpB. To this end, we intended to explore the conformational ensemble of the stand-alone LBCA TrpB enzyme system and to identify key positions by means of our developed SPM correlation-based tool. Sequence comparison of the identified positions along the phylogenetic tree further reduces the number of potential mutations and provides the specific amino acid substitutions for stand-alone function. This approach decreases the experimental screening to one single mutant and includes the identification of both active site and distal mutations. Our study presents a computational approach that is not restricted to active site mutations and could be in principle applied in unrelated allosterically regulated systems. Moreover, it demonstrates that the challenge to identify distal positions impacting the catalytic activity of the enzyme can be ultimately addressed by exploring the conformational energy landscape of enzymes in combination with cross correlation, ASR, and multiple sequence alignment (MSA) bioinformatic tools.

## Results

### Reconstruction of Ancestral TrpS Conformational Ensembles

**Figure 2:**
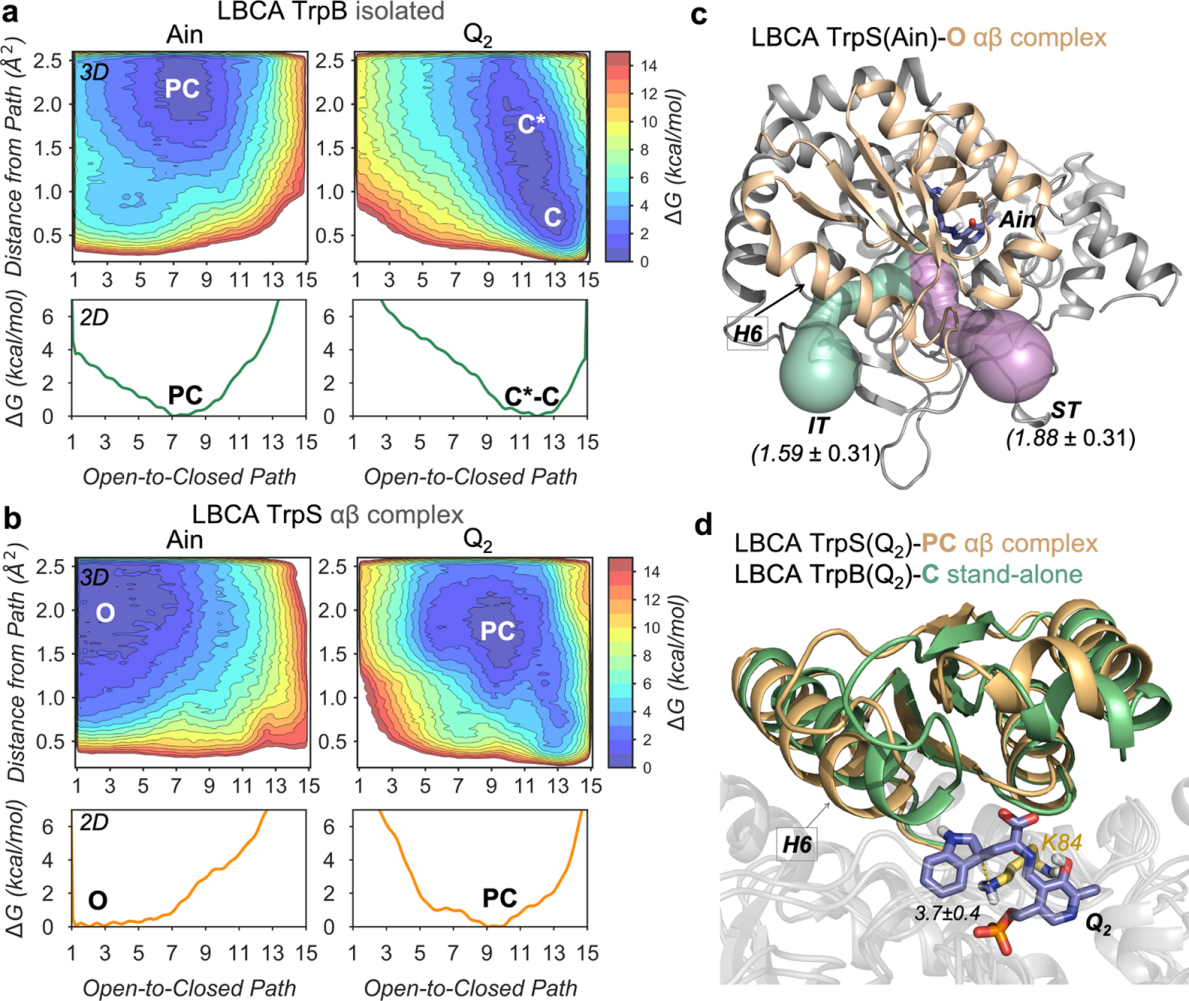
Computational exploration of the LBCA conformational ensemble. Free energy landscape (FEL) associated with the COMM domain O-to-C conformational transition of LBCA TrpB (a) and LBCA TrpS (b) at Ain and *Q*_2_ reaction intermediates. The *x*-axis corresponds to the progression along the reference O-to-C path generated from X-ray data, while the *y*-axis corresponds to the mean square deviation (MSD) distance from the reference path. Note that the deviated conformations (i.e., large *y*-axis values) encompassing the C state for LBCA TrpB at *Q*_2_ intermediate are labeled as C*. (c) Tunnels identified in the O state of LBCA TrpS at the Ain reaction intermediate that allow the access of l-Ser to the active site, computed with CAVER 3.0. The averaged bottleneck radii (in Å) for the internal TrpA-TrpB tunnel (IT, green) and the secondary tunnel (ST, violet) are also shown. (d) Overlays of the metastable conformations of the PC state of LBCA TrpS (orange) and the C state (green) of LBCA TrpB at *Q*_2_ reaction intermediate. The catalytic proton transfer distance (in Å) between the K84 (yellow) residue and the *Q*_2_ reaction intermediate (slate) is also represented.

As shown in previous studies, natural evolution has altered the need of TrpS to be allosterically regulated.^43^ As opposed to modern TrpB, the ancestral LBCA TrpB was found to operate less efficiently (in terms of *k*_cat_) in the presence of TrpA.^44^ The allosteric inhibition imparted by TrpA suggests that the ancestral TrpB in complex presents a more restricted conformational ensemble than that in isolation and is less efficient in accessing the catalytically productive conformational states required for enhanced activity.^32^ Interestingly, the affinity of LBCA TrpB toward the substrate l-Ser was enhanced in the heterocomplex form (Table S1). To provide the molecular basis for allosteric inhibition and higher affinity toward l-Ser in the heterocomplex TrpS and the stand-alone activity in isolation, we decided to computationally reconstruct the free-energy landscape (FEL) of LBCA TrpB in the presence and absence of TrpA (Figure 2). We employed metadynamics simulations to reconstruct the FEL associated with the O-to-C transition of the COMM domain (Figure S1) at the resting state [i.e., *E*(Ain)] and at the *Q*_2_ intermediate (i.e., quinonoid intermediate formed after indole coupling, see Scheme S1). The reconstructed FEL of the LBCA TrpB(Ain) in the absence of TrpA indicates that TrpB(Ain) mostly visits PC conformational states of the COMM domain (Figure 2a). This is altered in the presence of TrpA, which clearly induces a shift in the FEL stabilizing O states with similar deviations from the reference path (Figures 2a,b, on the left). At the resting state, C states are inaccessible for both systems. The analysis of the access tunnels to the active site for l-Ser binding through CAVER calculations (Figures 2c and S2) indicates that the PC conformational ensemble of the isolated LBCA TrpB has a substantially narrower tunnel bottleneck than the accessible O states of the complex. This finding indicates that the O conformational ensemble improves l-Ser accessibility to the active site, thus explaining the lower *K*_M_^L-Ser^ values displayed by the LBCA–TrpS complex.

More interesting is the fact that TrpA was found to inhibit the TrpB catalytic efficiency as isolated LBCA TrpB displays a 8.4-fold higher *k*_*ca*t_ value. As we show in our previous study,^32^ the catalytic activity of TrpS can be estimated by evaluating its ability to visit the allosterically driven conformational ensemble, especially the catalytically competent C states of the COMM domain. The catalytically relevant C conformational ensemble displays an efficient active site preorganization by means of optimized non-covalent interaction networks and short catalytic distances between the *Q*_2_ intermediate and the conserved catalytic K84 that acts as a proton acceptor. In particular, the H6 COMM domain α-helix was found to play an important role in the closure to form non-covalent interactions with the indole moiety of *Q*_2_. In the present work, the reconstructed FEL associated to the COMM domain O-to-C transition for LBCA–TrpB (Figure 2a,d) indicates that at the *Q*_2_ intermediate, the catalytically productive C conformational ensemble is indeed accessible for efficient catalysis. Structural characterization of this C conformational state shows catalytically productive COMM domain closure with appropriate K84-*Q*_2_ proton-transfer distances (Figures 2d and S3 and S4), as discussed above. Besides, within the broad energy minima of this C conformational state, largely deviated conformations along the *y*-axis are explored, labeled as C* in Figures 2a and S5. This feature provides LBCA TrpB some additional conformational heterogeneity needed for the catalytic cycle, as for instance for substrate binding and product release. Structural comparison of productive C and deviated C* conformations shows that the conferred heterogeneity is attributed to a certain flexibility of the H6 key dynamic element in the COMM domain (Figure S6). This evidences that LBCA TrpB has stand-alone properties derived from the exploration of stable catalytically competent C conformations in the absence of TrpA and its modest conformational heterogeneity. On the contrary, LBCA TrpA alters the conformational landscape of TrpB as it induces a shift toward PC conformations hampering the ability of the COMM domain to complete the O-to-C transition for achieving catalytically productive C states (Figure 2b). As expected, PC conformations of LBCA TrpB in the presence of TrpA do not exhibit a competent closure of the COMM domain; in particular, this is notorious for the H6 region. Besides, the K84-*Q*_2_ proton transfer distances are larger (Figures 2d and S3). In summary, our results indicate that the destabilization of the competent C LBCA TrpB ensemble is the main responsible factor for the allosteric inhibition exerted by the LBCA TrpA protein partner. It is worth mentioning that we estimated a similar effect (i.e., destabilization of the competent C ensemble) for the allosteric inhibition exerted by *pf*TrpA on the laboratory-evolved stand-alone *pf*TrpB^0B2^. Another interesting aspect of LBCA TrpB conformational dynamics is that a narrow set of states are sampled (i.e., no minima is found at O and PC), especially if compared with the previously studied allosteric *pf*TrpS complex and the laboratory-evolved stand-alone *pf*TrpB^0B2^ catalyst. This lower degree of conformational heterogeneity for exploring the complete O-to-C transition at *Q*_2_ intermediate observed for LBCA is compensated with a less restricted C conformational ensemble.

However, the lack of O states of the COMM domain at the *Q*_2_ intermediate for LBCA TrpB suggests a more rigid COMM as the reaction evolves, and an infrequent transition toward the O state, thus suggesting that product release might be rate limiting.

### Computational Identification of Distal Active Site Mutations for Stand-Alone Function

The mutations introduced along an evolutionary pathway progressively tune the conformational ensemble of enzymes toward a novel function.^4,6,11,21^ In this context, distal active site mutations have been shown to play a crucial role in natural and laboratory evolvability.^13,23^ Their prediction considering the vast protein sequence space that yields a targeted function is, however, an extremely challenging task in computational enzyme design.^21^ We have recently reported that molecular dynamics coupled to correlation-based tools are promising methodologies for the identification of both active site and distal positions targeted in non-rational laboratory evolution experiments.^21,25^ In particular, we successfully developed and applied the SPM method for identifying the enzyme pathways that most contribute to the conformational dynamics of the *pf*TrpS enzyme. Of relevance is that the identified positions coincide or form persistent non-covalent interactions with residues targeted in the laboratory evolution of the *pf*TrpS enzyme for stand-alone function.^32^ SPM identifies important positions for the enzyme conformational dynamics, thus reducing the potential number of mutational hotspots.

**Figure 3:**
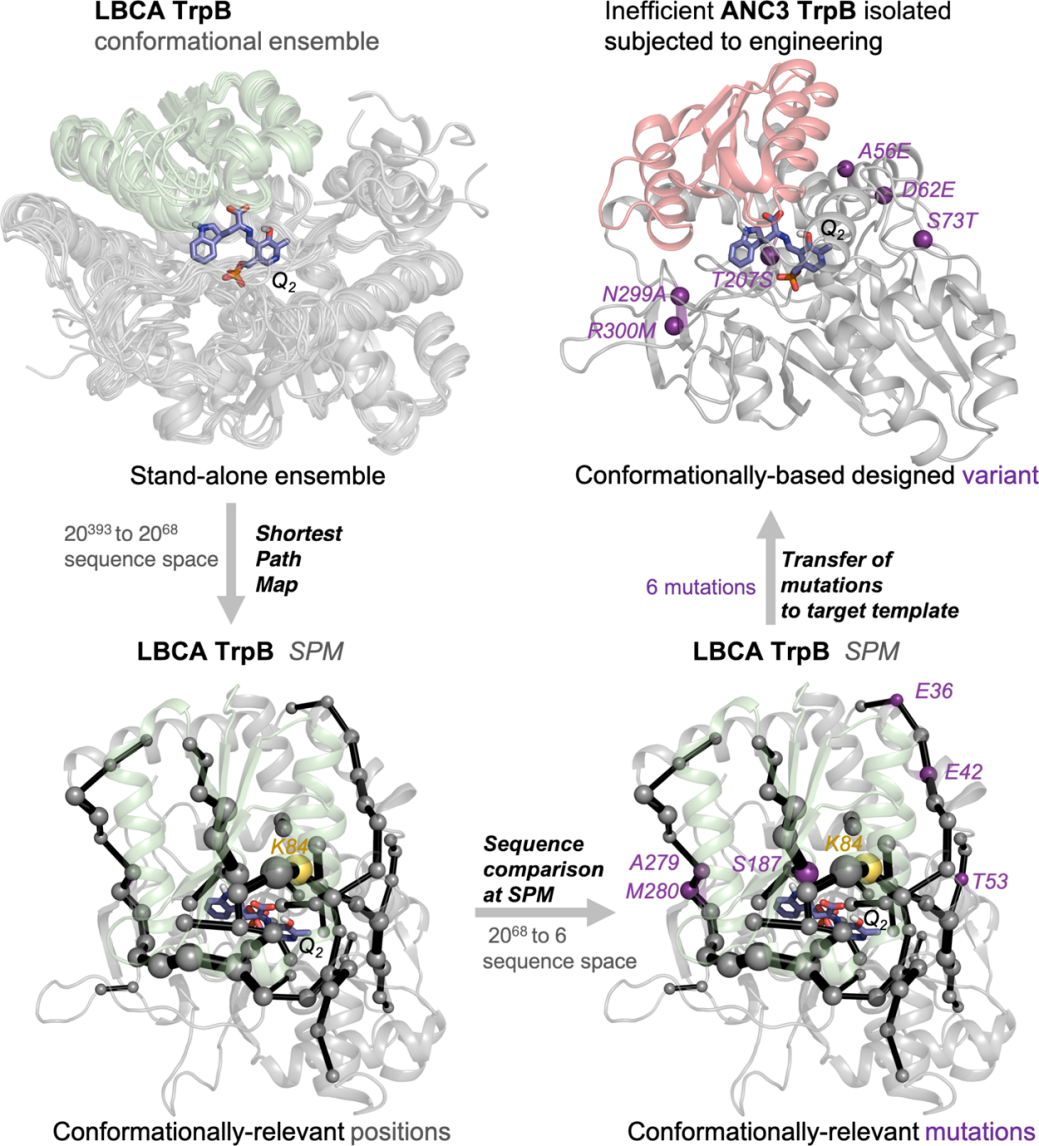
SPM-based computational workflow for SPM6 TrpB enzyme variant generation. By analyzing the conformational ensemble of the stand-alone LBCA TrpB with high catalytic activity (upper left ensemble) through the SPM, we identified positions (gray spheres, lower left structure) within allosteric pathways (black edges) in the enzyme that contribute most to the LBCA TrpB conformational dynamics in the *Q*_2_ intermediate. Thereby the size of each edge and node corresponds to the relevance for conformational dynamics. The catalytic residue K84 is highlighted in yellow. Excluding residues that do not participate in an allosteric pathway reduces the sequence space from 20^393^ to 20^68^ possible activity enhancing substitutions. Sequence comparison at the SPM positions between stand-alone LBCA TrpB and inefficient ANC3 TrpB reduces the sequence space to six mutations with respect to LBCA TrpB (lower right structure, purple residues), that were introduced into ANC3 TrpB (upper right structure, purple residues) and tested *in vitro*. Numbering of the residues is according to LBCA TrpB in the lower right panel and according to ANC3 TrpB in the upper right panel. Note that an insertion of 20 amino acids in ANC3 TrpB relative to LBCA TrpB leads to the shift in the residue numbering.

Inspired by our previous work on the TrpS ancestral reconstruction, we focused our computational approach on the ancestral ANC3 TrpB scaffold.^43,44^ This enzyme corresponds to the third node of the phylogenetic tree and exhibits reversion of allosteric inhibition toward activation along the evolution pathway (Figure 1c). In other words, ANC3 TrpB is the first enzyme that is allosterically dependent on TrpA, thus being highly inefficient as stand-alone catalyst (Table S2). The absence of TrpA decreases ANC3 TrpB activity about 30-fold in terms of *k*_cat_, suggesting a reduced conformational O-to-C ensemble. Given the success of SPM in identifying key positions for the enzyme conformational dynamics, we decided to apply our computational methodology to confer ANC3 TrpB improved stand-alone activity. Our initial reference protein was LBCA TrpB, as it exhibits stand-alone activity thanks to its ability to adopt stable and efficient closed states of the COMM domain. The SPM analysis of the LBCA TrpB identified numerous possible hotspots that potentially regulate the enzyme conformational dynamics (68 out of 413 residues, i.e., 18% of the full-length enzyme). This number is too large for an efficient redesign of ANC3 TrpB, as it is unclear which positions should be targeted and which substitutions should be introduced to establish stand-alone function. Similarly, sequence comparison between LBCA TrpB and ANC3 also identifies many potential hotspots (42 out of 393 residues, i.e., 11% of the full-length enzyme). We solved this problem by analyzing the sequence conservation between LBCA TrpB and the targeted ANC3 TrpB system for the 68 SPM positions (see the workflow followed in Figure 3). This combined approach based on SPM, ASR, and sequence conservation analysis successfully reduced the sequence space to only six positions.

### New SPM-Based ANC3 TrpB Variants for Stand-Alone Function

**Figure 4:**
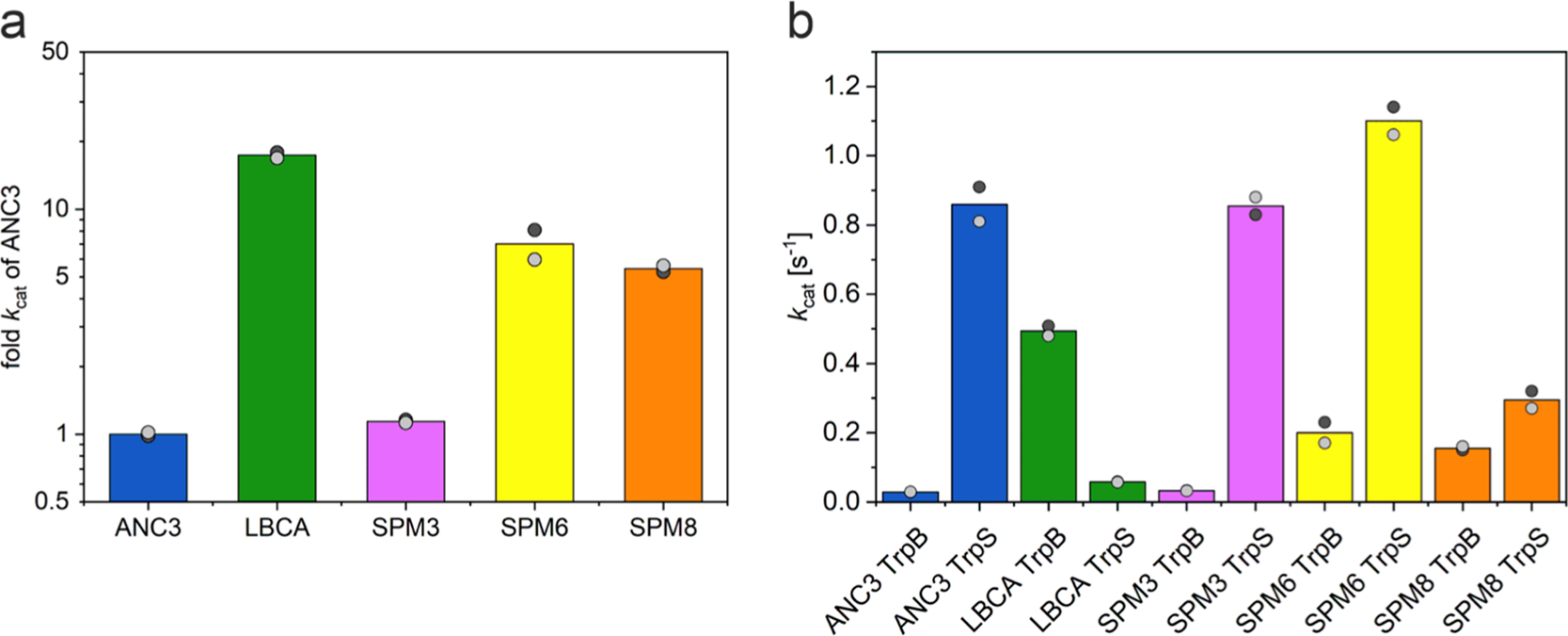
Illustration of the TrpB kinetic characterization. (a) TrpB stand-alone activities (in terms of *k*_cat_) for LBCA, SPM3, SPM6, and SPM8, shown as multiples of the ANC3 activity (logarithmic scale). While the reference stand-alone LBCA TrpB is 17.4-fold more active than ANC3 TrpB, the new TrpB designs SPM3, SPM6, and SPM8 are 1.1-fold, 7-fold, and 5.4-fold more active than ANC3 TrpB. (b) Activity differences (in terms of *k*_cat_) between isolated and TrpA-complexed ANC3, LBCA, SPM3, SPM6, and SPM8 TrpB enzymes. TrpS complex formation leads to an increase in the catalytic activities of ANC3, SPM3, SPM6, and SPM8 TrpB enzymes by factors of 30.2, 26.3, 5.5, and 1.9, and a decrease for LBCA TrpB by a factor of 8.4. The bar height represents the average value from two independent measurements, which are shown as gray dots. All catalytic constants are listed in Tables S2–S5.

The application of the SPM method coupled to sequence comparison between two variants exhibiting rather high (LBCA TrpB) or low (ANC3 TrpB) stand-alone function reduced the SPM library to only six specific mutations in ANC3 TrpB: A56E, D62E, S73T, T207S, N299A, and R300M. This ANC3 variant was termed SPM6 TrpB. Interestingly, none of the mutations is located in the COMM domain, and five out of six mutations are located far away from the active site (*ca*. 18–29 Å), among which N299A and R300M are near the TrpA-TrpB protein interface, and only S73T is located at the active site pocket (Figure 3). The computational screening of ANC3 TrpB, the ANC3 TrpS, and the SPM6 TrpB enzyme variant by means of conventional molecular dynamics simulations suggests that both SPM6 TrpB and ANC3 TrpS are able to retain the closed conformation of the COMM domain. In contrast, isolated ANC3 TrpB explores additional non-productive conformations (Figure S7). This fast screening computational protocol indicates a rather low stability of the C state of the COMM domain in isolated ANC3 TrpB, which is in line with its low stand-alone catalytic activity. These computational insights encouraged us to experimentally test the SPM6 enzyme variant. As shown in Figure 4a, the turnover number of SPM6 TrpB with respect to ANC3 TrpB is enhanced by almost one order of magnitude (7-fold increase in *k*_cat_). The catalytic efficiencies *k*_cat_/*K*_M_ for both, indole and l-Ser are improved by 4-fold and 7-fold (Tables S2 and S3). It is worth emphasizing that a similar fold increase in stand-alone catalytic activity, as obtained for SPM6, was achieved in *pf*TrpB by means of multiple rounds of laboratory evolution.^34^ Our SPM-based computational approach therefore provides the same order of improvement in stand-alone activity but by only testing one single rationally designed variant. However, the maximum observed stand-alone catalytic potential, as displayed by LBCA TrpB (17.4-fold more active than ANC3 TrpB; Figure 4a) was not matched.

Another interesting aspect to evaluate is whether the SPM6 mutations have an impact in the allosteric modulation exerted by TrpA. The catalytic activity of the ancestral ANC3 TrpB increases 30.2-fold in terms of *k*_cat_, thanks to the TrpA-triggered allosteric activation. Remarkably, the introduction of LBCA residues into ANC3 TrpB confers increased stand-alone activity in SPM6, while still retaining some TrpA allosteric activation (the activity of SPM6 TrpB is enhanced by 5.5-fold in the presence of TrpA; Figure 4b). This indicates that the SPM6 distal mutations tune the O-to-C conformational ensemble of SPM6 TrpB through long-range intra-subunit allosteric effects, but these changes in the conformational landscape do not prevent TrpA inter-subunit allosteric activation. In fact, the combination of both intra-subunit and inter-subunit allosteric effects leads to a catalytic activity of the SPM6 TrpS complex that slightly exceeds the catalytic activity of the ANC3 TrpS complex (i.e., 1.3-fold increase, Figure 4b).

**Figure 5:**
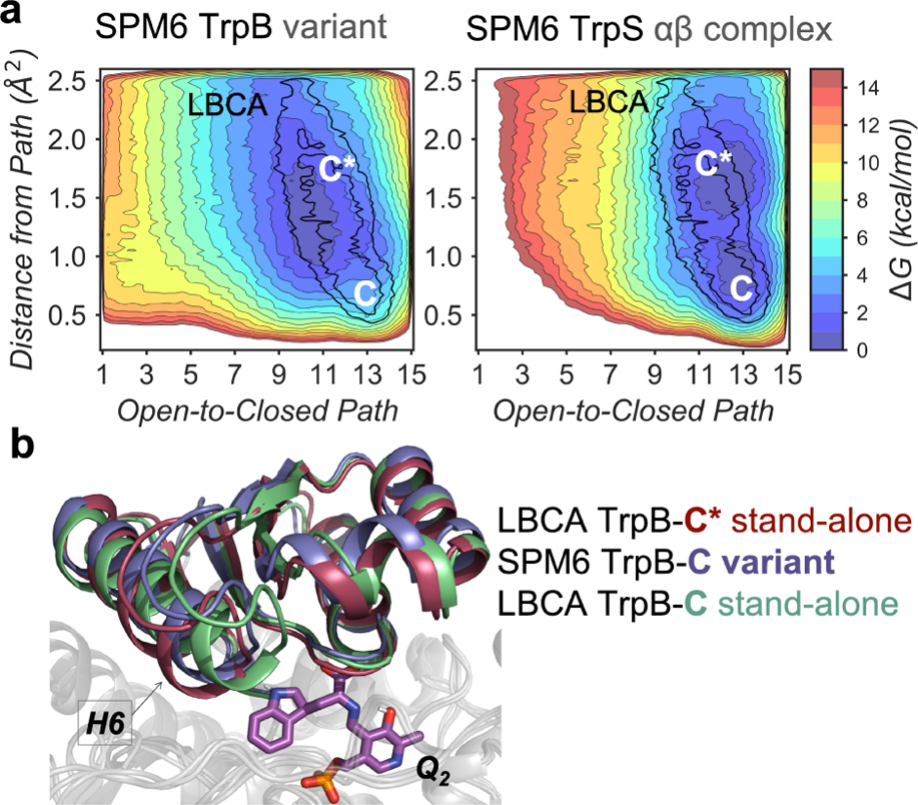
Computational exploration of the SPM6 conformational ensemble. (a) FEL associated with the COMM domain O-to-C conformational transition of SPM6 TrpB and SPM6 TrpS at *Q*_2_ reaction intermediate. For both systems, the LBCA TrpB local energy minima of the closed (C*-C) state is projected over the FEL and represented in black. The *x*-axis corresponds to the progression along the reference O-to-C path generated from X-ray data, while the *y*-axis corresponds to the MSD distance from the reference path. (b) Overlays of the metastable conformations of the deviated closed (C*) state of LBCA TrpB (garnet), the closed (C) state of SPM6 TrpB, and the productive closed (C) state of LBCA TrpB at *Q*_2_ reaction intermediate.

To further investigate the effects of distal mutations introduced in SPM6 and the TrpA allosteric activation exerted on the SPM6 TrpB variant, we performed additional metadynamics simulations (see methods in Supporting Information). In particular, we reconstructed the COMM conformational landscape for ANC3 TrpB, SPM6 TrpB, and their respective heterocomplexes (i.e., ANC3 TrpS and SPM6 TrpS, see Figures 5 and S8). Similarly to what we observed in *pf*TrpB,^32^ both ANC3 and SPM6 TrpB variants mostly explore catalytically unproductive C conformational states and display a rather restricted conformational ensemble at the *Q*_2_ intermediate. However, the distal mutations introduced in SPM6 TrpB variant partially recover the conformational heterogeneity of LBCA TrpB, as a wider energy minimum for the C state is observed (i.e., larger deviation along the *y*-axis, see Figures 2a and 5a). This deviation confers some additional flexibility to the COMM domain of SPM6 TrpB, which could facilitate both, substrate binding and product release, thus explaining its 7-fold higher stand-alone activity. Still, the introduced mutations are not able to completely shift the C conformational ensemble toward more catalytically productive C conformational states. Interestingly, the SPM6 TrpB-C state lays in between productive C and deviated C* states of the stand-alone LBCA TrpB enzyme (Figure 5b). This evidences that some extra H6 COMM domain flexibility and stabilization of the productive C state would be required to further promote stand-alone activity in SPM6 TrpB. In contrast, the presence of TrpA in the ANC3 and SPM6 systems allows the exploration of catalytically productive C states. This is especially the case for SPM6 TrpS that exhibits a more stable C conformational ensemble, in line with its superior catalytic activity. Additionally, a higher conformational heterogeneity in the presence of TrpA is observed for both heterocomplexes, as they explore the catalytically productive C state and the more deviated C* conformations (Figures 5, S8 and S9).

### Additional SPM-Based Strategies for ANC3 TrpB Stand-Alone Function

To further test the SPM predictive power and the robustness of the strategy followed so far, we additionally targeted two other SPM-based approaches. In the first one, we followed the same workflow as for the SPM6 design but used instead of LBCA TrpB the isolated ANC2 TrpB (Figure 1) as stand-alone reference protein for the SPM pathway analysis. After identifying the SPM in ANC2 TrpB and subsequent sequence comparison between ANC2 TrpB and ANC3 TrpB, we identified 3 SPM positions and the corresponding amino acid exchanges in ANC3 TrpB: S73T, N299S, and R300M. The resulting variant was termed SPM3. This reduced number of non-conserved SPM positions makes sense since ANC2 and ANC3 are closer in the phylogenetic tree than LBCA and ANC3.

In the second approach, we conducted an SPM analysis on the ANC3 TrpS complex. The rationale behind taking this complex as a reference was that, while isolated ANC3 TrpB is poorly active and its allosteric communication is likely truncated, complexation with TrpA leads to high activity (Figure 4b and Table S2) and a restored allosteric network. After identifying allosterically relevant SPM positions within ANC3 TrpS, we again compared the ANC3 TrpB sequence to LBCA TrpB to identify mutations that lead to a stand-alone catalyst. Following this protocol, we identified the variant SPM8, which contains two extra exchanges, as compared to SPM6: R53N and M187I, where R53N is far away from the active site and M187I is located at the H6 helix of the COMM domain. It should be noted that the three positions of SPM3 and six out of eight of SPM8 were previously identified in SPM6. Following the same computational MD-based screening protocol, the SPM3 and SPM8 TrpB variants were analyzed. The results suggested a rather high stability of the C state of the COMM domain and thus enhanced activity (Figure S7). Both the catalytic TrpB activities of SPM3 and SPM8 were next tested experimentally. SPM8 TrpB exhibited a similar activity enhancement (5.4-fold in terms of *k*_cat_) as SPM6 TrpB, whereas a quite modest enhancement (1.1-fold) was obtained for SPM3 TrpB, in line with the reduced number of mutations (Figure 4a). Regarding the inter-subunit allosteric effects exerted by TrpA, SPM3 and SPM8 variants also showed TrpA allosteric activation. In particular, SPM3 showed a similar degree of *k*_cat_ increase when in complex as ANC3 (26.3-fold), while the SPM6 (5.5-fold) and the SPM8 (1.9-fold) enzyme variants present a reduced predisposition to the TrpA allosteric activation (Figure 4b).

### New Approach for Enzyme Design: Combined SPM-Based Detection of Conformationally Relevant Positions and Multiple Sequence Alignment Tools

As we have shown above, the combination of correlation-based tools and sequence comparison between the target enzyme and stand-alone template has dramatically reduced the sequence space: from 393 positions for the full-length TrpB protein to only six specific mutations that resulted in enhanced stand-alone function. While this new approach might be potentially used for computationally converting enzyme functionalities within the same family or for enhancing some pre-existing side promiscuous activities, its applicability could be further expanded if no reference template was required. To further explore this point, we focused on comparing the generated SPMs from homologous TrpB enzymes and applied multiple sequence alignment (MSA) tools.

The comparison of the conformationally relevant positions, as identified by SPM for LBCA TrpB and the *pf*TrpS complex,^32^ reveals that many of the detected positions are shared (Table S6 and Figure S10). In particular, we find that 48 out of 68 LBCA TrpB SPM residues are also detected in the SPM of the modern *pf*TrpS complex (i.e., 71% of common conformationally relevant amino acids). This is suggesting that many of the conformationally relevant positions are shared between homologous enzymes, and thus even in the absence of the stand-alone LBCA reference, many of the key SPM positions would have been detected by combining the results of these two independent analyses. This suggests that the SPM analysis of multiple TrpS complexes might converge to a low set of common conformationally relevant residues, which then can be tested experimentally. Importantly, five out of the six targeted positions for SPM6 variant generation are contained in the mentioned common SPM set of 48 amino acids, indicating that hardly any information would be lost by such an integrative SPM approach.

The MSA of 52 extant TrpB sequences from our previous study^43,44^ also provides interesting new insights. MSA identifies 144 residues with a conservation higher than 90% (Table S6). Remarkably, none of the six targeted residues for SPM6 generation is included in this highly conserved set, indicating that MSA and SPM are complementary techniques when trying to identify crucial positions. Along these lines, 12 out of 48 SPM positions that are shared between LBCA TrpB and *pf*TrpB present a conservation score lower than 70%, and indeed five of the SPM6 positions are contained in this set of 12 low conserved residues. Therefore, the application of the SPM methodology shows that non-conserved residues, which would be missed by an MSA-based analysis, can play a crucial role for allostery and stand-alone function. However, as shown in our previous work, the identification of conserved residues within different groups of enzymes exhibiting either allosteric activation or inactivation can also be successful.^43^ Remarkably, the best variants of each approach harbored four mutations (ANC3 AM4)^43^ and six mutations (SPM6) with only one common mutation, namely T207S. Therefore, there is hardly any overlap between the identified residues by the two approaches, demonstrating that they are clearly complementary.

## Discussion

Allosteric regulation is a central biological process focused on the functional connection between distinct sites on either a single biological entity or among complex multimeric structures.^9,13,45^ This regulation of enzymatic function is not limited to effector or protein partner binding, as similar effects have been observed by covalent attachment or by introducing mutations located at distal positions of the enzyme active site.^9,10,13^ The elucidation of the underlying mechanism and forces that drive allosteric regulation has the enormous potential of identifying key positions for regulating enzymatic function, which could be exploited in enzyme design.^21^ The present study indeed demonstrates that positions distal from the active site, regulating the allosterically driven conformational ensemble and thus the enzyme activity, can be successfully identified by means of correlation-based tools and sequence comparison analysis. Given the vast sequence and conformational space, the identification of remote positions from the active site impacting enzymatic function, is an extremely difficult task in the computational enzyme design field. Apart from that, the identification of the specific amino acid substitutions that optimize the enzyme conformational ensemble for a targeted enzyme function is challenging. Our study focuses on the engineering of stand-alone function taking advantage of the substantial allosteric contributions that distal mutations were exerting on the laboratory-evolved variants.^32,34,35^ The exploration of the FEL of the ancestrally reconstructed LBCA TrpS in complex and as stand-alone catalyst (LBCA TrpB), together with our previous findings^32^ on the wild-type *pf*TrpS complex, isolated *pf*TrpB, and laboratory-evolved *pf*TrpB^0B2^ has elucidated the conformational ensemble that a stand-alone catalyst has to display for being efficient. This information is pivotal for fine-tuning the conformational ensemble and progressing toward the targeted enzyme design goal. We find that LBCA TrpB naturally adopts a stable catalytically productive COMM domain closure, which is hampered by the presence of the LBCA TrpA protein partner. LBCA TrpA therefore induces an allosteric inhibition of LBCA TrpB activity, which contrasts with the TrpA allosteric activation usually found in modern TrpB enzymes. In this study, we exploit the conformational heterogeneity and intrinsic ability of LBCA TrpB to efficiently stabilize catalytically competent COMM domain closed conformations when isolated (which are both crucial for stand-alone properties) and utilize a combined SPM and ASR approach for conferring stand-alone function. In particular, we apply our SPM method to identify the enzyme pathways and positions that contribute most to the ancestrally reconstructed LBCA TrpB conformational dynamics. We hypothesized that these conformationally relevant SPM positions could be potential hotspots for tuning the conformational ensemble of TrpA-dependent TrpB enzymes. The reconstruction of the phylogenetic tree from LBCA TrpS to the modern *nc*TrpS provided an intermediate variant ANC3 TrpB, which exhibits a high allosteric activation from ANC3 TrpA (i.e., ANC3 TrpB is highly inefficient when isolated). The application of SPM to LBCA TrpB reduced the sequence space from 393 to 68 SPM positions, suggesting that *ca*. 18% of the residues play a conformationally relevant role. However, this still leads to a massive amount of enzyme variants to screen. Similarly, 42 potential hotspots are identified by simply comparing LBCA and ANC3 TrpB sequences. Interestingly, the analysis of sequence conservation at the identified SPM positions between LBCA and ANC3 TrpB reduced this large number to only six positions. This approach assumes that the transfer of the non-conserved conformationally relevant SPM mutations from the LBCA to the targeted ANC3 TrpB template will enhance the enzyme conformational heterogeneity and induce the stabilization of the catalytically relevant closed state of the COMM domain. It is worth mentioning that among these six mutations, five are distal from the active site and none is included in the COMM domain.

The experimental evaluation of SPM6 showed that the introduced mutations boosted the stand-alone catalytic activity of the inefficient isolated ANC3 TrpB enzyme near one order of magnitude. The enhancement by only testing this single variant is comparable to that observed for the laboratory evolved *pf*TrpB^0B2^ after three rounds of DE, which involved the screening of *ca.* 3080 variants.^34^ The observed enhancement of ANC3 TrpB stand-alone activity still does not completely recover the 100% of the activity displayed by the ANC3 TrpS complex. The newly designed variant SPM6 enhances the low initial 3% activity displayed by ANC3 TrpB up to a *ca.* 23% recovery. It should be also mentioned that the SPM6 design is based on the template scaffold of LBCA TrpB, whose catalytic activity is lower than that of the ANC3 TrpS complex (LBCA TrpB activity is *ca.* 58% that of ANC3 TrpS). In the case of the DE *pf*TrpB^0B2^ enzyme variant, 300% of activity recovery was observed.^34^

The partial recovery observed for SPM6 is in part due to the dramatic loss of activity displayed by ANC3 TrpB in the absence of TrpA (97% of activity loss), which is more moderate in *pf*TrpB (69%). These numbers indicate that the total recovery of ANC3 activity is more demanding from an engineering point of view and suggest that the newly generated SPM6 variant still presents some predisposition toward TrpA regulation. Our simulations showed that the distal mutations introduced in the SPM6 variant successfully enhanced the stand-alone activity of ANC3 TrpB activity through intra-subunit allosteric effects, which slightly enhanced the COMM domain conformational heterogeneity by enlarging the population of the closed state. However, the introduced mutations in the SPM6 variant did not completely free TrpB from the inter-subunit allosteric regulation exerted by TrpA, as further heterogeneity and the stabilization of the catalytically competent closed state are required. To our surprise, SPM6 in complex with TrpA showed the most efficient turnover tested in this work. The reconstruction of the conformational landscape of SPM6 TrpB in complex with TrpA indicated that the increased catalytic activity is attributed to a higher conformational heterogeneity and the stabilization of the catalytically competent closed conformation of the COMM domain. Altogether these results indicate that the combination of intra- and inter-allosteric effects can operate synergistically to successfully tune the O-to-C conformational ensemble and achieve high catalytic efficiencies.

Another secondary insight gained from this work comes from the analysis of how the TrpS conformational landscape is altered and conserved along the natural evolutionary pathway. The exploration of the conformational ensemble and the identification of the key conformationally relevant SPM positions of LBCA, ANC2, and ANC3, and their comparison with the previously studied modern *pf*TrpS revealed that the main allosteric pathways are not significantly altered along evolution. Indeed, the comparison of the generated SPM paths for the different enzymes reveals a rather high number of shared positions, thus suggesting similar TrpB correlated motions among ancestral and extant variants. This suggests that even in the absence of a stand-alone reference, the most important positions for the conformational dynamics of the enzyme could be in principle identified. The analysis of the conservation of the SPM conformationally relevant positions through MSA tools also evidenced that the targeted SPM positions for the SPM6 generation present a rather low conservation score. This demonstrates the high complementarity of SPM and MSA as the targeted SPM positions would have been missed if only MSA was applied. Interestingly, only one of the positions identified in SPM6 (i.e., T207S) was also found in our previous study based on MSA,^43^ thus further highlighting that SPM and MSA are complementary approaches that can be used to identify allosterically relevant residues. Our findings indicate that the detection of conformationally relevant positions through SPM, specially if applied in ancestral enzymes, which lack some conformational restrictions, corresponds to a successful approach for creating active TrpB variants, either for improved stand-alone or in complex function. It also evidences that conformational heterogeneity, and in particular, the use of ancestral conformationally rich scaffolds corresponds to a successful strategy for designing the desired enzymatic functions.^42,46^ The success of the utilized SPM and MSA computational approach in this particular case could be attributed to the existing allosteric pathways in TrpB. Still, the fact that in many DE experiments key distal mutations impacting catalytic activity are found is suggesting the existence of allosteric communication between distinct protein sites. Indeed, it was hypothesized that allostery might be an intrinsic property of all dynamic (non-fibrous) proteins.^8^ These observations are encouraging for the development of more general SPM–ASR–MSA approaches for computational enzyme design.

## Conclusions

The SPM–ASR–MSA approach presented in this work highlights that the exploration of the enzyme conformational ensemble is essential for identifying key conformationally relevant sites and dramatically reducing the sequence space to only a few mutations. The detection of the key conformationally relevant positions and the combined analysis of its conservation along ancestral phylogenetic trees and/or extant enzyme homologues harbors meaningful information for solving the current challenge in computational enzyme design of distal active site prediction for enhanced function.

## Supporting Information Available

Computational and experimental methods; O-to-C path of collective variables; tunnel access at Ain reaction intermediate for the L-Ser substrate; detailed active site view; overlays of the metastable conformations of the closed states at *Q*_2_ intermediate; projection of the conformations that correspond to the local energy minima coordinates; overlays of the metastable conformations; population analysis of the conventional molecular dynamics simulations; computational exploration of the ANC3 conformational ensemble; computed SPM for the different TrpB systems; calculation of SPM of LBCA using different thresholds; 2D FELs associated with the COMM domain; differences in energy between selected regions of the FEL surface along the metadynamics simulations; steady-state enzyme kinetic parameters of LBCA TrpB, ANC3 TrpB, SPM6 TrpB, SPM8 TrpB, and SPM3 TrpB; and conservation level of TrpB residues in extant organisms (PDF)
