# Supplementary material for: In Silico Identification and Experimental Validation of Distal Activity-Enhancing Mutations in Tryptophan Synthase
Source: ACS Catal. 2021 Oct 28;11(21):13733–43. doi: 10.1021/acscatal.1c03950 (PMC8576815; doi:10.1021/acscatal.1c03950)
Supplement: Supplementary file 1 — cs1c03950_si_001.pdf [file cs1c03950_si_001.pdf]

In silico identification and experimental validation of distal activity-enhancing mutations in tryptophan synthase

Miguel A. Maria-Solano,[a,d]#* Thomas Kinateder,[b]# Javier Iglesias-Fernández,[a,e] Reinhard Sterner,[b]* and Sílvia Osuna[a,c]*

[a] CompBioLab group, Institut de Química Computacional i Catàlisi (IQCC) and Departament de Química, Universitat de Girona, Girona, 17003, Spain

[b] Institute of Biophysics and Physical Biochemistry, Regensburg Center for Biochemistry, University of Regensburg, Universitätsstrasse 31, Regensburg, 93053, Germany

[c] ICREA, Pg. Lluís Companys 23, Barcelona, 08010, Spain

[d] Global AI Drug Discovery Center, College of Pharmacy and Graduate School of Pharmaceutical Science, Ewha Womans University, Seoul, 03760, Republic of Korea

[e] Nostrum Biodiscovery, Carrer de Baldiri Reixac, 10–12, Barcelona, 08028, Spain

#: These authors contributed equally to the work

Corresponding Authors

*miguel.mariasolano@ewha.ac.kr
*silvia.osuna@udg.edu
*Reinhard.Sterner@ur.de

Supporting information

SI Table of Contents

Computational methods……………………………………………………………………………...S2

Experimental methods…………………………………………………………………………….S6

SI Tables………………………………………………………………………………………...S7

SI Schemes……………………………………………………………………………………..S25

SI Figures……………………………………………………………………………………S26

References……………………………………………………………………………………..S38

Computational methods:

Molecular Dynamics simulations. *System preparation.* The crystal structure of the LBCA TrpS complex (LBCA TrpA + LBCA TrpB), with PDB accession code 5ey5 was used as starting structure. The missing X-Ray regions were added using Modeller web server. The ANC3 TrpS complex was constructed by homology modelling. The ANC2 TrpB, the SPM3, SPM6 and SPM8 variants were generated from the ANC3 TrpB template using the mutagenesis tool included in Pymol (http://www.pymol.org/). Isolated TrpB enzymes were generated by manually removing its corresponding TrpA subunit. MD parameters for the reaction intermediates for TrpA (IGP, GP3) and TrpB (Ain and Q_2) were generated with the antechamber and parmchk modules of AMBER16[1] using the general amber force-field (GAFF). The partial charges (RESP model) were set to fit the electrostatic potential generated at the HF/6-31G(d) level of theory using the Gaussian09 software package. The different reaction intermediates were placed in the TrpA and TrpB subunits by alignment to available X-ray structures. For the simulations of TrpS complexes, two different combinations of bound substrates were used: in the first simulations IGP was introduced in TrpA subunit, while Ain intermediate was bound in TrpB; in the second set, G3P was introduced in TrpA, while Q_2 intermediate was placed in TrpB. A total of 11 systems were generated: 4 TrpS complexes (LBCA TrpS with IGP-Ain and GP3-Q_2 intermediates, ANC3 TrpS with GP3-Q_2 intermediates and SPM6 TrpS with GP3-Q_2 intermediates), 6 isolated TrpB enzymes (LBCA TrpB at Ain and Q_2 intermediates, and ANC3 TrpB, ANC2 TrpB, SPM3 TrpB, SPM6 TrpB and SPM8 TrpB at Q_2 intermediate).

Molecular dynamics simulation details. All enzyme structures were filled with buffer in a pre-equilibrated cubic box of 10 Angstrom using the TIP3P water model and neutralized by the addition of explicit counterions (Na^+ and Cl^-) using the AMBER 16 leap module. All subsequent calculations were performed using a modification of the amber99 force field (ff14SB). A two-stage geometry optimization approach was performed. The first stage minimizes the positions of solvent molecules and ions imposing positional restraints on solute by a harmonic potential with a force constant of 500 kcal mol^{-1}Å^{-2}, and the second stage is an unrestrained minimization of all the atoms in the simulation cell. All systems were gently heated using seven 50 ps steps, incrementing the temperature 50 K each step under constant-volume and periodic boundary conditions. Hydrogen bonds were set to fixed lengths using the SHAKE algorithm. Long-range electrostatic effects were modeled using the particle-mesh-Ewald method. An 8 Å cutoff was applied to Lennard-Jones and electrostatic interactions. Decreasing harmonic restraints were applied

to the protein (210, 165, 125, 85, 45, 10 kcal $mol^{-1}Å^{-2}$) during the thermal equilibration, with the Langevin scheme used to control and equalize the temperature. The time step was kept at 1 fs during the heating stages, allowing potential inhomogeneities to self-adjust. Each system was equilibrated without restrains for 20 ns with a 2fs timestep at a constant pressure of 1 atm. After the systems were equilibrated in the NPT ensemble, a production run MD simulation was performed for each system in the NVT ensemble and periodic-boundary conditions. Production runs were performed with the AMBER16 software. For the systems subjected to the fast MD-based screening (ANC3 TrpS, ANC3 TrpB, ANC2 TrpB, SPM3, SPM6 and SPM8 at Q_2 intermediate) a production run of 3 independent replicas 500 ns each (i.e. 1.5 μs accumulated time for each system) was performed at 333 K.

Metadynamics simulations. *Path of collective variables.* For the LBCA systems we were interested in obtaining the Free energy landscape as a function of the COMM domain Open-to-Closed transition. To that end, we followed the same protocol employed in our previous work where we applied well-tempered metadynamics simulations with a path of collective variables to construct the FEL for the *pf*TrpS complex, the isolated *pf*TrpB and the evolved variant *pf*TrpB^{0B2}.[2] The LBCA FEL obtained here is therefore directly comparable to the previous FEL explorations of *pf*TrpS.

Metadynamics[3] consists of the addition of external energy potentials at a regular number of MD steps. In particular, the external potentials are added to a selected degree of freedom, often referred to as collective variables (CVs). After sufficient simulation time, metadynamics provides a reliable estimation of the underlying free energy as a function of the CVs by summing the external potentials added along the simulation. The choice of the collective variables (CV) represents a critical step in metadynamics simulations. Ideally, for the TrpS system, the CVs should: (1) clearly distinguish between open and closed states, as well as the intermediate states, (2) describe the slow COMM domain conformational exchange and (3) their number should not be too large. Here we used a path of collective variables approach that describes the allosteric open-to-closed conformational transition. The path of collective variables was generated with the morph Gromacs tool, including only the α-carbons of the most relevant dynamic elements of the protein: the COMM domain (residues 97-184) and a loop region located at the base of the COMM domain (residues 282-305), see **Figure S1**. The tool generates a selected number of interpolated structures between an initial and final state provided. In this case, the path was composed of a set of 15 structures including the initial state (i.e. open state, PDB ID: 1WDW) and the final state (i.e. closed state, PDB ID: 3CEP) obtained from X-ray data. We restricted the protein atoms included in the path to alleviate the path dimensionality while accounting for the essential descriptors of the allosteric transition.

Note that, in this work, two paths of collective variables were considered. Specifically, the first collective variable (x axis in our 3D free energy plots) corresponds to the progression along the path, encompassing the 15 conformations generated from open (x value = 1) to closed (x value = 15) structures, see **Figure S1**. The second CVs (y axis in our 3D free energy plots) represents the mean square deviation (MSD), in $Å^2$, from the reference path provided. Note that the first CV, the progression along the path, has no units since it corresponds to arbitrary values

between 1 (open) and 15 (closed), which are dependent to a particular set of α-carbon atom coordinates. It is worth mentioning that the linear interpolation between open and closed structures may result in non-physical conformations of the protein, a limitation that is overcome thanks to the second collective variable included, i.e. the deviation from the reference path of structures.

When constructing the path of conformations for the metadynamics calculations there is a key aspect one should consider. That is, an optimal path requires approximately equal spacing between the neighboring frames. This can be observed by representing the RMSD values as a function of the 15 conformations of the path. Once verified, a suitable λ parameter can be computed by multiplying the inverse of the mean square displacement between successive frames times 2.3. In our calculations we used a λ value of 80. Given the high 3D structural similarity between LBCA and the modern enzymes as shown by the available X-ray data, the path of conformations previously generated for the modern *pf*TrpB enzyme perfectly matches that obtained for LBCA.

Well-tempered and Multiple walkers. We used the PLUMED2 software package[4] and GROMACS 5.1.2 code[5] to perform the metadynamics simulations. First, we carried out a metadynamics simulation for the 8 systems targeted: 4 TrpS complexes (LBCA TrpS with IGP-Ain and GP3-Q_2 intermediates, ANC3 TrpS with GP3-Q_2 intermediates and SPM6 TrpS with GP3-Q_2 intermediates), 4 isolated TrpB enzymes (LBCA TrpB at Ain and Q_2 intermediates, and ANC3 TrpB, SPM3 TrpB and SPM6 TrpB at Q_2 intermediate). In all cases starting from the preequilibrated structures through conventional MD simulations (see above). Initial Gaussian potentials of height 0.15 kcal mol^{-1}, deposited every 2 ps of MD simulation at 350 K, were gradually decreased on the basis of the well-tempered adaptive bias with a bias factor of 10. The well-tempered approach allows for a smooth convergence of the FEL reconstruction avoiding the risk of overfilling. Besides, the adaptive Gaussian width scheme, in which hills variance is adapted to local properties of the free-energy surface, was used. Second, we extracted ten snapshots from the initial metadynamics exploration mostly covering the conformational space sampled by each system. These ten snapshots were used as the starting structures for the multiple-walkers metadynamics simulations. This approach allows to increase the sampling of the conformational space and to reach convergence of individual-energy profiles. The ten replicas (walkers) are run in parallel and each walker reads the energy quantities (external potentials) deposited by the others during the simulation time. In this context, the ten walkers collaborate together to reconstruct the FEL. For this case each replica was run for 50-60 ns, giving a total of 500-600 ns per system (i.e. accumulated simulation time of *ca.* 2.3 µs).

Error Estimation. The error within each FEL was estimated by averaging ten free energy profiles along the first CV, progression within the path of conformations, at different time intervals. More precisely, ten FELs were computed using a 5 ns time interval from the last 50 ns of total simulation time (i. e. ten multiple-walkers aggregated simulation time) for each studied system. Errors were computed as the standard deviation within the ten extracted FELs (Figure **S12**).

Convergence. The convergence of the recovered FEL was assessed by monitoring the energy difference (ΔΔG) between selected regions of the conformational surface along the simulation time (see **Figure S13**). The selected

regions correspond to the open, partially closed and closed energy minimum found and also open and closed regions that exhibited higher in energy free energy values. For instance, in the LBCA TrpS system at Q_2 intermediate, the energy differences were computed between the partially closed local energy minimum found and the higher in energy closed and open regions (i.e. PC-C and PC-O energy differences).

Structural analysis. A set of structures from each local energy minimum were clustered to obtain representative metastable conformations (see **Figure S5**). For consistency with our previous work, the local energy minima and the associated representative structures are labeled as open (O), partially closed (PC) and closed (C) accordingly with the path of CV values (x axis); (O)=1-5, (PC)=5-10 and (C)=10-15. In addition, closed structures with large deviation (y axis values > 1.5) are labeled as (C*).

Caver analysis. The analysis of the available tunnels for the entrance of L-Serine was performed with the CAVER 3.0 software.[6] In this study we analyze the LBCA TrpS and LBCA TrpB systems at Ain intermediate. A total of 200 snapshots for the selected local energy minima from the metadynamic trajectories were subjected to Caver analysis. In particular, we used the structures from LBCA TrpS (Ain) open and the LBCA TrpB (Ain) partially closed energy minima. The starting point for the calculations was chosen at the L-Ser active site coordinates by alignment of the LBCA metastable structures at Ain intermediate with the X-ray structure (PDB ID 5DW0), which contains the Aex1 intermediate co-crystalized (intermediate formed after L-Serine covalent attachment with PLP). According to the parameters used in this study, a spherical probe of 0.9 Å radius was selected with a weighting coefficient of 1, and clustering threshold of 12.0.

Shortest Path Map analysis. The Shortest Path Map (SPM) analysis was performed using the metadynamics simulation of LBCA TrpB, and the MD simulation of the ANC3 variant. The first step for SPM construction relies on the calculation of the inter-residue mean distances and correlation values observed along the MD simulations and the conversion of this information into a simplified graph. For each residue of the enzyme a node is created. Each pair of nodes that displays a mean distance of less than 6 Å along the MD simulation time is connected through a line. The length of the connecting line between residues i and j is weighted according to their correlation value (dij=-log |Cij|). In this way, those pairs of residues exhibiting large correlation values (i.e. highly correlated, values closer to 1 or -1) will be connected through shorter lines, whereas long lines will be drawn for those presenting lower correlation values (i.e. non-correlated, values closer to 0). At this point, the generated graph is further simplified to identify the shortest path lengths. The algorithm goes through all nodes of the initial graph and detects the shortest paths to go from the first until the last protein residue. Following this strategy, those lines in the graph that are shorter, i.e. the connecting residues are more correlated, and that play a substantial role in the enzyme conformational dynamics are detected. The generated graph that we called SPM is then drawn on the 3D structure of the enzyme. More details about our SPM tool can be found in the recent publications [7] and [8].

Experimental methods:

Bacterial Strains and Chemicals. The proteins that were analyzed in this study were expressed in *E. coli* strain BL21 Gold (DE3) (purchased from Agilent Technologies). All chemicals used herein were purchased form commercial sources and were of analytical grade or higher.

Cloning. The genes for SPM3, SPM6, and SPM8 were codon optimized for expression in *E. coli* and purchased from Thermo Fisher Scientific (GeneArt Gene Synthesis). The genes were then cloned into a pET21a vector in a coupled digestion/ligation reaction using *Bsa*I and T4 DNA ligase,[9] which allowed for an isopropyl-β-thiogalactopyranoside (IPTG) inducible expression with a C-terminal His_6-tag.

Gene Expression and Protein Purification. The *E. coli* expression strain BL21 (DE3) Gold was transformed with plasmids harboring the genes for the TrpB variants SPM3, SPM6 and SPM8 and grown in 4 L lysogeny broth (LB) medium supplemented with 150 mg/mL ampicillin and 40 µM PLP at 37°C. When an OD_{600} of 0.6 was reached, expression was induced by addition of 0.5 mM IPTG and the cultures were further incubated over night at 20°C. Cells were then harvested by centrifugation and suspended in 50 mM KP (pH 7.5), 300 mM KCl, 10 mM imidazole, and 20 mM PLP. Cells were disrupted by sonication (Branson Sonifier W-250D, 60 % amplitude, 3 min, 2 s pulse, 2 s pause) and cell debris and insoluble aggregates were removed by centrifugation. The target proteins were purified from the supernatant by nickel-affinity chromatography (HisTrap™ FF crude or HisTrap excel, 5 mL, GE Healthcare) applying a linear imidazole gradient (10 mM to 500 mM). This was followed by size exclusion chromatography (Superdex 75 HiLoad 26/600, GE Healthcare) using 50 mM potassium phosphate (pH 7,5) and 300 mM KCl. The purified proteins were then dripped into liquid nitrogen and stored at -80°C.

The proteins LBCA TrpB, LBCA TrpA, ANC3 TrpB, and ANC3 TrpA were taken from previous work.[10]

Steady-State Enzyme Kinetics. In order to monitor TrpB activity, the difference in absorbance between indole and L-Trp was used ($\Delta\varepsilon_{290}$ = 1890 $M^{-1}cm^{-1}$). The reactions were performed at 30°C and changes in absorption were monitored with a spectrophotometer (JASCO V-750). The experimental conditions included 50 mM potassium phosphate (pH 7.5), 180 mM KCl, 40 µM PLP, saturating concentrations of one substrate (either L-Ser or indole) and varying concentrations of the second substrate. When a constant baseline absorption was reached, reactions were initiated by addition of TrpB or the TrpS complex. In the case of TrpS, TrpA was added in molar excess to ensure complete complex formation. Initial slopes were determined and divided by $\Delta\varepsilon_{290}$ to give the initial velocities (v_i). The values obtained for v_i were divided by the total concentration of TrpB enzyme ($[E]_t$) and plotted against the substrate concentration [S]. The Michaelis constant K_M and the turnover number k_{cat} were obtained by fitting to the Michaelis-Menten equation (1) using Origin 2019 (Origin Lab).

$$\frac{v_i}{[E]_t} = \frac{k_{cat}\,[S]}{K_M + [S]} \tag{1}$$

Supplementary Tables:

Table S1. Steady state enzyme kinetic parameters of LBCA TrpB in isolation and in the LBCA TrpS complex.

Kinetic parameters	LBCA TrpB	LBCA TrpS
k_{cat} [s^{-1}]	0.51 ± 0.04	0.059 ± 0.004
K_M^{Ind} [µM]	7.5 ± 1.6	9.1 ± 2.0
k_{cat}/K_M^{Ind} [M^{-1} s^{-1}]	$6.6 \cdot 10^4$	$6.3 \cdot 10^3$
k_{cat} [s^{-1}]	0.48 ± 0.01	0.057 ± 0.002
K_M^{Ser} [mM]	1.82 ± 0.13	0.28 ± 0.04
k_{cat}/K_M^{Ser} [M^{-1} s^{-1}]	272	209

Experimental conditions: 50 mM potassium phosphate pH 7.5, 180 mM KCl, 40 µM PLP, 30°C. The concentration of the substrate added in excess was at least five times its respective K_M. For the measurements of TrpB, 0.2 µM TrpB LBCA were used, while for the LBCA TrpS complex 2.0 µM LBCA TrpA and 1.0 µM LBCA TrpB were employed. Under these conditions TrpB was completely saturated with TrpA.

Table S2. Steady state enzyme kinetic parameters of ANC3 TrpB in isolation and in the ANC3 TrpS complex.

Kinetic parameters	ANC3 TrpB	ANC3 TrpS
k_{cat} [s^{-1}]	0.028 ± 0.001	0.91 ± 0.01
K_M^{Ind} [µM]	9.8 ± 1.2	27.7 ± 1.5
k_{cat}/K_M^{Ind} [M^{-1} s^{-1}]	$2.9 \cdot 10^3$	$3.1 \cdot 10^4$
k_{cat} [s^{-1}]	0.029 ± 0.001	0.81 ± 0.03
K_M^{Ser} [mM]	2.4 ± 0.5	0.62 ± 0.07
k_{cat}/K_M^{Ser} [M^{-1} s^{-1}]	12.1	$1.4 \cdot 10^3$

Experimental conditions: 50 mM potassium phosphate pH 7.5, 180 mM KCl, 40 µM PLP, 30°C. The concentration of the substrate added in excess was at least seven times its respective K_M. For the measurements of TrpB, 2.0 µM ANC3 TrpB were used, while for the ANC3 TrpS complex 5.0 µM ANC3 TrpA and 0.2 µM ANC3 TrpB were employed. Under these conditions TrpB was completely saturated with TrpA.

Table S3. Steady state enzyme kinetic parameters of SPM6 TrpB in isolation and in the SPM6 TrpS complex.

Kinetic constants	SPM6 TrpB	SPM6 TrpS
k_{cat} [s^{-1}]	0.23 ± 0.01	1.14 ± 0.07
K_M^{Ind} [µM]	19.8 ± 1.9	56.8 ± 9.6
k_{cat}/K_M^{Ind} [M^{-1} s^{-1}]	$1.2 \cdot 10^4$	$1.9 \cdot 10^4$
k_{cat} [s^{-1}]	0.17 ± 0.004	1.06 ± 0.04
K_M^{Ser} [mM]	1.9 ± 0.1	1.02 ± 0.16
k_{cat}/K_M^{Ser} [M^{-1} s^{-1}]	89	$1.1 \cdot 10^3$

Experimental conditions: 50 mM potassium phosphate pH 7.5, 180 mM KCl, 40 µM PLP, 30°C. The concentration of the substrate added in excess was at least five times its respective K_M. For the measurements of TrpB, 0.5 µM TrpB SPM6 were used, while for the SPM6 TrpS complex 0.4 µM ANC3 TrpA and 0.2 µM TrpB SPM6 were employed. Under these conditions TrpB was completely saturated with TrpA.

Table S4. Steady state enzyme kinetic parameters of SPM8 TrpB in isolation and in the SPM8 TrpS complex.

Kinetic parameters	SPM8 TrpB	SPM8 TrpS
k_{cat} [s^{-1}]	0.15 ± 0.004	0.32 ± 0.016
K_M^{Ind} [µM]	12.4 ± 1.1	19.0 ± 2.5
k_{cat}/K_M^{Ind} [M^{-1} s^{-1}]	$1.25 \cdot 10^4$	$1.55 \cdot 10^4$
k_{cat} [s^{-1}]	0.16 ± 0.005	0.27 ± 0.01
K_M^{Ser} [mM]	3.75 ± 0.3	0.51 ± 0.06
k_{cat}/K_M^{Ser} [M^{-1} s^{-1}]	41	578

Experimental conditions: 50 mM potassium phosphate pH 7.5, 180 mM KCl, 40 µM PLP, 30°C. The concentration of the substrate added in excess was at least six times its respective K_M. For the measurements of TrpB, 1.0 µM TrpB SPM8 were used, while for the SPM8 TrpS complex 0.75 µM ANC3 TrpA and 0.5 µM TrpB SPM8 were employed. Under these conditions TrpB was completely saturated with TrpA.

Table S5. Steady state enzyme kinetic parameters of SPM3 TrpB in isolation and in the SPM3 TrpS complex.

Kinetic parameters	TrpB SPM3	SPM3 TrpS
k_{cat} [s^{-1}]	0.033 ± 0.002	0.83 ± 0.04
K_M^{Ind} [µM]	16.3 ± 2.4	44.2 ± 5.3
k_{cat}/K_M^{Ind} [M^{-1} s^{-1}]	$2.0 \cdot 10^3$	$1.6 \cdot 10^4$
k_{cat} [s^{-1}]	0.032 ± 0.001	0.88 ± 0.01
K_M^{Ser} [mM]	0.38 ± 0.03	0.55 ± 0.03
k_{cat}/K_M^{Ser} [M^{-1} s^{-1}]	85	$1.5 \cdot 10^3$

Experimental conditions: 50 mM potassium phosphate pH 7.5, 180 mM KCl, 40 µM PLP, 30°C. The concentration of the substrate added in excess was at least five times its respective K_M For the measurements of TrpB, 1.5 µM TrpB SPM3 were used, while for the SPM3 TrpS complex 1.0 µM ANC3 TrpA and 0.5 µM TrpB SPM3 were employed. Under these conditions TrpB was completely saturated with TrpA.

Table S6. Conservation level of TrpB residues in extant organisms compared to positions identified by SPM and to activity enhancing positions identified in LBCA TrpB and *pf*TrpB. The SPM results for pfTrpB were taken from reference 2.

MSA position	amino acid																					Conservation > 90%	Position in LBCA TrpB	SPM of LBCA TrpB	Position in PfTrpB[11]	SPM of PfTrpB	combined SPM	SPM3 mutation	SPM6 mutation
	A	C	D	E	F	G	H	I	K	L	M	N	P	Q	R	S	T	V	W	Y	gap								
1	0,00	0,00	0,00	0,00	0,04	0,02	0,00	0,04	0,00	0,25	0,00	0,00	0,00	0,10	0,00	0,04	0,00	0,02	0,04	0,08	0,38	-	-	-	-	-	-	-	-
2	0,00	0,00	0,00	0,00	0,00	0,02	0,00	0,00	0,02	0,00	0,00	0,02	0,56	0,00	0,00	0,00	0,02	0,00	0,00	0,00	0,37	-	-	-	-	-	-	-	-
3	0,00	0,02	0,54	0,02	0,00	0,04	0,00	0,04	0,04	0,02	0,00	0,04	0,00	0,08	0,00	0,00	0,02	0,00	0,00	0,00	0,15	-	-	-	-	-	-	-	-
4	0,23	0,00	0,02	0,13	0,00	0,02	0,00	0,02	0,17	0,08	0,04	0,00	0,02	0,00	0,04	0,08	0,02	0,06	0,00	0,04	0,04	-	1	-	-	-	-	-	-
5	0,02	0,00	0,08	0,04	0,00	0,02	0,08	0,04	0,12	0,00	0,00	0,21	0,04	0,02	0,29	0,02	0,04	0,00	0,00	0,00	0,00	-	2	-	-	-	-	-	-
6	0,00	0,00	0,00	0,00	0,00	0,90	0,00	0,00	0,02	0,00	0,00	0,00	0,00	0,00	0,00	0,04	0,04	0,00	0,00	0,00	0,00	yes	3	-	1	-	-	-	-
7	0,00	0,00	0,00	0,00	0,00	0,00	0,17	0,00	0,19	0,00	0,02	0,00	0,00	0,00	0,56	0,00	0,00	0,00	0,00	0,06	0,00	-	4	-	2	-	-	-	-
8	0,00	0,00	0,00	0,00	0,71	0,00	0,00	0,00	0,00	0,02	0,00	0,00	0,00	0,00	0,00	0,00	0,00	0,00	0,00	0,27	0,00	-	5	-	3	-	-	-	-
9	0,02	0,00	0,00	0,00	0,00	**0,98**	0,00	0,00	0,00	0,00	0,00	0,00	0,00	0,00	0,00	0,00	0,00	0,00	0,00	0,00	0,00	yes	6	-	4	-	-	-	-
10	0,02	0,00	0,06	0,17	0,00	0,00	0,00	0,08	0,27	0,00	0,00	0,00	0,17	0,06	0,12	0,00	0,04	0,02	0,00	0,00	0,00	-	7	-	5	-	-	-	-
11	0,00	0,00	0,00	0,00	0,35	0,00	0,04	0,00	0,00	0,00	0,00	0,00	0,00	0,00	0,00	0,00	0,00	0,00	0,00	0,62	0,00	-	8	-	6	-	-	-	-
12	0,00	0,00	0,00	0,00	0,00	**1,00**	0,00	0,00	0,00	0,00	0,00	0,00	0,00	0,00	0,00	0,00	0,00	0,00	0,00	0,00	0,00	yes	9	-	7	-	-	-	-
13	0,00	0,00	0,00	0,00	0,00	**1,00**	0,00	0,00	0,00	0,00	0,00	0,00	0,00	0,00	0,00	0,00	0,00	0,00	0,00	0,00	0,00	yes	10	-	8	-	-	-	-
14	0,00	0,02	0,00	0,00	0,00	0,00	0,00	0,10	0,06	0,00	0,06	0,00	0,00	0,33	0,37	0,02	0,04	0,02	0,00	0,00	0,00	-	11	-	9	yes	-	-	-
15	0,00	0,00	0,00	0,00	0,37	0,00	0,00	0,00	0,00	0,00	0,00	0,00	0,00	0,00	0,00	0,00	0,00	0,00	0,00	0,63	0,00	-	12	yes	10	yes	yes	-	-
16	0,08	0,00	0,00	0,00	0,00	0,00	0,00	0,00	0,00	0,00	0,00	0,00	0,00	0,00	0,00	0,00	0,00	0,92	0,00	0,00	0,00	yes	13	yes	11	yes	yes	-	-
17	0,12	0,00	0,00	0,00	0,00	0,00	0,00	0,00	0,00	0,00	0,00	0,00	0,77	0,00	0,00	0,12	0,00	0,00	0,00	0,00	0,00	-	14	yes	12	yes	yes	-	-
18	0,00	0,00	0,00	**0,96**	0,00	0,00	0,00	0,00	0,00	0,00	0,00	0,00	0,00	0,00	0,00	0,00	0,00	0,04	0,00	0,00	0,00	yes	15	-	13	-	-	-	-
19	0,00	0,00	0,00	0,00	0,00	0,00	0,00	0,08	0,00	0,10	0,00	0,00	0,00	0,00	0,00	0,00	0,75	0,08	0,00	0,00	0,00	-	16	-	14	yes	-	-	-
20	0,00	0,00	0,00	0,00	0,00	0,00	0,00	0,06	0,00	0,90	0,02	0,00	0,00	0,00	0,00	0,00	0,00	0,02	0,00	0,00	0,00	yes	17	yes	15	yes	yes	-	-
21	0,00	0,00	0,00	0,00	0,00	0,00	0,00	0,29	0,00	0,04	0,62	0,00	0,00	0,00	0,00	0,00	0,00	0,06	0,00	0,00	0,00	-	18	-	16	-	-	-	-
22	0,12	0,00	0,00	0,02	0,00	0,02	0,00	0,00	0,00	0,00	0,00	0,12	0,62	0,00	0,00	0,08	0,04	0,00	0,00	0,00	0,00	-	19	-	17	yes	-	-	-
23	0,85	0,00	0,00	0,00	0,00	0,00	0,00	0,00	0,00	0,12	0,00	0,00	0,00	0,00	0,00	0,00	0,00	0,04	0,00	0,00	0,00	-	20	yes	18	-	-	-	-
24	0,00	0,00	0,00	0,00	0,00	0,00	0,00	0,21	0,00	0,69	0,00	0,00	0,00	0,00	0,00	0,00	0,00	0,10	0,00	0,00	0,00	-	21	-	19	-	-	-	-

54	53	52	51	50	49	48	47	46	45	44	43	42	41	40	39	38	37	36	35	34	33	32	31	30	29	28	27	26	25
0,46	0,00	0,00	0,13	0,00	0,00	0,08	0,15	0,00	0,04	0,37	0,04	0,00	0,27	0,04	0,00	0,12	0,00	0,48	0,00	0,00	0,15	0,06	0,00	0,62	0,19	0,02	0,00	0,00	0,06
0,00	0,00	0,00	0,00	0,00	0,00	0,00	0,00	0,00	0,00	0,00	0,00	0,00	0,00	0,00	0,00	0,00	0,00	0,00	0,00	0,00	0,00	0,00	0,00	0,00	0,02	0,02	0,00	0,00	0,00
0,00	0,00	0,29	0,00	0,00	0,06	0,08	0,27	0,00	0,08	0,04	0,00	0,00	0,08	0,00	0,92	0,13	0,00	0,00	0,00	0,00	0,04	0,06	0,00	0,00	0,02	0,08	0,00	0,13	0,17
0,00	0,00	0,37	0,02	0,00	0,04	0,00	0,13	0,00	0,77	0,17	0,02	0,00	0,35	0,10	0,04	0,10	0,00	0,00	0,00	0,00	0,08	0,38	0,00	0,17	0,38	0,62	0,00	0,81	0,35
0,00	0,33	0,00	0,00	0,02	0,02	0,08	0,02	0,19	0,00	0,00	0,00	**0,96**	0,00	0,00	0,00	0,00	0,02	0,08	0,00	0,04	0,00	0,00	0,17	0,00	0,00	0,00	0,00	0,00	0,00
0,00	0,00	0,00	0,02	0,00	0,00	0,06	0,02	0,00	0,00	0,02	0,00	0,00	0,04	0,04	0,02	0,02	0,02	0,00	0,00	0,00	0,00	0,00	0,00	0,10	0,00	0,02	0,00	0,00	0,02
0,00	0,00	0,23	0,04	0,00	0,04	0,10	0,06	0,00	0,00	0,00	0,00	0,00	0,00	0,00	0,00	0,04	0,00	0,00	0,00	0,00	0,08	0,02	0,00	0,00	0,00	0,00	0,00	0,00	0,02
0,00	0,00	0,00	0,00	0,00	0,00	0,00	0,00	0,00	0,00	0,00	0,06	0,00	0,00	0,00	0,00	0,00	0,00	0,08	0,00	0,00	0,00	0,02	0,00	0,00	0,00	0,02	0,00	0,00	0,06
0,00	0,00	0,00	0,25	0,00	0,00	0,08	0,13	0,00	0,00	0,15	0,04	0,00	0,04	0,06	0,00	0,12	0,58	0,00	0,00	0,00	0,23	0,02	0,00	0,00	0,10	0,00	0,00	0,00	0,02
0,00	0,00	0,00	0,00	0,73	0,33	0,00	0,00	0,65	0,02	0,00	0,29	0,00	0,00	0,00	0,00	0,00	0,02	0,12	0,00	0,00	0,00	0,10	0,00	0,02	0,02	0,00	**0,96**	0,00	0,17
0,00	0,00	0,00	0,00	0,06	0,02	0,00	0,00	0,02	0,00	0,00	0,08	0,00	0,00	0,00	0,00	0,00	0,02	0,00	0,00	0,00	0,00	0,00	0,00	0,02	0,00	0,00	0,00	0,00	0,02
0,00	0,00	0,00	0,04	0,02	0,00	0,00	0,02	0,00	0,00	0,04	0,04	0,00	0,06	0,00	0,02	0,21	0,04	0,00	0,00	0,00	0,00	0,08	0,00	0,00	0,00	0,08	0,00	0,00	0,08
0,00	0,00	0,00	0,00	0,00	0,00	0,00	0,00	0,00	0,00	0,00	0,00	0,00	0,00	0,71	0,00	0,02	0,00	0,00	0,00	0,00	0,00	0,00	0,00	0,00	0,00	0,00	0,00	0,00	0,00
0,00	0,00	0,02	0,04	0,04	0,00	0,08	0,02	0,00	0,04	0,06	0,33	0,00	0,04	0,00	0,00	0,08	0,00	0,00	0,00	0,00	0,06	0,02	0,00	0,02	0,12	0,02	0,00	0,04	0,02
0,00	0,00	0,02	0,31	0,02	0,00	0,12	0,10	0,02	0,06	0,06	0,06	0,00	0,04	0,04	0,00	0,13	0,23	0,00	0,00	0,00	0,27	0,12	0,00	0,00	0,10	0,02	0,00	0,00	0,00
0,04	0,00	0,00	0,02	0,04	0,00	0,08	0,06	0,00	0,00	0,06	0,00	0,02	0,06	0,02	0,00	0,00	0,02	0,00	0,00	0,00	0,00	0,00	0,00	0,00	0,02	0,00	0,00	0,02	0,00
0,00	0,00	0,08	0,13	0,00	0,00	0,02	0,00	0,00	0,00	0,04	0,00	0,00	0,02	0,00	0,00	0,04	0,02	0,02	0,00	0,00	0,06	0,06	0,00	0,02	0,00	0,12	0,00	0,00	0,00
0,50	0,00	0,00	0,00	0,00	0,00	0,00	0,00	0,00	0,00	0,00	0,04	0,00	0,02	0,00	0,00	0,00	0,02	0,00	0,00	0,00	0,02	0,02	0,00	0,04	0,04	0,00	0,04	0,00	0,00
0,00	0,00	0,00	0,00	0,02	0,02	0,02	0,00	0,00	0,00	0,00	0,02	0,00	0,00	0,00	0,00	0,00	0,02	0,00	0,04	0,00	0,00	0,00	0,00	0,00	0,00	0,00	0,00	0,00	0,00
0,00	0,67	0,00	0,00	0,06	0,48	0,23	0,02	0,12	0,00	0,00	0,00	0,02	0,00	0,00	0,00	0,00	0,00	0,23	0,00	0,00	0,02	0,06	0,83	0,00	0,00	0,00	0,00	0,00	0,02
0,00	0,00	0,00	0,00	0,00	0,00	0,00	0,00	0,00	0,00	0,00	0,00	0,00	0,00	0,00	0,00	0,00	0,00	0,00	**0,96**	**0,96**	0,00	0,00	0,00	0,00	0,00	0,00	0,00	0,00	0,00
-	-	-	-	-	-	-	-	-	-	-	-	yes	-	-	yes	-	-	-	-	-	-	-	-	-	-	-	yes	-	-
49	48	47	46	45	44	43	42	41	40	39	38	37	36	35	34	33	32	31	-	-	30	29	28	27	26	25	24	23	22
yes	-	-	-	yes	-	-	yes	-	-	yes	-	-	yes	-	yes	-	-	-	-	-	-	-	-	-	-	-	-	-	-
47	46	45	44	43	42	41	40	39	38	37	36	35	34	33	32	31	30	29	-	-	28	27	26	25	24	23	22	21	20
-	-	-	-	yes	-	-	yes	-	-	yes	-	-	-	-	yes	-	-	yes	-	-	-	-	yes	-	-	yes	-	-	yes
-	-	-	-	yes	-	-	yes	-	-	yes	-	-	-	-	yes	-	-	-	-	-	-	-	-	-	-	-	-	-	-
-	-	-	-	-	-	-	-	-	-	-	-	-	-	-	-	-	-	-	-	-	-	-	-	-	-	-	-	-	-
-	-	-	-	-	-	-	yes	-	-	-	-	-	yes	-	-	-	-	-	-	-	-	-	-	-	-	-	-	-	-

84	83	82	81	80	79	78	77	76	75	74	73	72	71	70	69	68	67	66	65	64	63	62	61	60	59	58	57	56	55
0,00	0,12	0,00	0,00	0,00	0,00	0,00	0,00	0,00	0,00	0,00	0,65	0,00	0,00	0,08	0,02	0,06	0,00	0,02	0,00	0,06	0,88	0,02	0,00	0,00	0,04	0,00	0,00	0,00	0,00
0,00	0,02	0,00	0,00	0,00	0,00	0,00	0,00	0,00	0,00	0,00	0,23	0,00	0,00	0,02	0,00	0,00	0,00	0,00	0,00	0,00	0,12	0,00	0,00	0,00	0,00	0,00	0,00	0,00	0,00
0,00	0,00	0,00	0,92	0,06	0,00	0,00	0,00	0,00	0,00	0,00	0,00	0,00	0,02	0,00	0,15	0,04	0,00	0,00	0,00	0,00	0,00	0,00	0,00	0,00	0,00	0,00	0,00	0,00	0,00
0,00	0,00	0,00	0,08	0,94	0,00	0,00	0,00	0,00	0,00	0,00	0,00	0,00	0,00	0,00	0,13	0,42	0,00	0,00	0,00	0,46	0,00	0,02	0,00	0,00	0,00	0,00	0,21	0,00	0,00
0,04	0,00	0,02	0,00	0,00	0,00	0,00	0,10	0,06	0,00	0,00	0,02	0,00	0,00	0,10	0,00	0,00	0,00	0,00	0,00	0,00	0,00	0,48	0,00	0,00	0,00	0,00	0,00	0,00	0,00
0,00	0,00	0,00	0,00	0,00	0,00	0,00	0,00	0,00	0,00	0,00	0,00	**1,00**	0,63	0,00	0,00	0,00	0,00	0,00	0,00	0,02	0,00	0,00	0,00	0,00	0,10	0,00	0,00	0,00	0,90
**0,96**	0,00	0,00	0,00	0,00	0,00	0,00	0,00	0,00	0,00	0,00	0,00	0,00	0,00	0,00	0,23	0,02	0,00	0,00	0,02	0,00	0,00	0,10	0,00	0,00	0,00	0,00	0,00	0,00	0,00
0,00	0,00	0,00	0,00	0,00	0,00	0,00	0,00	0,00	0,73	0,00	0,00	0,00	0,00	0,06	0,00	0,00	0,00	0,02	0,00	0,00	0,00	0,00	0,00	0,04	0,00	0,00	0,00	0,00	0,00
0,00	0,00	0,00	0,00	0,00	0,00	**1,00**	0,00	0,00	0,00	0,65	0,00	0,00	0,00	0,00	0,25	0,23	0,00	0,00	0,04	0,10	0,00	0,00	0,00	0,00	0,00	0,00	0,00	0,02	0,00
0,00	0,19	0,87	0,00	0,00	0,00	0,00	0,90	0,02	0,00	0,00	0,00	0,00	0,00	0,50	0,04	0,00	0,00	0,75	0,00	0,00	0,00	0,12	0,00	0,94	0,06	0,00	0,00	0,00	0,00
0,00	0,02	0,08	0,00	0,00	0,00	0,00	0,00	0,00	0,00	0,00	0,00	0,00	0,00	0,00	0,00	0,00	0,00	0,12	0,00	0,00	0,00	0,00	0,00	0,02	0,00	0,00	0,00	0,00	0,00
0,00	0,50	0,00	0,00	0,00	0,00	0,00	0,00	0,00	0,00	0,00	0,00	0,00	0,00	0,00	0,00	0,02	0,00	0,00	0,46	0,02	0,00	0,00	0,00	0,00	0,02	0,06	0,00	0,00	0,08
0,00	0,00	0,00	0,00	0,00	0,00	0,00	0,00	0,00	0,00	0,00	0,00	0,00	0,00	0,00	0,00	0,00	0,00	0,00	0,00	0,08	0,00	0,00	0,00	0,00	0,71	0,00	0,77	0,00	0,00
0,00	0,00	0,04	0,00	0,00	0,00	0,00	0,00	0,00	0,00	0,08	0,00	0,00	0,00	0,00	0,04	0,02	0,00	0,00	0,00	0,04	0,00	0,00	0,00	0,00	0,02	0,00	0,02	0,00	0,00
0,00	0,00	0,00	0,00	0,00	**1,00**	0,00	0,00	0,00	0,00	0,27	0,00	0,00	0,00	0,00	0,02	0,19	0,00	0,00	0,44	0,23	0,00	0,00	0,00	0,00	0,00	0,00	0,00	**0,98**	0,00
0,00	0,00	0,00	0,00	0,00	0,00	0,00	0,00	0,00	0,00	0,00	0,02	0,00	0,00	0,00	0,00	0,00	0,44	0,00	0,04	0,00	0,00	0,00	0,00	0,00	0,02	0,42	0,00	0,00	0,02
0,00	0,00	0,00	0,00	0,00	0,00	0,00	0,00	0,00	0,00	0,00	0,08	0,00	0,00	0,00	0,06	0,00	0,56	0,00	0,00	0,00	0,00	0,00	0,12	0,00	0,04	0,52	0,00	0,00	0,00
0,00	0,15	0,00	0,00	0,00	0,00	0,00	0,00	0,00	0,27	0,00	0,00	0,00	0,00	0,06	0,00	0,00	0,00	0,00	0,00	0,00	0,00	0,00	0,00	0,00	0,00	0,00	0,00	0,00	0,00
0,00	0,00	0,00	0,00	0,00	0,00	0,00	0,00	0,00	0,00	0,00	0,00	0,00	0,00	0,08	0,00	0,00	0,00	0,10	0,00	0,00	0,00	0,00	0,00	0,00	0,00	0,00	0,00	0,00	0,00
0,00	0,00	0,00	0,00	0,00	0,00	0,00	0,00	0,92	0,00	0,00	0,00	0,00	0,00	0,12	0,06	0,00	0,00	0,00	0,00	0,00	0,00	0,27	0,88	0,00	0,00	0,00	0,00	0,00	0,00
0,00	0,00	0,00	0,00	0,00	0,00	0,00	0,00	0,00	0,00	0,00	0,00	0,00	0,35	0,00	0,00	0,00	0,00	0,00	0,00	0,00	0,00	0,00	0,00	0,00	0,00	0,00	0,00	0,00	0,00
yes	-	-	yes	yes	yes	yes	yes	yes	-	-	-	yes	-	-	-	-	-	-	-	-	-	-	-	yes	-	-	-	yes	yes
79	78	77	76	75	74	73	72	71	70	69	68	67	66	65	64	63	62	61	60	59	58	57	56	55	54	53	52	51	50
-	yes	-	-	yes	yes	-	yes	yes	yes	yes	-	-	-	-	-	-	-	-	-	-	-	-	-	-	-	yes	yes	yes	-
77	76	75	74	73	72	71	70	69	68	67	66	65	64	63	62	61	60	59	58	57	56	55	54	53	52	51	50	49	48
-	-	-	-	yes	yes	yes	yes	yes	-	-	-	-	-	-	-	-	-	-	-	-	-	-	-	-	-	yes	yes	yes	-
-	-	-	-	yes	yes	-	yes	yes	-	-	-	-	-	-	-	-	-	-	-	-	-	-	-	-	-	yes	yes	yes	-
-	-	-	-	-	-	-	-	-	-	-	-	-	-	-	-	-	-	-	-	-	-	-	-	-	-	yes	-	-	-
-	-	-	-	-	-	-	-	-	-	-	-	-	-	-	-	-	-	-	-	-	-	-	-	-	-	yes	-	-	-

114	113	112	111	110	109	108	107	106	105	104	103	102	101	100	99	98	97	96	95	94	93	92	91	90	89	88	87	86	85
**1,00**	0,00	0,00	0,00	**1,00**	0,00	0,00	0,00	0,00	0,00	0,00	0,00	0,02	0,00	**0,96**	0,00	0,00	0,58	0,00	0,04	0,00	0,38	0,00	0,00	0,00	0,00	0,00	0,90	0,00	0,00
0,00	0,00	0,00	0,00	0,00	0,00	0,00	0,00	0,00	0,00	0,00	0,00	0,00	0,00	0,04	0,00	0,00	0,04	0,00	0,00	0,00	0,17	0,00	0,00	0,00	0,00	0,00	0,00	0,00	0,00
0,00	0,00	0,00	0,00	0,00	0,00	0,00	0,00	0,00	0,00	0,00	0,00	0,00	0,00	0,00	0,00	0,00	0,00	0,00	0,00	0,00	0,00	0,00	0,00	0,00	0,00	0,00	0,00	0,00	0,00
0,00	0,00	0,00	**1,00**	0,00	0,00	0,00	0,02	0,00	0,00	0,00	0,00	0,00	0,02	0,00	0,00	0,00	0,00	0,00	0,00	0,00	0,00	0,00	0,00	0,00	0,00	0,00	0,00	0,00	0,00
0,00	0,00	0,00	0,00	0,00	0,00	0,00	0,00	0,00	0,00	0,00	0,00	0,06	0,00	0,00	0,00	0,00	0,00	0,00	0,00	0,00	0,00	0,00	0,00	0,00	0,00	0,00	0,00	0,00	0,00
0,00	**1,00**	0,00	0,00	0,00	0,00	0,00	0,00	0,00	0,00	**0,96**	0,00	0,00	0,02	0,00	0,00	0,00	0,17	0,00	**0,96**	0,00	0,00	0,00	0,00	0,00	0,00	0,00	0,00	**1,00**	0,19
0,00	0,00	0,00	0,00	0,00	0,00	0,00	0,00	0,00	0,00	0,00	0,00	0,04	0,00	0,00	0,00	0,00	0,00	0,00	0,00	0,00	0,00	0,00	0,00	0,00	0,00	**1,00**	0,00	0,00	0,00
0,00	0,00	0,00	0,00	0,00	**0,98**	0,33	0,00	0,00	0,00	0,00	0,00	0,00	0,00	0,00	0,02	0,00	0,12	0,00	0,00	0,35	0,00	0,00	0,00	0,67	0,00	0,00	0,00	0,00	0,00
0,00	0,00	0,00	0,00	0,00	0,00	0,00	0,06	0,42	**1,00**	0,02	0,00	0,06	0,65	0,00	0,00	0,00	0,00	0,00	0,00	0,00	0,00	0,00	0,00	0,00	**1,00**	0,00	0,00	0,00	0,00
0,00	0,00	0,00	0,00	0,00	0,00	0,31	0,00	0,00	0,00	0,00	0,02	0,00	0,04	0,00	**0,98**	**1,00**	0,00	0,00	0,00	0,54	0,00	0,00	0,00	0,19	0,00	0,00	0,00	0,00	0,04
0,00	0,00	0,00	0,00	0,00	0,00	0,00	0,00	0,00	0,00	0,00	0,92	0,00	0,02	0,00	0,00	0,00	0,00	0,00	0,00	0,04	0,00	0,00	0,00	0,02	0,00	0,00	0,00	0,00	0,00
0,00	0,00	0,00	0,00	0,00	0,00	0,00	0,00	0,00	0,00	0,00	0,00	0,00	0,00	0,00	0,00	0,00	0,00	0,00	0,00	0,00	0,00	**1,00**	**1,00**	0,00	0,00	0,00	0,00	0,00	0,00
0,00	0,00	0,00	0,00	0,00	0,00	0,00	0,00	0,10	0,00	0,00	0,00	0,00	0,00	0,00	0,00	0,00	0,00	0,00	0,00	0,00	0,00	0,00	0,00	0,00	0,00	0,00	0,00	0,00	0,00
0,00	0,00	0,00	0,00	0,00	0,00	0,00	0,00	0,04	0,00	0,00	0,00	0,00	0,10	0,00	0,00	0,00	0,00	**1,00**	0,00	0,00	0,00	0,00	0,00	0,00	0,00	0,00	0,00	0,00	0,00
0,00	0,00	0,00	0,00	0,00	0,00	0,00	0,92	0,12	0,00	0,02	0,00	0,56	0,15	0,00	0,00	0,00	0,00	0,00	0,00	0,00	0,00	0,00	0,00	0,00	0,00	0,00	0,00	0,00	0,00
0,00	0,00	0,00	0,00	0,00	0,00	0,00	0,00	0,04	0,00	0,00	0,02	0,02	0,00	0,00	0,00	0,00	0,00	0,00	0,00	0,00	0,00	0,00	0,00	0,00	0,00	0,00	0,10	0,00	0,02
0,00	0,00	**1,00**	0,00	0,00	0,00	0,00	0,00	0,29	0,00	0,00	0,04	0,00	0,00	0,00	0,00	0,00	0,00	0,00	0,00	0,00	0,42	0,00	0,00	0,00	0,00	0,00	0,00	0,00	0,75
0,00	0,00	0,00	0,00	0,00	0,02	0,37	0,00	0,00	0,00	0,00	0,00	0,00	0,00	0,00	0,00	0,00	0,10	0,00	0,00	0,08	0,02	0,00	0,00	0,12	0,00	0,00	0,00	0,00	0,00
0,00	0,00	0,00	0,00	0,00	0,00	0,00	0,00	0,00	0,00	0,00	0,00	0,00	0,00	0,00	0,00	0,00	0,00	0,00	0,00	0,00	0,00	0,00	0,00	0,00	0,00	0,00	0,00	0,00	0,00
0,00	0,00	0,00	0,00	0,00	0,00	0,00	0,00	0,00	0,00	0,00	0,00	0,25	0,00	0,00	0,00	0,00	0,00	0,00	0,00	0,00	0,00	0,00	0,00	0,00	0,00	0,00	0,00	0,00	0,00
0,00	0,00	0,00	0,00	0,00	0,00	0,00	0,00	0,00	0,00	0,00	0,00	0,00	0,00	0,00	0,00	0,00	0,00	0,00	0,00	0,00	0,00	0,00	0,00	0,00	0,00	0,00	0,00	0,00	0,00
yes	yes	yes	yes	yes	yes	-	yes	-	yes	yes	yes	-	-	yes	yes	yes	-	yes	yes	-	-	yes	yes	-	yes	yes	yes	yes	-
109	108	107	106	105	104	103	102	101	100	99	98	97	96	95	94	93	92	91	90	89	88	87	86	85	84	83	82	81	80
yes	yes	yes	-	-	-	-	-	-	-	-	-	-	-	-	-	-	-	-	-	-	-	yes	-	yes	yes	yes	yes	-	-
107	106	105	104	103	102	101	100	99	98	97	96	95	94	93	92	91	90	89	88	87	86	85	84	83	82	81	80	79	78
yes	yes	yes	-	-	-	-	-	-	-	-	-	-	-	-	-	-	-	-	-	-	-	yes	yes	yes	yes	yes	-	-	-
yes	yes	yes	-	-	-	-	-	-	-	-	-	-	-	-	-	-	-	-	-	-	-	yes	-	yes	yes	yes	-	-	-
-	-	-	-	-	-	-	-	-	-	-	-	-	-	-	-	-	-	-	-	-	-	-	-	-	-	-	-	-	-
-	-	-	-	-	-	-	-	-	-	-	-	-	-	-	-	-	-	-	-	-	-	-	-	-	-	-	-	-	-

144	143	142	141	140	139	138	137	136	135	134	133	132	131	130	129	128	127	126	125	124	123	122	121	120	119	118	117	116	115
0,00	0,00	0,04	0,02	0,00	0,00	0,40	0,00	0,00	0,00	0,00	0,00	0,04	0,00	0,00	0,00	0,04	0,06	0,96	0,44	0,33	0,12	1,00	0,00	0,92	0,00	0,00	0,00	0,00	0,00
0,00	0,00	0,00	0,02	0,00	0,00	0,00	0,00	0,00	0,00	0,00	0,00	0,67	0,00	0,00	0,00	0,00	0,00	0,00	0,25	0,00	0,00	0,00	0,00	0,00	0,00	0,00	0,00	0,00	0,00
0,00	0,00	0,00	0,00	1,00	0,04	0,00	0,00	0,00	0,00	0,00	0,08	0,00	0,10	0,00	0,02	0,00	0,00	0,00	0,00	0,00	0,00	0,00	0,00	0,00	0,00	0,00	0,00	0,00	0,00
0,00	0,00	0,46	0,00	0,00	0,44	0,33	0,00	0,00	0,00	0,00	0,19	0,00	0,56	0,00	0,00	0,00	0,00	0,00	0,00	0,00	0,00	0,00	0,00	0,00	0,00	0,00	0,00	0,00	0,00
0,00	0,00	0,00	0,00	0,00	0,00	0,00	0,00	0,00	0,23	0,00	0,00	0,00	0,00	0,12	0,00	0,35	0,00	0,00	0,00	0,00	0,00	0,00	0,00	0,00	0,00	0,00	0,00	0,00	0,00
0,00	0,00	0,00	0,00	0,00	0,00	0,00	1,00	0,00	0,00	0,00	0,00	0,00	0,00	0,00	0,94	0,00	0,00	0,00	0,31	0,00	0,00	0,00	0,00	0,00	0,00	1,00	0,00	0,00	1,00
0,00	0,00	0,00	0,00	0,00	0,00	0,00	0,00	0,00	0,00	0,00	0,04	0,00	0,00	0,00	0,00	0,00	0,00	0,00	0,00	0,00	0,00	0,00	0,00	0,00	0,00	0,00	1,00	0,00	0,00
0,00	0,00	0,02	0,27	0,00	0,00	0,00	0,00	0,00	0,00	0,19	0,10	0,00	0,00	0,00	0,00	0,00	0,00	0,00	0,00	0,12	0,10	0,00	0,00	0,00	0,12	0,00	0,00	0,00	0,00
0,00	0,00	0,17	0,00	0,00	0,17	0,06	0,00	0,00	0,00	0,00	0,02	0,00	0,10	0,00	0,02	0,00	0,06	0,00	0,00	0,00	0,00	0,00	0,00	0,00	0,00	0,00	0,00	0,00	0,00
0,00	0,00	0,00	0,00	0,00	0,06	0,00	0,00	0,00	0,00	0,00	0,00	0,00	0,00	0,69	0,00	0,52	0,31	0,00	0,00	0,00	0,00	0,00	0,00	0,00	0,00	0,00	0,00	0,00	0,00
0,00	0,00	0,00	0,12	0,00	0,00	0,00	0,00	0,96	0,00	0,00	0,00	0,00	0,00	0,19	0,00	0,06	0,13	0,00	0,00	0,00	0,12	0,00	0,00	0,00	0,00	0,00	0,00	0,00	0,00
0,00	0,00	0,00	0,00	0,00	0,00	0,00	0,00	0,00	0,00	0,00	0,00	0,00	0,00	0,00	0,02	0,00	0,10	0,00	0,00	0,00	0,00	0,00	0,00	0,00	0,00	0,00	0,00	0,00	0,00
0,00	0,00	0,00	0,00	0,00	0,00	0,00	0,00	0,00	0,00	0,00	0,00	0,00	0,15	0,00	0,00	0,00	0,00	0,00	0,00	0,00	0,00	0,00	0,00	0,00	0,00	0,00	0,00	0,00	0,00
1,00	0,00	0,10	0,00	0,00	0,00	0,04	0,00	0,04	0,00	0,00	0,04	0,00	0,06	0,00	0,00	0,00	0,00	0,00	0,00	0,00	0,00	0,00	0,00	0,00	0,00	0,00	0,00	1,00	0,00
0,00	1,00	0,21	0,00	0,00	0,00	0,02	0,00	0,00	0,00	0,00	0,00	0,00	0,00	0,00	0,00	0,00	0,27	0,00	0,00	0,00	0,00	0,00	0,00	0,00	0,00	0,00	0,00	0,00	0,00
0,00	0,00	0,00	0,00	0,00	0,00	0,10	0,00	0,00	0,00	0,00	0,00	0,00	0,04	0,00	0,00	0,00	0,00	0,00	0,00	0,00	0,00	0,00	0,15	0,08	0,00	0,00	0,00	0,00	0,00
0,00	0,00	0,00	0,12	0,00	0,10	0,06	0,00	0,00	0,00	0,00	0,08	0,10	0,00	0,00	0,00	0,00	0,02	0,04	0,00	0,00	0,62	0,00	0,77	0,00	0,10	0,00	0,00	0,00	0,00
0,00	0,00	0,00	0,46	0,00	0,19	0,00	0,00	0,00	0,00	0,81	0,46	0,19	0,00	0,00	0,00	0,00	0,06	0,00	0,00	0,56	0,06	0,00	0,08	0,00	0,79	0,00	0,00	0,00	0,00
0,00	0,00	0,00	0,00	0,00	0,00	0,00	0,00	0,00	0,00	0,00	0,00	0,00	0,00	0,00	0,00	0,00	0,00	0,00	0,00	0,00	0,00	0,00	0,00	0,00	0,00	0,00	0,00	0,00	0,00
0,00	0,00	0,00	0,00	0,00	0,00	0,00	0,00	0,00	0,77	0,00	0,00	0,00	0,00	0,00	0,00	0,04	0,00	0,00	0,00	0,00	0,00	0,00	0,00	0,00	0,00	0,00	0,00	0,00	0,00
0,00	0,00	0,00	0,00	0,00	0,00	0,00	0,00	0,00	0,00	0,00	0,00	0,00	0,00	0,00	0,00	0,00	0,00	0,00	0,00	0,00	0,00	0,00	0,00	0,00	0,00	0,00	0,00	0,00	0,00
yes	yes	-	-	yes	-	-	yes	yes	-	-	-	-	-	-	yes	-	-	yes	-	-	-	yes	-	yes	-	yes	yes	yes	yes
139	138	137	136	135	134	133	132	131	130	129	128	127	126	125	124	123	122	121	120	119	118	117	116	115	114	113	112	111	110
-	-	-	-	-	-	-	-	-	-	-	-	-	-	-	-	-	-	-	-	-	-	-	-	-	-	-	yes	yes	yes
137	136	135	134	133	132	131	130	129	128	127	126	125	124	123	122	121	120	119	118	117	116	115	114	113	112	111	110	109	108
-	-	-	-	-	-	-	-	-	-	-	-	-	-	-	-	-	-	-	-	-	-	-	-	yes	-	-	yes	-	-
-	-	-	-	-	-	-	-	-	-	-	-	-	-	-	-	-	-	-	-	-	-	-	-	-	-	-	yes	-	-
-	-	-	-	-	-	-	-	-	-	-	-	-	-	-	-	-	-	-	-	-	-	-	-	-	-	-	-	-	-
-	-	-	-	-	-	-	-	-	-	-	-	-	-	-	-	-	-	-	-	-	-	-	-	-	-	-	-	-	-

174	173	172	171	170	169	168	167	166	165	164	163	162	161	160	159	158	157	156	155	154	153	152	151	150	149	148	147	146	145
0,00	0,00	0,00	**0,98**	0,00	0,00	0,00	0,00	0,13	0,02	0,00	0,12	0,04	0,02	0,06	0,00	0,00	0,00	0,81	0,00	0,00	0,00	0,00	0,00	0,00	0,06	0,08	0,00	0,00	0,40
0,00	0,00	0,00	0,00	0,00	0,00	0,00	0,00	0,00	0,00	0,00	0,04	0,00	0,00	0,00	0,00	0,00	0,00	0,00	0,00	0,02	0,00	0,00	0,00	0,00	0,00	0,00	0,00	0,00	0,00
0,02	0,00	0,00	0,00	**0,96**	0,00	0,00	0,00	0,00	0,00	0,00	0,00	0,12	0,00	0,00	0,00	0,00	0,02	0,00	0,00	0,00	0,00	0,00	0,00	0,00	0,00	0,00	0,00	0,00	0,00
**0,96**	0,00	0,00	0,00	0,04	0,00	0,00	0,00	0,00	0,00	0,00	0,00	0,21	0,00	0,08	0,00	0,00	0,58	0,00	0,00	0,00	0,00	0,35	0,00	0,00	0,00	0,00	0,00	0,00	0,02
0,00	0,00	0,00	0,00	0,00	0,00	0,00	0,00	0,00	0,00	0,00	0,00	0,02	0,00	0,00	0,00	0,00	0,00	0,00	0,00	0,00	0,04	0,00	0,00	0,00	0,63	0,00	0,00	0,00	0,00
0,00	0,00	0,00	0,00	0,00	0,00	0,00	0,00	0,12	0,00	**1,00**	0,02	0,02	0,00	0,02	0,00	0,00	0,00	0,02	**1,00**	0,00	0,00	0,00	0,00	0,00	0,00	0,00	0,00	0,00	0,00
0,00	0,00	0,00	0,00	0,00	0,00	0,00	0,00	0,00	0,00	0,00	0,00	0,00	0,00	0,00	0,15	0,00	0,00	0,00	0,00	0,00	0,00	0,00	0,00	0,00	0,00	0,00	0,00	0,00	0,02
0,00	0,00	0,29	0,00	0,00	0,00	0,00	0,00	0,00	0,00	0,00	0,00	0,00	0,00	0,00	0,19	0,04	0,00	0,00	0,00	0,00	0,08	0,00	0,00	0,00	0,00	0,00	0,00	0,00	0,00
0,00	0,00	0,00	0,00	0,00	**1,00**	0,00	0,00	0,21	0,00	0,00	0,00	0,06	0,00	0,00	0,04	0,00	0,19	0,00	0,00	0,00	0,00	0,37	0,00	0,04	0,00	0,00	0,00	0,00	0,27
0,00	0,00	0,04	0,00	0,00	0,00	**1,00**	0,00	0,00	0,00	0,00	0,00	0,00	0,00	0,00	0,00	0,00	0,00	0,00	0,00	0,62	0,85	0,00	0,00	0,00	0,00	0,00	0,00	0,69	0,00
0,00	0,00	0,25	0,00	0,00	0,00	0,00	0,00	0,00	0,00	0,00	0,02	0,00	0,00	0,00	0,00	0,00	0,00	0,00	0,00	0,27	0,02	0,00	**1,00**	0,00	0,00	0,00	0,00	0,15	0,02
0,00	**0,96**	0,00	0,00	0,00	0,00	0,00	0,00	0,00	0,00	0,00	0,00	0,00	0,00	0,00	0,00	0,00	0,02	0,00	0,00	0,00	0,00	0,06	0,00	0,00	0,00	0,00	**1,00**	0,00	0,00
0,00	0,00	0,00	0,00	0,00	0,00	0,00	0,00	0,00	0,00	0,00	0,00	0,00	0,00	0,67	0,00	0,00	0,00	0,00	0,00	0,00	0,00	0,00	0,00	0,00	0,00	0,00	0,00	0,13	0,00
0,02	0,00	0,00	0,00	0,00	0,00	0,00	0,00	0,08	0,06	0,00	0,00	0,02	0,00	0,00	0,00	0,00	0,02	0,00	0,00	0,00	0,00	0,06	0,00	0,00	0,00	0,00	0,00	0,00	0,10
0,00	0,00	0,00	0,00	0,00	0,00	0,00	0,00	0,42	0,00	0,00	0,00	0,00	0,00	0,00	0,35	0,00	0,04	0,00	0,00	0,00	0,00	0,17	0,00	**0,96**	0,00	0,00	0,00	0,00	0,04
0,00	0,04	0,00	0,02	0,00	0,00	0,00	0,02	0,04	0,44	0,00	0,63	0,12	0,00	0,15	0,02	0,00	0,00	0,04	0,00	0,10	0,00	0,00	0,00	0,00	0,00	0,00	0,00	0,00	0,13
0,00	0,00	0,23	0,00	0,00	0,00	0,00	**0,98**	0,00	0,48	0,00	0,17	0,38	0,00	0,02	0,04	0,00	0,13	0,13	0,00	0,00	0,00	0,00	0,00	0,00	0,00	0,00	0,00	0,02	0,00
0,00	0,00	0,19	0,00	0,00	0,00	0,00	0,00	0,00	0,00	0,00	0,00	0,02	**0,98**	0,00	0,21	**0,96**	0,00	0,00	0,00	0,00	0,02	0,00	0,00	0,00	0,00	0,92	0,00	0,00	0,00
0,00	0,00	0,00	0,00	0,00	0,00	0,00	0,00	0,00	0,00	0,00	0,00	0,00	0,00	0,00	0,00	0,00	0,00	0,00	0,00	0,00	0,00	0,00	0,00	0,00	0,00	0,00	0,00	0,00	0,00
0,00	0,00	0,00	0,00	0,00	0,00	0,00	0,00	0,00	0,00	0,00	0,00	0,00	0,00	0,00	0,00	0,00	0,00	0,00	0,00	0,00	0,00	0,00	0,00	0,00	0,31	0,00	0,00	0,00	0,00
0,00	0,00	0,00	0,00	0,00	0,00	0,00	0,00	0,00	0,00	0,00	0,00	0,00	0,00	0,00	0,00	0,00	0,00	0,00	0,00	0,00	0,00	0,00	0,00	0,00	0,00	0,00	0,00	0,00	0,00
yes	yes	-	yes	yes	yes	yes	yes	-	-	yes	-	-	yes	-	-	yes	-	-	yes	-	-	-	yes	yes	-	yes	yes	-	-
169	168	167	166	165	164	163	162	161	160	159	158	157	156	155	154	153	152	151	150	149	148	147	146	145	144	143	142	141	140
-	-	-	-	-	-	yes	-	-	-	-	-	-	-	-	-	-	-	-	-	-	-	-	-	-	-	-	yes	-	-
167	166	165	164	163	162	161	160	159	158	157	156	155	154	153	152	151	150	149	148	147	146	145	144	143	142	141	140	139	138
yes	-	-	yes	-	-	yes	-	-	-	-	-	-	-	-	-	-	-	-	-	-	-	-	-	-	-	-	-	-	-
-	-	-	-	-	-	yes	-	-	-	-	-	-	-	-	-	-	-	-	-	-	-	-	-	-	-	-	-	-	-
-	-	-	-	-	-	-	-	-	-	-	-	-	-	-	-	-	-	-	-	-	-	-	-	-	-	-	-	-	-
-	-	-	-	-	-	-	-	-	-	-	-	-	-	-	-	-	-	-	-	-	-	-	-	-	-	-	-	-	-

204	203	202	201	200	199	198	197	196	195	194	193	192	191	190	189	188	187	186	185	184	183	182	181	180	179	178	177	176	175
0,00	0,04	0,00	0,19	0,00	0,00	0,00	0,00	0,06	0,00	0,29	0,21	0,00	0,00	0,00	0,00	0,00	0,02	0,00	0,00	0,02	0,00	0,00	0,12	0,06	0,00	0,00	0,00	0,00	**1,00**
0,00	0,00	0,00	0,00	0,00	0,00	0,00	0,00	0,00	0,00	0,00	0,04	0,00	0,00	0,00	0,08	0,00	0,00	0,00	0,00	0,00	0,02	0,00	0,00	0,02	0,00	0,00	0,00	0,00	0,00
0,00	0,00	0,00	0,00	0,00	0,00	0,00	0,00	0,00	0,00	0,00	0,00	0,00	0,00	0,00	0,00	0,00	0,00	0,00	0,56	0,23	0,00	0,00	0,00	0,00	0,00	0,87	0,00	0,00	0,00
0,00	0,00	0,00	0,06	0,00	0,00	0,00	0,00	0,00	0,00	0,00	0,00	0,00	0,00	0,00	0,00	0,00	0,00	0,00	0,00	0,27	0,00	0,02	0,00	0,00	0,00	0,06	0,00	0,00	0,00
0,00	0,00	0,00	0,00	0,00	0,08	0,00	0,00	0,00	0,00	0,00	0,00	0,00	0,00	0,00	0,00	0,00	0,40	0,00	0,00	0,00	0,00	0,00	0,00	0,00	0,00	0,00	0,00	0,12	0,00
0,00	0,00	0,00	0,00	0,00	0,00	0,00	0,00	0,00	**1,00**	0,00	0,00	0,00	**1,00**	0,00	0,00	0,00	0,00	0,00	0,00	0,04	0,00	0,00	0,00	0,00	0,00	0,02	0,00	0,00	0,00
0,00	0,00	0,00	0,00	0,00	0,00	0,00	**0,96**	0,00	0,00	0,00	0,00	0,00	0,00	0,00	0,00	0,00	0,38	0,00	0,00	0,06	0,00	0,06	0,00	0,00	0,00	0,02	0,00	0,00	0,00
0,00	0,02	0,23	0,00	0,00	0,00	0,00	0,00	0,00	0,00	0,00	0,00	0,00	0,00	0,65	0,40	0,00	0,00	0,00	0,00	0,00	0,13	0,00	0,00	0,10	0,00	0,00	0,00	0,21	0,00
0,10	0,00	0,00	0,04	0,00	0,00	0,00	0,00	0,00	0,00	0,00	0,00	0,00	0,00	0,00	0,00	0,00	0,00	0,00	0,00	0,02	0,00	0,00	0,02	0,00	0,00	0,00	0,00	0,00	0,00
0,00	0,00	0,13	0,02	0,00	0,00	0,00	0,00	0,00	0,00	0,10	0,00	0,00	0,00	0,33	0,42	0,00	0,00	0,00	0,00	0,02	0,04	0,00	0,00	0,00	0,00	0,00	0,00	0,29	0,00
0,00	0,00	0,63	0,38	0,00	0,00	0,00	0,00	0,06	0,00	0,19	0,00	0,00	0,00	0,00	0,00	0,00	0,00	0,00	0,00	0,00	0,00	0,00	0,00	0,00	0,00	0,00	0,00	0,38	0,00
0,00	0,00	0,00	0,00	0,00	0,00	0,00	0,00	0,00	0,00	0,00	0,00	0,00	0,00	0,00	0,00	0,00	0,00	0,00	0,19	0,02	0,00	0,85	0,00	0,00	0,00	0,02	0,00	0,00	0,00
0,00	0,00	0,00	0,00	**1,00**	0,00	**1,00**	0,00	0,87	0,00	0,00	0,00	0,00	0,00	0,00	0,00	0,00	0,00	0,00	0,00	0,00	0,04	0,00	0,00	0,00	0,00	0,00	0,00	0,00	0,00
0,00	0,00	0,00	0,00	0,00	0,00	0,00	0,00	0,00	0,00	0,00	0,00	0,00	0,00	0,00	0,00	0,00	0,00	0,00	0,00	0,00	0,00	0,00	0,00	0,00	0,00	0,00	0,00	0,00	0,00
0,90	0,00	0,00	0,02	0,00	0,00	0,00	0,04	0,00	0,00	0,00	0,00	0,00	0,00	0,00	0,00	0,00	0,00	0,00	0,00	0,33	0,00	0,04	0,00	0,00	0,00	0,00	**1,00**	0,00	0,00
0,00	0,00	0,00	0,04	0,00	0,00	0,00	0,00	0,02	0,00	0,00	0,00	0,69	0,00	0,00	0,00	0,00	0,00	0,08	0,02	0,00	0,00	0,02	0,17	0,02	0,00	0,00	0,00	0,00	0,00
0,00	0,00	0,00	0,21	0,00	0,00	0,00	0,00	0,00	0,00	0,00	0,12	0,31	0,00	0,00	0,00	0,00	0,00	0,92	0,21	0,00	0,00	0,02	0,69	0,04	0,00	0,00	0,00	0,00	0,00
0,00	0,94	0,00	0,04	0,00	0,00	0,00	0,00	0,00	0,00	0,40	0,63	0,00	0,00	0,02	0,10	0,00	0,00	0,00	0,00	0,00	0,77	0,00	0,00	0,77	0,00	0,00	0,00	0,00	0,00
0,00	0,00	0,00	0,00	0,00	0,00	0,00	0,00	0,00	0,00	0,00	0,00	0,00	0,00	0,00	0,00	0,00	0,00	0,00	0,00	0,00	0,00	0,00	0,00	0,00	**1,00**	0,00	0,00	0,00	0,00
0,00	0,00	0,00	0,00	0,00	0,92	0,00	0,00	0,00	0,00	0,02	0,00	0,00	0,00	0,00	0,00	**1,00**	0,19	0,00	0,02	0,00	0,00	0,00	0,00	0,00	0,00	0,02	0,00	0,00	0,00
0,00	0,00	0,00	0,00	0,00	0,00	0,00	0,00	0,00	0,00	0,00	0,00	0,00	0,00	0,00	0,00	0,00	0,00	0,00	0,00	0,00	0,00	0,00	0,00	0,00	0,00	0,00	0,00	0,00	0,00
yes	yes	-	-	yes	yes	yes	yes	-	yes	-	-	-	yes	-	-	yes	-	yes	-	-	-	-	-	-	yes	-	yes	-	yes
199	198	197	196	195	194	193	192	191	190	189	188	187	186	185	184	183	182	181	180	179	178	177	176	175	174	173	172	171	170
-	-	-	-	-	-	-	-	-	-	-	yes	yes	-	yes	yes	yes	yes	yes	-	-	-	-	-	-	-	-	-	-	-
197	196	195	194	193	192	191	190	189	188	187	186	185	184	183	182	181	180	179	178	177	176	175	174	173	172	171	170	169	168
-	yes	yes	-	-	yes	-	yes	-	yes	yes	yes	yes	-	yes	yes	yes	yes	yes	-	-	-	-	-	-	-	-	-	-	-
-	-	-	-	-	-	-	-	-	-	-	yes	yes	-	yes	yes	yes	yes	yes	-	-	-	-	-	-	-	-	-	-	-
-	-	-	-	-	-	-	-	-	-	-	-		-	-	-	-	-	-	-	-	-	-	-	-	-	-	-	-	-
-	-	-	-	-	-	-	-	-	-	-	-	yes	-	-	-	-	-	-	-	-	-	-	-	-	-	-	-	-	-

234	233	232	231	230	229	228	227	226	225	224	223	222	221	220	219	218	217	216	215	214	213	212	211	210	209	208	207	206	205
0,10	0,04	**1,00**	0,00	0,04	0,40	0,00	0,00	0,00	0,02	0,00	0,00	0,04	0,13	0,10	0,00	0,04	0,00	0,19	0,02	0,37	0,00	0,00	0,00	0,00	0,00	0,13	0,00	0,00	0,00
0,00	**0,96**	0,00	0,00	0,00	0,08	0,00	0,00	0,00	0,00	0,00	0,00	0,00	0,00	0,00	0,00	0,06	0,00	0,00	0,00	0,02	0,00	0,00	0,00	0,00	0,00	0,02	0,00	0,00	0,00
0,00	0,00	0,00	0,00	0,00	0,00	**0,96**	0,00	0,00	0,02	0,00	0,00	0,00	0,00	0,04	0,00	0,00	0,00	0,02	0,00	0,00	0,00	0,08	0,00	0,00	0,00	0,00	0,00	0,00	0,87
0,00	0,00	0,00	0,00	0,00	0,02	0,00	0,00	0,00	0,02	0,04	0,02	0,81	0,02	0,69	0,00	0,00	0,02	0,25	0,00	0,00	**1,00**	0,33	0,00	0,00	0,00	0,00	0,00	0,00	0,04
0,00	0,00	0,00	0,02	0,00	0,00	0,00	0,00	0,00	0,00	0,00	0,00	0,00	0,00	0,00	0,00	0,02	0,00	0,00	0,00	0,00	0,00	0,00	0,00	0,00	0,00	0,00	0,00	0,94	0,02
0,00	0,00	0,00	0,00	0,00	0,00	0,00	0,00	0,00	0,00	0,04	0,94	0,00	0,00	0,00	0,00	0,00	0,00	0,00	0,00	0,00	0,00	0,00	**1,00**	0,00	0,00	0,00	0,00	0,00	0,00
0,00	0,00	0,00	0,00	0,00	0,00	0,00	0,00	0,00	0,00	0,00	0,00	0,00	0,02	0,00	0,00	0,00	0,00	0,00	0,00	0,00	0,00	0,02	0,00	0,00	0,00	0,00	0,06	0,00	0,06
0,12	0,00	0,00	0,31	0,40	0,00	0,00	0,00	0,00	0,00	0,00	0,00	0,04	0,04	0,00	0,02	0,48	0,00	0,00	0,00	0,04	0,00	0,02	0,00	**0,96**	0,06	0,00	0,00	0,00	0,00
0,00	0,00	0,00	0,00	0,00	0,00	0,00	0,00	0,00	0,00	0,13	0,02	0,00	0,38	0,06	0,02	0,00	0,00	0,10	0,42	0,00	0,00	0,12	0,00	0,00	0,00	0,08	0,00	0,00	0,00
0,00	0,00	0,00	0,04	0,23	0,06	0,00	0,00	0,71	0,00	0,00	0,00	0,04	0,04	0,00	0,63	0,17	0,00	0,00	0,00	0,04	0,00	0,02	0,00	0,00	0,00	0,00	0,00	0,06	0,00
0,00	0,00	0,00	0,08	0,00	0,04	0,00	0,00	0,04	0,00	0,00	0,00	0,00	0,04	0,00	0,15	0,08	0,00	0,00	0,00	0,00	0,00	0,00	0,00	0,00	0,00	0,00	0,00	0,00	0,00
0,00	0,00	0,00	0,00	0,00	0,00	0,02	0,00	0,02	0,02	0,00	0,02	0,00	0,00	0,02	0,00	0,00	0,00	0,00	0,00	0,00	0,00	0,08	0,00	0,00	0,00	0,00	0,00	0,00	0,00
0,00	0,00	0,00	0,00	0,00	0,00	0,00	**1,00**	0,00	0,00	0,00	0,00	0,00	0,00	0,00	0,02	0,00	0,00	0,00	0,00	0,00	0,00	0,00	0,00	0,00	0,00	0,00	0,00	0,00	0,00
0,00	0,00	0,00	0,00	0,00	0,00	0,00	0,00	0,04	0,00	0,00	0,00	0,00	0,13	0,02	0,12	0,00	**0,98**	0,17	0,00	0,00	0,00	0,04	0,00	0,00	0,00	0,00	0,94	0,00	0,00
0,00	0,00	0,00	0,00	0,00	0,00	0,00	0,00	0,00	0,00	0,79	0,00	0,00	0,15	0,04	0,02	0,00	0,00	0,21	0,56	0,00	0,00	0,27	0,00	0,00	0,00	0,13	0,00	0,00	0,00
0,00	0,00	0,00	0,00	0,00	0,10	0,00	0,00	0,00	0,08	0,00	0,00	0,00	0,00	0,04	0,00	0,08	0,00	0,00	0,00	0,00	0,00	0,00	0,00	0,00	0,00	0,62	0,00	0,00	0,00
0,00	0,00	0,00	0,00	0,00	0,13	0,02	0,00	0,02	0,02	0,00	0,00	0,02	0,02	0,00	0,02	0,00	0,00	0,00	0,00	0,27	0,00	0,02	0,00	0,00	0,00	0,02	0,00	0,00	0,00
0,79	0,00	0,00	0,56	0,33	0,13	0,00	0,00	0,13	0,00	0,00	0,00	0,02	0,02	0,00	0,00	0,08	0,00	0,06	0,00	0,27	0,00	0,02	0,00	0,04	0,94	0,00	0,00	0,00	0,00
0,00	0,00	0,00	0,00	0,00	0,00	0,00	0,00	0,00	0,00	0,00	0,00	0,00	0,00	0,00	0,00	0,00	0,00	0,00	0,00	0,00	0,00	0,00	0,00	0,00	0,00	0,00	0,00	0,00	0,00
0,00	0,00	0,00	0,00	0,00	0,04	0,00	0,00	0,04	0,00	0,00	0,00	0,04	0,00	0,00	0,00	0,00	0,00	0,00	0,00	0,00	0,00	0,00	0,00	0,00	0,00	0,00	0,00	0,00	0,02
0,00	0,00	0,00	0,00	0,00	0,00	0,00	0,00	0,00	0,83	0,00	0,00	0,00	0,00	0,00	0,00	0,00	0,00	0,00	0,00	0,00	0,00	0,00	0,00	0,00	0,00	0,00	0,00	0,00	0,00
-	yes	yes	-	-	-	yes	yes	-	-	-	yes	-	-	-	-	-	yes	-	-	-	yes	-	yes	yes	yes	-	yes	yes	-
228	227	226	225	224	223	222	221	220	-	219	218	217	216	215	214	213	212	211	210	209	208	207	206	205	204	203	202	201	200
-	-	yes	-	-	-	-	yes	yes	-	-	-	-	-	-	-	-	-	-	-	-	-	-	-	-	-	-	-	yes	-
226	225	224	223	222	221	220	219	218	-	217	216	215	214	213	212	211	210	209	208	207	206	205	204	203	202	201	200	199	198
-	-	yes	-	-	-	-	-	-	-	-	-	-	-	-	-	-	-	-	-	-	-	yes	-	-	yes	-	yes	-	-
-	-	yes	-	-	-	-	-	-	-	-	-	-	-	-	-	-	-	-	-	-	-	-	-	-	-	-	-	-	-
-	-	-	-	-	-	-	-	-	-	-	-	-	-	-	-	-	-	-	-	-	-	-	-	-	-	-	-	-	-
-	-	-	-	-	-	-	-	-	-	-	-	-	-	-	-	-	-	-	-	-	-	-	-	-	-	-	-	-	-

264	263	262	261	260	259	258	257	256	255	254	253	252	251	250	249	248	247	246	245	244	243	242	241	240	239	238	237	236	235
0,50	0,92	0,00	0,12	0,12	0,00	0,00	0,08	0,00	0,04	0,04	0,02	0,08	0,00	0,00	0,00	0,21	0,00	0,08	0,21	0,00	0,06	0,00	0,02	0,94	0,00	0,00	0,00	0,00	0,00
0,00	0,00	0,00	0,00	0,00	0,00	0,00	0,00	0,00	0,00	0,00	0,00	0,02	0,00	0,00	0,00	0,00	0,00	0,00	0,00	0,00	0,00	0,00	0,00	0,00	0,00	0,00	0,00	0,00	0,00
0,00	0,00	0,00	0,00	0,00	0,00	0,00	0,04	0,00	0,27	0,04	0,73	0,21	0,00	0,00	0,00	0,00	0,00	0,12	0,00	0,00	0,00	0,00	0,00	0,00	0,00	0,00	0,00	0,00	0,00
0,00	0,00	1,00	0,00	0,00	0,00	0,00	0,19	0,00	0,10	0,29	0,15	0,31	0,00	0,00	0,00	0,00	0,00	0,04	0,00	0,00	0,00	0,00	0,00	0,00	0,00	0,00	0,00	0,00	0,00
0,00	0,00	0,00	0,00	0,00	0,04	0,00	0,00	0,00	0,00	0,00	0,00	0,00	0,00	0,00	0,00	0,00	0,90	0,00	0,00	1,00	0,00	0,00	0,00	0,00	0,00	0,00	0,00	0,00	0,00
0,48	0,08	0,00	0,00	0,87	0,00	0,00	0,00	0,00	0,15	0,02	0,04	0,06	0,00	0,00	0,00	0,00	0,00	0,06	0,00	0,00	0,00	1,00	0,00	0,00	0,00	0,00	1,00	1,00	1,00
0,00	0,00	0,00	0,00	0,00	0,04	0,00	0,00	0,00	0,00	0,00	0,00	0,00	0,00	0,00	0,00	0,00	0,00	0,00	0,35	0,00	0,00	0,00	0,00	0,00	0,00	0,00	0,00	0,00	0,00
0,00	0,00	0,00	0,02	0,00	0,60	0,06	0,00	0,00	0,00	0,00	0,00	0,00	0,00	0,00	0,00	0,33	0,00	0,00	0,00	0,00	0,44	0,00	0,63	0,00	0,00	0,00	0,00	0,00	0,00
0,00	0,00	0,00	0,00	0,00	0,00	0,00	0,15	0,00	0,02	0,12	0,00	0,02	0,00	0,00	0,00	0,00	0,00	0,00	0,00	0,00	0,00	0,00	0,00	0,00	0,00	0,00	0,00	0,00	0,00
0,00	0,00	0,00	0,00	0,00	0,00	0,69	0,02	0,00	0,00	0,00	0,00	0,00	0,00	0,13	0,00	0,31	0,00	0,00	0,00	0,00	0,33	0,00	0,00	0,00	0,00	0,00	0,00	0,00	0,00
0,00	0,00	0,00	0,00	0,00	0,00	0,25	0,00	0,00	0,00	0,00	0,00	0,00	0,00	0,00	0,00	0,02	0,02	0,00	0,00	0,00	0,10	0,00	0,35	0,00	0,00	0,00	0,00	0,00	0,00
0,00	0,00	0,00	0,00	0,00	0,00	0,00	0,00	0,00	0,04	0,02	0,00	0,10	0,00	0,00	0,00	0,00	0,00	0,00	0,00	0,00	0,00	0,00	0,00	0,00	1,00	0,00	0,00	0,00	0,00
0,00	0,00	0,00	0,00	0,02	0,00	0,00	0,00	0,19	0,00	0,10	0,02	0,17	0,12	0,00	0,00	0,00	0,00	0,71	0,00	0,00	0,00	0,00	0,00	0,00	0,00	0,00	0,00	0,00	0,00
0,00	0,00	0,00	0,00	0,00	0,00	0,00	0,04	0,00	0,00	0,08	0,02	0,04	0,00	0,00	0,00	0,00	0,00	0,00	0,00	0,00	0,00	0,00	0,00	0,00	0,00	0,00	0,00	0,00	0,00
0,00	0,00	0,00	0,00	0,00	0,00	0,00	0,48	0,00	0,17	0,08	0,00	0,00	0,00	0,00	0,00	0,00	0,00	0,00	0,00	0,00	0,00	0,00	0,00	0,00	0,00	0,00	0,00	0,00	0,00
0,02	0,00	0,00	0,00	0,00	0,00	0,00	0,00	0,00	0,21	0,02	0,00	0,00	0,00	0,00	0,00	0,00	0,00	0,00	0,10	0,00	0,00	0,00	0,00	0,06	0,00	1,00	0,00	0,00	0,00
0,00	0,00	0,00	0,06	0,00	0,00	0,00	0,00	0,00	0,00	0,04	0,00	0,00	0,00	0,00	0,00	0,00	0,00	0,00	0,00	0,00	0,04	0,00	0,00	0,00	0,00	0,00	0,00	0,00	0,00
0,00	0,00	0,00	0,81	0,00	0,19	0,00	0,00	0,81	0,00	0,00	0,02	0,00	0,00	0,00	0,00	0,13	0,00	0,00	0,00	0,00	0,04	0,00	0,00	0,00	0,00	0,00	0,00	0,00	0,00
0,00	0,00	0,00	0,00	0,00	0,00	0,00	0,00	0,00	0,00	0,00	0,00	0,00	0,00	0,00	0,00	0,00	0,00	0,00	0,02	0,00	0,00	0,00	0,00	0,00	0,00	0,00	0,00	0,00	0,00
0,00	0,00	0,00	0,00	0,00	0,13	0,00	0,00	0,00	0,00	0,00	0,00	0,00	0,00	0,00	0,17	0,00	0,08	0,00	0,33	0,00	0,00	0,00	0,00	0,00	0,00	0,00	0,00	0,00	0,00
0,00	0,00	0,00	0,00	0,00	0,00	0,00	0,00	0,00	0,00	0,17	0,00	0,00	0,88	0,87	0,83	0,00	0,00	0,00	0,00	0,00	0,00	0,00	0,00	0,00	0,00	0,00	0,00	0,00	0,00
-	yes	yes	-	-	-	-	-	-	-	-	-	-	-	-	-	-	yes	-	-	yes	-	yes	-	yes	yes	yes	yes	yes	yes
255	254	253	252	251	250	249	248	247	246	245	244	243	-	-	-	242	241	240	239	238	237	236	235	234	233	232	231	230	229
-	-	-	-	-	-	-	-	-	-	-	-	-	-	-	-	-	-	-	-	-	-	-	-	yes	-	-	-	-	-
253	252	251	250	249	248	247	246	245	244	243	242	241	-	-	-	240	239	238	237	236	235	234	233	232	231	230	229	228	227
yes	yes	yes	yes	yes	-	-	-	-	-	-	-	-	-	-	-	-	-	-	-	-	-	-	-	yes	-	-	-	-	-
-	-	-	-	-	-	-	-	-	-	-	-	-	-	-	-	-	-	-	-	-	-	-	-	yes	-	-	-	-	-
-	-	-	-	-	-	-	-	-	-	-	-	-	-	-	-	-	-	-	-	-	-	-	-	-	-	-	-	-	-
-	-	-	-	-	-	-	-	-	-	-	-	-	-	-	-	-	-	-	-	-	-	-	-	-	-	-	-	-	-

294	293	292	291	290	289	288	287	286	285	284	283	282	281	280	279	278	277	276	275	274	273	272	271	270	269	268	267	266	265
0,00	0,00	0,31	0,00	0,00	0,00	0,00	0,00	0,00	0,02	0,00	0,37	0,13	0,00	0,06	**0,96**	0,60	0,00	0,00	0,10	0,08	0,00	0,02	0,04	0,00	0,04	0,02	0,06	0,00	0,00
0,00	0,00	0,00	0,00	0,00	0,00	0,00	0,00	0,00	0,00	0,00	0,02	0,25	0,00	0,00	0,00	0,00	0,00	0,00	0,00	0,00	0,00	0,00	0,00	0,06	0,00	0,00	0,00	0,02	0,00
0,00	0,00	0,00	0,00	0,00	0,00	0,00	0,00	0,06	0,02	0,00	0,02	0,00	0,00	0,00	0,00	0,00	0,00	0,00	0,00	0,00	0,02	0,08	0,08	0,00	0,25	0,00	0,00	0,10	0,00
0,00	0,04	0,00	0,00	0,00	0,00	0,00	0,00	0,13	0,15	0,00	0,00	0,02	0,00	0,00	0,00	0,00	0,00	0,00	0,00	0,02	0,00	0,29	0,06	0,00	0,42	0,00	0,00	0,23	0,00
0,00	0,02	0,02	0,00	0,00	0,08	0,00	0,00	0,00	0,00	0,00	0,00	0,00	0,08	0,00	0,00	0,00	0,00	0,00	0,00	0,00	0,00	0,00	0,00	0,00	0,00	0,00	0,00	0,00	0,00
0,00	0,00	0,00	**1,00**	0,00	0,00	0,00	**1,00**	0,00	0,00	**1,00**	0,31	0,08	0,00	0,00	0,00	0,19	0,00	0,00	0,00	0,00	0,00	0,00	0,56	0,02	0,06	0,00	0,94	0,00	**1,00**
0,00	0,02	0,00	0,00	0,87	0,00	0,00	0,00	0,00	0,00	0,00	0,00	0,00	0,00	0,00	0,00	0,00	0,90	0,00	0,00	0,00	0,00	0,00	0,00	0,00	0,00	0,00	0,00	0,15	0,00
0,02	0,00	0,00	0,00	0,00	0,04	0,35	0,00	0,02	0,02	0,00	0,04	0,00	0,23	0,00	0,00	0,00	0,00	0,00	0,00	0,00	0,00	0,00	0,00	0,00	0,02	0,38	0,00	0,00	0,00
0,00	0,19	0,00	0,00	0,00	0,00	0,00	0,00	0,04	0,21	0,00	0,12	0,00	0,00	0,00	0,00	0,00	0,00	0,00	0,00	0,00	0,06	0,23	0,00	0,02	0,06	0,02	0,00	0,27	0,00
0,00	0,21	0,00	0,00	0,00	0,87	0,00	0,00	0,06	0,00	0,00	0,00	0,00	0,60	0,00	0,02	0,00	0,00	0,10	0,00	0,00	0,00	0,04	0,02	0,00	0,00	0,29	0,00	0,06	0,00
0,19	0,17	0,10	0,00	0,00	0,00	0,00	0,00	0,00	0,00	0,00	0,00	0,00	0,00	0,00	0,00	0,00	0,00	0,00	0,00	0,00	0,00	0,04	0,00	0,00	0,00	0,04	0,00	0,00	0,00
0,00	0,00	0,21	0,00	0,00	0,00	0,00	0,00	0,00	0,00	0,00	0,02	0,12	0,00	0,00	0,00	0,00	0,04	0,00	0,00	0,00	0,00	0,06	0,04	0,02	0,02	0,00	0,00	0,00	0,00
0,00	0,00	0,00	0,00	0,00	0,00	0,00	0,00	0,40	0,00	0,00	0,00	0,00	0,00	0,10	0,00	0,00	0,00	0,00	0,00	0,00	0,00	0,00	0,06	0,06	0,00	0,00	0,00	0,00	0,00
0,00	0,00	0,00	0,00	0,13	0,00	0,00	0,00	0,00	0,00	0,00	0,00	0,04	0,00	0,00	0,00	0,00	0,00	0,00	0,00	0,00	0,00	0,02	0,02	0,02	0,00	0,00	0,00	0,00	0,00
0,00	0,29	0,00	0,00	0,00	0,00	0,00	0,00	0,06	0,33	0,00	0,04	0,00	0,00	0,08	0,00	0,00	0,00	0,00	0,00	0,00	0,02	0,23	0,00	0,02	0,04	0,00	0,00	0,10	0,00
0,37	0,00	0,29	0,00	0,00	0,00	0,00	0,00	0,00	0,15	0,00	0,00	0,19	0,00	0,44	0,02	0,21	0,00	0,00	0,00	0,00	0,00	0,00	0,00	0,21	0,08	0,00	0,00	0,04	0,00
0,42	0,00	0,08	0,00	0,00	0,00	0,00	0,00	0,00	0,10	0,00	0,04	0,15	0,00	0,33	0,00	0,00	0,06	0,00	0,00	0,00	0,00	0,00	0,10	0,56	0,02	0,04	0,00	0,00	0,00
0,00	0,00	0,00	0,00	0,00	0,02	0,65	0,00	0,23	0,00	0,00	0,02	0,02	0,10	0,00	0,00	0,00	0,00	0,00	0,00	0,00	0,00	0,00	0,00	0,00	0,00	0,21	0,00	0,00	0,00
0,00	0,00	0,00	0,00	0,00	0,00	0,00	0,00	0,00	0,00	0,00	0,02	0,00	0,00	0,00	0,00	0,00	0,00	0,00	0,00	0,00	0,00	0,00	0,00	0,00	0,00	0,00	0,00	0,00	0,00
0,00	0,06	0,00	0,00	0,00	0,00	0,00	0,00	0,00	0,00	0,00	0,00	0,00	0,00	0,00	0,00	0,00	0,00	0,00	0,00	0,00	0,00	0,00	0,00	0,02	0,00	0,00	0,00	0,04	0,00
0,00	0,00	0,00	0,00	0,00	0,00	0,00	0,00	0,00	0,00	0,00	0,00	0,00	0,00	0,00	0,00	0,00	0,00	0,90	0,90	0,90	0,90	0,00	0,04	0,00	0,00	0,00	0,00	0,00	0,00
-	-	-	yes	-	-	-	yes	-	-	yes	-	-	-	-	yes	-	yes	-	-	-	-	-	-	-	-	-	yes	-	yes
281	280	279	278	277	276	275	274	273	272	271	270	269	268	267	266	265	264	-	-	-	-	263	262	261	260	259	258	257	256
yes	yes	yes	-	-	-	-	-	-	-	-	-	-	-	-	-	-	-	-	-	-	-	-	-	-	-	-	-	-	-
279	278	277	276	275	274	273	272	271	270	269	268	267	266	265	264	263	262	-	-	-	-	261	260	259	258	257	256	255	254
yes	yes	yes	-	-	-	-	-	-	-	-	-	-	-	yes	-	yes	-	-	-	-	-	-	-	-	-	-	-	-	-
yes	yes	yes	-	-	-	-	-	-	-	-	-	-	-	-	-	-	-	-	-	-	-	-	-	-	-	-	-	-	-
-	yes	yes	-	-	-	-	-	-	-	-	-	-	-	-	-	-	-	-	-	-	-	-	-	-	-	-	-	-	-
-	yes	yes	-	-	-	-	-	-	-	-	-	-	-	-	-	-	-	-	-	-	-	-	-	-	-	-	-	-	-

324	323	322	321	320	319	318	317	316	315	314	313	312	311	310	309	308	307	306	305	304	303	302	301	300	299	298	297	296	295
0,54	0,00	0,00	0,00	0,00	0,00	0,08	0,08	0,00	0,00	0,00	0,00	1,00	0,10	0,00	0,00	0,00	0,38	0,00	0,04	0,00	0,00	0,00	0,00	0,10	0,00	0,04	0,00	0,00	0,00
0,08	0,00	0,00	0,00	0,00	0,00	0,00	0,00	0,00	0,00	0,00	0,00	0,00	0,00	0,00	0,02	0,00	0,00	0,00	0,00	0,00	0,00	0,00	0,00	0,00	0,00	0,02	0,00	0,00	0,00
0,00	0,00	0,00	0,00	0,00	0,00	0,00	0,00	0,00	1,00	0,00	0,00	0,00	0,00	0,00	0,00	0,00	0,00	0,12	0,02	0,00	0,00	0,00	0,52	0,23	0,88	0,00	0,00	0,00	0,00
0,00	0,00	1,00	0,00	0,00	0,00	0,00	0,00	0,00	0,00	0,00	0,00	0,00	0,00	0,00	0,00	0,06	0,00	0,42	0,10	0,00	0,00	0,00	0,12	0,46	0,00	0,08	0,00	0,00	0,00
0,00	0,00	0,00	0,00	0,00	0,00	0,00	0,00	0,00	0,00	0,00	0,00	0,00	0,00	0,00	0,00	0,00	0,00	0,00	0,00	0,00	0,00	0,00	0,06	0,00	0,00	0,02	0,00	0,06	0,04
0,00	0,00	0,00	0,00	0,98	0,00	0,92	0,00	0,00	0,00	0,00	1,00	0,00	0,00	0,00	0,00	0,00	0,00	0,04	0,02	0,00	0,00	1,00	0,02	0,06	0,00	0,00	0,00	0,00	0,00
0,00	0,87	0,00	0,00	0,00	0,00	0,00	0,00	0,00	0,00	0,00	0,00	0,00	0,00	0,00	0,00	0,73	0,00	0,02	0,00	0,00	0,00	0,00	0,04	0,00	0,00	0,04	0,00	0,00	0,00
0,00	0,00	0,00	0,00	0,00	0,31	0,00	0,00	0,00	0,00	0,00	0,00	0,00	0,00	0,52	0,00	0,00	0,00	0,00	0,23	0,87	0,00	0,00	0,00	0,00	0,00	0,00	0,04	0,08	0,02
0,00	0,00	0,00	0,00	0,00	0,00	0,00	0,00	0,00	0,00	0,00	0,00	0,00	0,00	0,00	0,00	0,00	0,00	0,00	0,02	0,00	0,00	0,00	0,00	0,06	0,00	0,00	0,04	0,00	0,08
0,00	0,13	0,00	0,00	0,00	0,00	0,00	0,00	0,00	0,00	1,00	0,00	0,00	0,00	0,00	0,00	0,00	0,00	0,08	0,25	0,00	0,00	0,00	0,00	0,00	0,00	0,00	0,79	0,62	0,02
0,00	0,00	0,00	0,00	0,00	0,00	0,00	0,00	0,00	0,00	0,00	0,00	0,00	0,00	0,00	0,00	0,00	0,00	0,00	0,04	0,00	0,00	0,00	0,00	0,00	0,00	0,00	0,13	0,00	0,00
0,00	0,00	0,00	0,00	0,00	0,00	0,00	0,00	0,00	0,00	0,00	0,00	0,00	0,00	0,00	0,00	0,00	0,00	0,00	0,08	0,00	0,00	0,00	0,19	0,02	0,08	0,06	0,00	0,00	0,00
0,00	0,00	0,00	1,00	0,00	0,00	0,00	0,83	0,00	0,00	0,00	0,00	0,00	0,00	0,00	0,00	0,00	0,06	0,31	0,00	0,00	0,00	0,00	0,00	0,02	0,00	0,00	0,00	0,00	0,00
0,00	0,00	0,00	0,00	0,00	0,00	0,00	0,00	0,00	0,00	0,00	0,00	0,00	0,00	0,00	0,00	0,00	0,00	0,00	0,10	0,00	1,00	0,00	0,00	0,02	0,00	0,67	0,00	0,00	0,00
0,00	0,00	0,00	0,00	0,00	0,00	0,00	0,00	0,00	0,00	0,00	0,00	0,00	0,00	0,00	0,00	0,00	0,00	0,00	0,02	0,00	0,00	0,00	0,00	0,04	0,00	0,00	0,00	0,00	0,04
0,38	0,00	0,00	0,00	0,02	0,00	0,00	0,10	0,00	0,00	0,00	0,00	0,00	0,90	0,00	0,98	0,08	0,15	0,00	0,02	0,00	0,00	0,00	0,00	0,00	0,00	0,02	0,00	0,00	0,00
0,00	0,00	0,00	0,00	0,00	0,00	0,00	0,00	0,00	0,00	0,00	0,00	0,00	0,00	0,00	0,00	0,00	0,35	0,00	0,00	0,00	0,00	0,00	0,00	0,00	0,04	0,00	0,00	0,00	0,00
0,00	0,00	0,00	0,00	0,00	0,69	0,00	0,00	0,00	0,00	0,00	0,00	0,00	0,00	0,48	0,00	0,00	0,06	0,02	0,08	0,13	0,00	0,00	0,02	0,00	0,00	0,00	0,00	0,25	0,00
0,00	0,00	0,00	0,00	0,00	0,00	0,00	0,00	0,00	0,00	0,00	0,00	0,00	0,00	0,00	0,00	0,00	0,00	0,00	0,00	0,00	0,00	0,00	0,00	0,00	0,00	0,00	0,00	0,00	0,02
0,00	0,00	0,00	0,00	0,00	0,00	0,00	0,00	1,00	0,00	0,00	0,00	0,00	0,00	0,00	0,00	0,13	0,00	0,00	0,00	0,00	0,00	0,00	0,04	0,00	0,00	0,06	0,00	0,00	0,79
0,00	0,00	0,00	0,00	0,00	0,00	0,00	0,00	0,00	0,00	0,00	0,00	0,00	0,00	0,00	0,00	0,00	0,00	0,00	0,00	0,00	0,00	0,00	0,00	0,00	0,00	0,00	0,00	0,00	0,00
-	-	yes	yes	yes	-	yes	-	yes	yes	yes	yes	yes	yes	-	yes	-	-	-	-	-	yes	yes	-	-	-	-	-	-	-
311	310	309	308	307	306	305	304	303	302	301	300	299	298	297	296	295	294	293	292	291	290	289	288	287	286	285	284	283	282
-	-	-	-	yes	-	-	yes	yes	-	yes	yes	-	yes	-	-	-	-	-	-	-	-	-	-	-	-	yes	yes	yes	yes
309	308	307	306	305	304	303	302	301	300	299	298	297	296	295	294	293	292	291	290	289	288	287	286	285	284	283	282	281	280
-	-	-	-	yes	-	-	yes	yes	yes	yes	yes	yes	yes	-	-	-	-	-	-	-	-	-	-	-	-	-	-	yes	yes
-	-	-	-	yes	-	-	yes	yes	-	yes	yes	-	yes	-	-	-	-	-	-	-	-	-	-	-	-	-	-	yes	yes
-	-	-	-	-	-	-	-	-	-	-	-	-	-	-	-	-	-	-	-	-	-	-	-	-	-	-	-	-	-
-	-	-	-	-	-	-	-	-	-	-	-	-	-	-	-	-	-	-	-	-	-	-	-	-	-	-	-	-	-

354	353	352	351	350	349	348	347	346	345	344	343	342	341	340	339	338	337	336	335	334	333	332	331	330	329	328	327	326	325
0,00	0,00	0,02	0,00	0,06	0,00	0,04	0,00	0,00	0,79	0,21	0,00	0,88	0,00	0,17	0,00	0,00	0,21	0,25	0,04	0,10	0,02	0,58	0,04	0,00	0,10	0,00	0,15	0,00	0,00
0,00	0,00	0,02	0,00	0,25	0,08	0,00	0,00	0,00	0,00	0,00	0,00	0,00	0,00	0,00	0,00	0,00	0,04	0,02	0,02	0,00	0,00	0,00	0,00	0,00	0,02	0,00	0,00	0,00	0,00
0,00	0,00	0,00	0,00	0,00	0,00	0,02	0,02	0,00	0,00	0,23	0,00	0,00	0,04	0,40	**0,96**	0,02	0,00	0,02	0,00	0,00	0,02	0,00	0,00	0,00	0,00	0,65	0,02	0,00	0,00
0,00	**1,00**	0,02	0,08	0,00	0,00	0,13	0,08	0,00	0,00	0,38	0,00	0,00	0,85	0,12	0,00	0,00	0,00	0,00	0,10	0,00	0,44	0,00	0,00	0,02	0,04	0,23	0,00	0,00	0,00
0,00	0,00	0,02	0,00	0,00	0,02	0,02	0,00	0,92	0,00	0,00	0,00	0,00	0,00	0,00	0,00	0,00	0,00	0,00	0,00	0,00	0,00	0,00	0,00	0,00	0,00	0,00	0,00	0,02	0,15
**1,00**	0,00	0,00	0,00	0,00	0,00	0,00	0,00	0,00	0,21	0,00	0,00	0,00	0,00	0,02	0,00	0,00	0,00	0,02	0,02	0,00	0,00	0,00	0,00	0,83	0,00	0,00	0,00	0,00	0,00
0,00	0,00	0,02	0,02	0,00	0,00	0,00	0,12	0,00	0,00	0,00	0,00	0,00	0,00	0,02	0,00	0,00	0,00	0,00	0,04	0,00	0,00	0,00	0,00	0,02	0,00	0,00	0,10	0,04	0,17
0,00	0,00	0,08	0,00	0,00	0,00	0,00	0,00	0,00	0,00	0,00	0,00	0,00	0,00	0,00	0,00	0,00	0,27	0,00	0,00	0,00	0,00	0,15	0,02	0,00	0,23	0,00	0,00	0,00	0,00
0,00	0,00	0,00	0,12	0,00	0,00	0,02	0,12	0,00	0,00	0,06	0,00	0,00	0,00	0,13	0,00	0,02	0,00	0,00	0,00	0,00	0,06	0,00	0,00	0,08	0,00	0,00	0,40	0,00	0,00
0,00	0,00	0,33	0,00	0,00	0,87	0,44	0,02	0,08	0,00	0,00	0,87	0,00	0,02	0,00	0,00	0,00	0,00	0,00	0,06	0,00	0,02	0,00	0,04	0,00	0,02	0,00	0,00	0,67	0,00
0,00	0,00	0,06	0,00	0,00	0,00	0,00	0,02	0,00	0,00	0,00	0,04	0,00	0,00	0,00	0,00	0,02	0,00	0,00	0,02	0,00	0,02	0,00	0,04	0,00	0,02	0,00	0,04	0,02	0,10
0,00	0,00	0,00	0,00	0,00	0,00	0,00	0,06	0,00	0,00	0,06	0,00	0,00	0,00	0,02	0,04	0,15	0,00	0,02	0,02	0,00	0,08	0,00	0,00	0,04	0,02	0,00	0,02	0,00	0,02
0,00	0,00	0,00	0,00	0,00	0,00	0,00	0,00	0,00	0,00	0,00	0,00	0,00	0,00	0,00	0,00	0,00	0,00	0,19	0,00	0,04	0,00	0,00	0,00	0,00	0,00	0,00	0,00	0,00	0,00
0,00	0,00	0,00	0,02	0,00	0,00	0,04	0,35	0,00	0,00	0,00	0,00	0,00	0,10	0,04	0,00	0,00	0,00	0,00	0,00	0,00	0,10	0,00	0,00	0,02	0,06	0,00	0,02	0,00	0,00
0,00	0,00	0,02	0,71	0,00	0,00	0,04	0,19	0,00	0,00	0,06	0,00	0,00	0,00	0,02	0,00	0,00	0,00	0,00	0,08	0,00	0,06	0,00	0,81	0,00	0,02	0,02	0,08	0,00	0,00
0,00	0,00	0,04	0,04	0,48	0,00	0,02	0,02	0,00	0,00	0,00	0,00	0,04	0,00	0,00	0,00	0,02	0,00	0,21	0,02	0,00	0,02	0,00	0,00	0,00	0,17	0,04	0,12	0,00	0,02
0,00	0,00	0,25	0,02	0,21	0,00	0,06	0,00	0,00	0,00	0,00	0,00	0,00	0,00	0,06	0,00	0,77	0,00	0,06	0,10	0,00	0,17	0,00	0,00	0,00	0,27	0,00	0,00	0,00	0,00
0,00	0,00	0,08	0,00	0,00	0,04	0,00	0,00	0,00	0,00	0,00	0,10	0,08	0,00	0,00	0,00	0,00	0,48	0,04	0,48	0,00	0,00	0,27	0,06	0,00	0,02	0,06	0,00	0,00	0,00
0,00	0,00	0,00	0,00	0,00	0,00	0,00	0,00	0,00	0,00	0,00	0,00	0,00	0,00	0,00	0,00	0,00	0,00	0,02	0,00	0,00	0,00	0,00	0,00	0,00	0,00	0,00	0,00	0,02	0,13
0,00	0,00	0,06	0,00	0,00	0,00	0,17	0,02	0,00	0,00	0,00	0,00	0,00	0,00	0,00	0,00	0,00	0,00	0,15	0,02	0,87	0,00	0,00	0,00	0,00	0,02	0,00	0,06	0,23	0,40
0,00	0,00	0,00	0,00	0,00	0,00	0,00	0,00	0,00	0,00	0,00	0,00	0,00	0,00	0,00	0,00	0,00	0,00	0,00	0,00	0,00	0,00	0,00	0,00	0,00	0,00	0,00	0,00	0,00	0,00
yes	yes	-	-	-	-	-	-	yes	-	-	-	-	-	-	yes	-	-	-	-	-	-	-	-	-	-	-	-	-	-
341	340	339	338	337	336	335	334	333	332	331	330	329	328	327	326	325	324	323	322	321	320	319	318	317	316	315	314	313	312
-	-	-	-	-	-	-	-	-	-	-	-	-	-	-	-	-	-	-	-	-	-	-	-	-	-	-	-	-	-
339	338	337	336	335	334	333	332	331	330	329	328	327	326	325	324	323	322	321	320	319	318	317	316	315	314	313	312	311	310
-	-	-	-	-	-	-	-	-	-	-	-	yes	-	-	yes	yes	-	-	-	-	-	-	-	-	-	-	-	-	-
-	-	-	-	-	-	-	-	-	-	-	-	-	-	-	-	-	-	-	-	-	-	-	-	-	-	-	-	-	-
-	-	-	-	-	-	-	-	-	-	-	-	-	-	-	-	-	-	-	-	-	-	-	-	-	-	-	-	-	-
-	-	-	-	-	-	-	-	-	-	-	-	-	-	-	-	-	-	-	-	-	-	-	-	-	-	-	-	-	-

384	383	382	381	380	379	378	377	376	375	374	373	372	371	370	369	368	367	366	365	364	363	362	361	360	359	358	357	356	355
0,02	0,00	0,00	0,00	0,00	0,00	0,02	0,00	0,00	0,00	0,02	0,10	0,88	0,10	0,02	0,17	0,62	0,02	0,88	0,00	1,00	0,00	0,42	0,00	0,00	0,00	1,00	0,00	0,00	0,00
0,00	0,00	0,00	0,00	0,00	0,00	0,00	0,00	0,00	0,00	0,00	0,00	0,02	0,02	0,00	0,02	0,00	0,04	0,00	0,00	0,00	0,00	0,00	0,00	0,00	0,00	0,00	0,00	0,00	0,00
0,00	0,00	0,00	0,00	0,13	0,63	0,00	0,15	0,00	0,00	0,12	0,06	0,00	0,00	0,02	0,00	0,00	0,00	0,00	0,00	0,00	0,00	0,00	0,00	0,00	0,00	0,00	0,00	0,00	0,00
0,00	0,00	0,00	0,00	0,17	0,10	0,04	0,12	0,10	0,04	0,19	0,13	0,00	0,00	0,13	0,08	0,00	0,04	0,00	0,00	0,00	0,00	0,00	0,00	1,00	0,00	0,00	0,00	0,00	0,00
0,00	0,00	0,00	0,04	0,00	0,00	0,00	0,00	0,06	0,00	0,00	0,00	0,00	0,00	0,00	0,02	0,00	0,02	0,00	0,00	0,00	0,00	0,00	0,00	0,00	0,00	0,00	0,00	0,00	0,00
0,00	0,00	0,00	0,00	0,08	0,23	0,00	0,19	0,10	0,00	0,02	0,04	0,00	0,00	0,00	0,08	0,02	0,00	0,00	0,00	0,00	0,00	0,00	0,00	0,00	0,00	0,00	0,00	0,00	0,00
0,00	0,00	0,00	0,00	0,02	0,00	0,00	0,00	0,00	0,00	0,00	0,00	0,00	0,00	0,00	0,00	0,00	0,13	0,02	0,00	0,00	1,00	0,00	0,00	0,00	0,00	0,00	0,00	0,00	0,00
0,35	0,31	0,44	0,40	0,00	0,00	0,00	0,00	0,00	0,00	0,00	0,00	0,00	0,13	0,00	0,06	0,00	0,00	0,00	0,35	0,00	0,00	0,00	0,00	0,00	0,12	0,00	0,00	0,67	0,96
0,00	0,00	0,00	0,00	0,33	0,00	0,54	0,06	0,00	0,00	0,12	0,12	0,00	0,00	0,63	0,06	0,00	0,00	0,00	0,00	0,00	0,00	0,00	0,00	0,00	0,00	0,00	0,00	0,00	0,00
0,06	0,04	0,15	0,12	0,00	0,00	0,10	0,00	0,35	0,00	0,00	0,00	0,00	0,40	0,00	0,10	0,12	0,04	0,00	0,27	0,00	0,00	0,00	0,00	0,00	0,88	0,00	0,00	0,10	0,00
0,06	0,00	0,12	0,00	0,00	0,00	0,00	0,00	0,35	0,00	0,02	0,00	0,00	0,04	0,00	0,23	0,00	0,00	0,00	0,02	0,00	0,00	0,00	0,00	0,00	0,00	0,00	0,00	0,17	0,00
0,00	0,00	0,00	0,04	0,02	0,02	0,00	0,12	0,00	0,00	0,00	0,00	0,00	0,06	0,00	0,00	0,00	0,00	0,00	0,00	0,00	0,00	0,00	0,00	0,00	0,00	0,00	0,00	0,00	0,00
0,00	0,00	0,00	0,00	0,00	0,00	0,19	0,15	0,00	0,06	0,00	0,33	0,00	0,00	0,00	0,02	0,00	0,00	0,00	0,00	0,00	0,00	0,00	0,06	0,00	0,00	0,00	1,00	0,00	0,00
0,00	0,00	0,00	0,00	0,23	0,00	0,00	0,00	0,00	0,00	0,15	0,04	0,00	0,00	0,06	0,00	0,00	0,04	0,00	0,00	0,00	0,00	0,00	0,00	0,00	0,00	0,00	0,00	0,00	0,00
0,00	0,00	0,00	0,00	0,00	0,00	0,12	0,06	0,04	0,00	0,04	0,15	0,04	0,02	0,13	0,04	0,00	0,08	0,00	0,00	0,00	0,00	0,00	0,00	0,00	0,00	0,00	0,00	0,00	0,00
0,00	0,00	0,00	0,06	0,00	0,02	0,00	0,13	0,00	0,00	0,13	0,04	0,02	0,02	0,00	0,00	0,00	0,00	0,06	0,00	0,00	0,00	0,58	0,88	0,00	0,00	0,00	0,00	0,00	0,00
0,00	0,00	0,00	0,12	0,02	0,00	0,00	0,02	0,00	0,00	0,19	0,00	0,02	0,04	0,00	0,00	0,00	0,00	0,00	0,00	0,00	0,00	0,00	0,06	0,00	0,00	0,00	0,00	0,00	0,00
0,52	0,65	0,29	0,23	0,00	0,00	0,00	0,00	0,02	0,00	0,00	0,00	0,02	0,08	0,00	0,13	0,25	0,00	0,00	0,37	0,00	0,00	0,00	0,00	0,00	0,00	0,00	0,00	0,06	0,04
0,00	0,00	0,00	0,00	0,00	0,00	0,00	0,00	0,00	0,00	0,00	0,00	0,00	0,00	0,00	0,00	0,00	0,02	0,00	0,00	0,00	0,00	0,00	0,00	0,00	0,00	0,00	0,00	0,00	0,00
0,00	0,00	0,00	0,00	0,00	0,00	0,00	0,00	0,00	0,00	0,00	0,00	0,00	0,10	0,00	0,00	0,00	0,58	0,04	0,00	0,00	0,00	0,00	0,00	0,00	0,00	0,00	0,00	0,00	0,00
0,00	0,00	0,00	0,00	0,00	0,00	0,00	0,00	0,00	0,90	0,00	0,00	0,00	0,00	0,00	0,00	0,00	0,00	0,00	0,00	0,00	0,00	0,00	0,00	0,00	0,00	0,00	0,00	0,00	0,00
-	-	-	-	-	-	-	-	-	-	-	-	-	-	-	-	-	-	-	-	yes	yes	-	-	yes	-	yes	yes	-	yes
370	369	368	367	366	365	364	363	362	-	361	360	359	358	357	356	355	354	353	352	351	350	349	348	347	346	345	344	343	342
-	-	-	-	-	-	-	-	-	-	-	-	-	-	-	-	-	-	-	-	-	-	-	-	yes	yes	yes	-	-	-
368	367	366	365	364	363	362	361	360	-	359	358	357	356	355	354	353	352	351	350	349	348	347	346	345	344	343	342	341	340
-	-	-	-	-	-	-	-	-	-	-	-	-	-	-	-	-	-	-	-	-	-	-	-	yes	-	-	-	-	-
-	-	-	-	-	-	-	-	-	-	-	-	-	-	-	-	-	-	-	-	-	-	-	-	yes	-	-	-	-	-
-	-	-	-	-	-	-	-	-	-	-	-	-	-	-	-	-	-	-	-	-	-	-	-	-	-	-	-	-	-
-	-	-	-	-	-	-	-	-	-	-	-	-	-	-	-	-	-	-	-	-	-	-	-	-	-	-	-	-	-

414	413	412	411	410	409	408	407	406	405	404	403	402	401	400	399	398	397	396	395	394	393	392	391	390	389	388	387	386	385
0,00	0,00	0,02	0,04	0,00	0,06	0,02	0,02	0,04	0,02	0,08	0,08	0,02	0,02	0,17	0,02	0,63	0,08	0,04	0,17	0,00	0,00	0,00	0,00	0,00	0,00	0,00	0,00	0,00	0,00
0,00	0,00	0,00	0,00	0,00	0,02	0,00	0,00	0,00	0,00	0,00	0,02	0,00	0,00	0,00	0,00	0,00	0,00	0,00	0,00	0,00	0,00	0,00	0,00	0,00	0,00	0,00	0,06	0,00	0,06
0,00	0,00	0,00	0,00	0,00	0,00	0,00	0,04	0,08	0,00	0,17	0,02	0,04	0,10	0,00	0,00	0,00	0,00	0,02	0,04	0,00	**1,00**	0,00	**1,00**	0,00	0,00	0,00	0,00	0,00	0,00
0,00	0,00	0,00	0,02	0,02	0,00	0,08	0,04	0,00	0,06	0,17	0,04	0,12	0,02	0,08	0,10	0,00	0,00	0,19	0,12	0,00	0,00	0,00	0,00	0,00	0,00	0,00	0,00	0,00	0,00
0,00	0,00	0,00	0,00	0,00	0,00	0,02	0,00	0,02	0,02	0,00	0,00	0,00	0,02	0,02	0,00	0,02	0,00	0,00	0,15	0,00	0,00	0,00	0,00	0,00	0,00	0,00	0,00	0,00	0,00
0,00	0,00	0,00	0,02	0,00	0,00	0,02	0,08	0,06	0,02	0,02	0,13	0,71	0,02	0,00	0,08	0,00	0,00	0,02	0,00	0,00	0,00	0,00	0,00	**1,00**	0,00	**1,00**	0,00	0,00	0,00
0,00	0,00	0,00	0,00	0,02	0,00	0,00	0,00	0,04	0,00	0,00	0,00	0,00	0,00	0,04	0,00	0,00	0,00	0,00	0,06	0,00	0,00	0,00	0,00	0,00	0,00	0,00	0,00	0,00	0,00
0,00	0,02	0,00	0,00	0,00	0,00	0,00	0,00	0,00	0,19	0,06	0,12	0,00	0,00	0,08	0,00	0,02	0,12	0,08	0,00	0,17	0,00	0,00	0,00	0,00	0,00	0,00	0,00	0,00	0,00
0,00	0,00	0,00	0,00	0,04	0,02	0,00	0,04	0,02	0,04	0,08	0,04	0,00	0,10	0,00	0,23	0,02	0,00	0,00	0,00	0,00	0,00	**1,00**	0,00	0,00	0,00	0,00	0,00	0,00	0,00
0,00	0,00	0,00	0,00	0,02	0,02	0,00	0,02	0,02	0,31	0,00	0,04	0,00	0,46	0,37	0,00	0,06	0,02	0,02	0,02	0,12	0,00	0,00	0,00	0,00	0,00	0,00	0,00	0,94	0,00
0,00	0,00	0,00	0,00	0,00	0,00	0,00	0,00	0,00	0,00	0,00	0,02	0,00	0,13	0,06	0,00	0,15	0,02	0,02	0,00	0,19	0,00	0,00	0,00	0,00	0,00	0,00	0,00	0,02	0,00
0,00	0,00	0,00	0,00	0,00	0,00	0,04	0,00	0,02	0,00	0,08	0,00	0,00	0,00	0,00	0,02	0,00	0,00	0,00	0,13	0,00	0,00	0,00	0,00	0,00	0,00	0,00	0,00	0,00	0,88
0,00	0,00	0,00	0,00	0,00	0,00	0,00	0,02	0,00	0,02	0,00	0,00	0,00	0,00	0,00	0,02	0,00	0,00	0,00	0,02	0,00	0,00	0,00	0,00	0,00	0,00	0,00	0,00	0,00	0,00
0,04	0,00	0,00	0,00	0,00	0,00	0,04	0,04	0,00	0,02	0,04	0,02	0,02	0,02	0,02	0,04	0,04	0,00	0,12	0,08	0,00	0,00	0,00	0,00	0,00	0,00	0,00	0,00	0,00	0,00
0,00	0,00	0,00	0,02	0,02	0,00	0,02	0,00	0,06	0,02	0,06	0,10	0,06	0,06	0,00	0,35	0,02	0,00	0,00	0,00	0,00	0,00	0,00	0,00	0,00	**1,00**	0,00	0,00	0,00	0,00
0,00	0,00	0,00	0,00	0,00	0,02	0,02	0,00	0,00	0,02	0,06	0,00	0,00	0,04	0,00	0,15	0,02	0,00	0,06	0,02	0,00	0,00	0,00	0,00	0,00	0,00	0,00	0,94	0,00	0,04
0,00	0,00	0,00	0,00	0,00	0,00	0,00	0,00	0,00	0,02	0,10	0,00	0,00	0,00	0,00	0,00	0,00	0,00	0,44	0,06	0,00	0,00	0,00	0,00	0,00	0,00	0,00	0,00	0,00	0,02
0,00	0,00	0,00	0,00	0,00	0,02	0,00	0,00	0,00	0,12	0,06	0,35	0,00	0,02	0,06	0,00	0,02	0,77	0,00	0,10	0,52	0,00	0,00	0,00	0,00	0,00	0,00	0,00	0,04	0,00
0,00	0,00	0,00	0,00	0,00	0,00	0,00	0,00	0,00	0,00	0,00	0,00	0,02	0,00	0,00	0,00	0,00	0,00	0,00	0,00	0,00	0,00	0,00	0,00	0,00	0,00	0,00	0,00	0,00	0,00
0,00	0,00	0,00	0,00	0,00	0,00	0,00	0,00	0,00	0,00	0,00	0,00	0,00	0,00	0,12	0,00	0,00	0,00	0,00	0,04	0,00	0,00	0,00	0,00	0,00	0,00	0,00	0,00	0,00	0,00
**0,96**	**0,98**	**0,98**	0,90	0,88	0,85	0,75	0,71	0,65	0,13	0,04	0,04	0,02	0,00	0,00	0,00	0,00	0,00	0,00	0,00	0,00	0,00	0,00	0,00	0,00	0,00	0,00	0,00	0,00	0,00
-	-	-	-	-	-	-	-	-	-	-	-	-	-	-	-	-	-	-	-	-	yes	yes	yes	yes	yes	yes	yes	yes	-
401	400	399	398	397	396	395	394	393	392	391	390	389	387, 388	386	385	384	383	382	381	380	379	378	377	376	375	374	373	372	371
-	-	-	-	-	-	-	-	-	-	-	-	-	-	-	yes	-	-	yes	-	-	yes	-	-	yes	-	yes	yes	-	-
-	-	-	-	-	-	-	-	-	-	-	-	-	385	384	383	382	381	380	379	378	377	376	375	374	373	372	371	370	369
-	-	-	-	-	-	-	-	-	-	-	-	-	-	-	-	-	-	-	-	-	yes	-	-	yes	-	-	yes	-	-
-	-	-	-	-	-	-	-	-	-	-	-	-	-	-	-	-	-	-	-	-	yes	-	-	yes	-	-	yes	-	-
-	-	-	-	-	-	-	-	-	-	-	-	-	-	-	-	-	-	-	-	-	-	-	-	-	-	-	-	-	-
-	-	-	-	-	-	-	-	-	-	-	-	-	-	-	-	-	-	-	-	-	-	-	-	-	-	-	-	-	-

415	0,00	0,00	0,00	0,06	0,00	0,00	0,00	0,00	0,00	0,00	0,00	0,00	0,00	0,00	0,00	0,00	0,00	0,00	0,00	0,00	0,94	-	-	-	-	-	-	-	-
416	0,00	0,00	0,00	0,00	0,00	0,00	0,00	0,00	0,00	0,00	0,00	0,00	0,00	0,00	0,00	0,00	0,00	0,06	0,00	0,00	0,94	-	-	-	-	-	-	-	-
417	0,00	0,00	0,00	0,02	0,00	0,00	0,00	0,00	0,00	0,00	0,00	0,00	0,00	0,00	0,00	0,00	0,00	0,00	0,00	0,00	**0,98**	-	-	-	-	-	-	-	-
418	0,00	0,00	0,00	0,00	0,00	0,00	0,00	0,00	0,00	0,00	0,00	0,00	0,02	0,00	0,02	0,00	0,00	0,00	0,00	0,00	**0,96**	-	-	-	-	-	-	-	-
419	0,00	0,00	0,00	0,02	0,00	0,00	0,00	0,00	0,00	0,00	0,00	0,00	0,00	0,00	0,00	0,00	0,02	0,00	0,00	0,00	**0,96**	-	-	-	-	-	-	-	-
420	0,02	0,00	0,00	0,00	0,00	0,00	0,00	0,00	0,00	0,00	0,00	0,00	0,00	0,00	0,02	0,00	0,00	0,00	0,00	0,00	**0,96**	-	-	-	-	-	-	-	-
421	0,00	0,00	0,00	0,00	0,00	0,02	0,00	0,00	0,00	0,00	0,00	0,00	0,00	0,00	0,02	0,00	0,00	0,00	0,00	0,00	**0,96**	-	-	-	-	-	-	-	-
422	0,00	0,00	0,00	0,00	0,00	0,02	0,00	0,00	0,02	0,04	0,00	0,00	0,00	0,00	0,00	0,00	0,02	0,00	0,00	0,00	0,90	-	-	-	-	-	-	-	-
423	0,06	0,00	0,00	0,00	0,00	0,00	0,00	0,00	0,02	0,02	0,00	0,00	0,00	0,00	0,00	0,00	0,00	0,00	0,00	0,00	0,90	-	-	-	-	-	-	-	-

The conservation level of TrpB residues was calculated using an MSA of 52 sequences from extant TrpB variants from our previous study[10].

Supplementary Schemes:

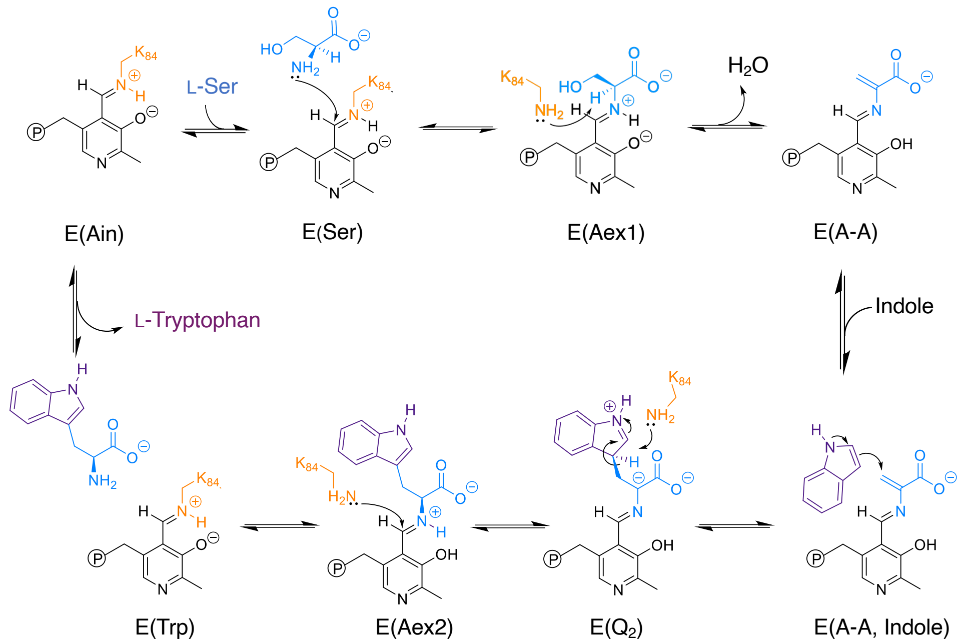

Scheme S1. Tryptophan synthase reaction mechanism of the TrpB subunit. The catalytic K86 residue is colored in orange, while the L-Ser and indole substrates are blue and purple, respectively. In the resting state, a pyridoxal phosphate (PLP)-cofactor is covalently linked to the K84 active site residue, forming a Schiff base intermediate (E(Ain)). After transamination with L-serine E(Ser), an external aldimine intermediate E(Aex1) is formed. This intermediate undergoes deprotonation at C_α, aided by K84, which is followed by a rapid elimination of the Aex1 hydroxyl group to form an electrophilic amino acrylate intermediate E(A−A). At this point, indole formed in TrpA reaches the TrpB active site and reacts with E(A−A) to form a quinonoid intermediate E(Q_2), which after proton extraction generates E(Q3) (not shown). The reaction mechanism follows with the protonation at C_α of Q_3 by K84 to form the E(Aex2) intermediate, which undergoes a second transamination reaction to finally release the L-tryptophan E(Trp) product and recover the resting state of the enzyme.

Supplementary Figures:

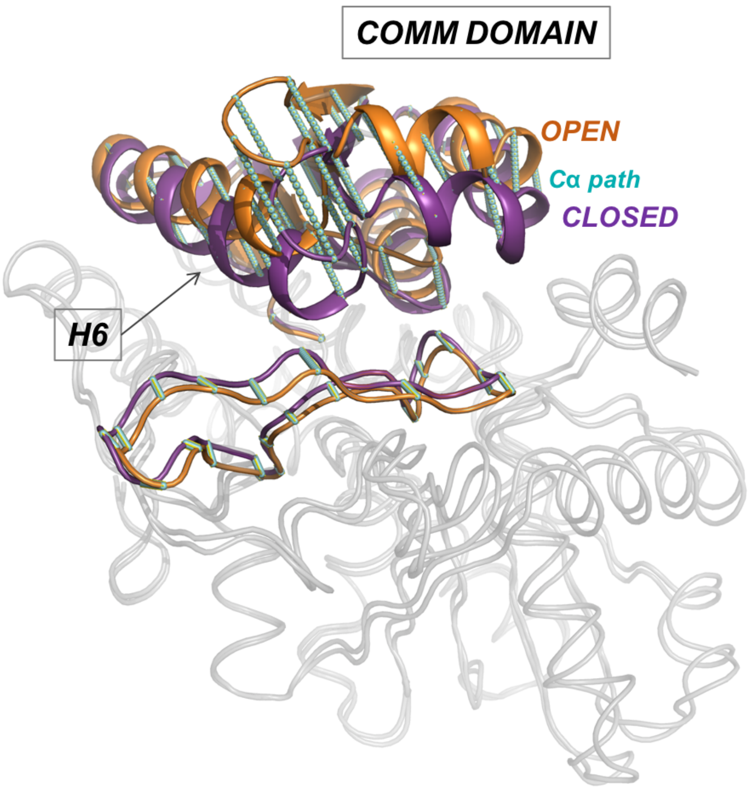

Figure S1. Representation of the Open-to-Closed path of collective variables. The α carbon atoms encompassing the path (shown as cyan spheres) corresponds to the COMM domain residues (97-184) and the loop residues (282-305) going from the Open (PDB ID: 1WDW, in orange) to the Closed (PDB ID: 3CEP, in purple) X-ray structures.

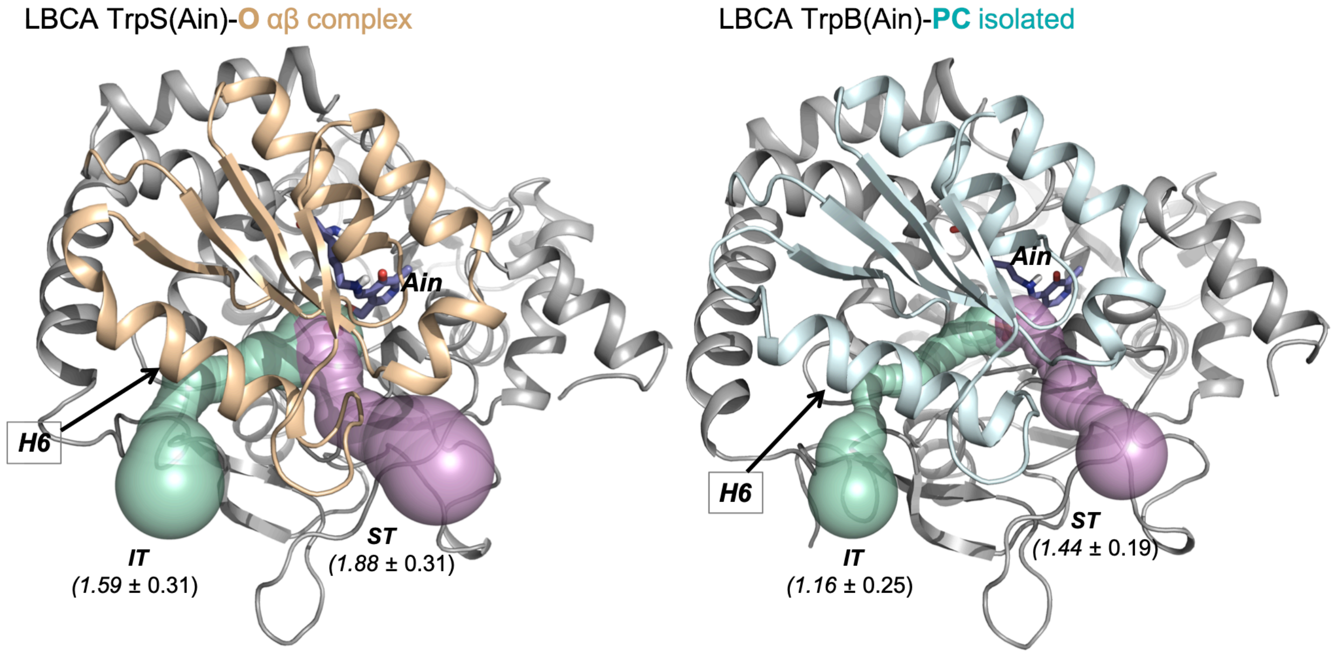

Figure S2. Representation of the tunnel access at Ain reaction intermediate for the L-Ser substrate. The substrate access tunnels that were computed with CAVER 3.0 of the O and PC conformational ensembles corresponding to the LBCA TrpS complex and LBCA TrpB isolated respectively, revealed two different entry paths: the previously reported TrpA-TrpB internal tunnel (IT, green) and a secondary tunnel (ST, violet) that our group recently characterized.[2] The averaged bottleneck radii values (in Å) are also shown. We previously hypothesized that the secondary tunnel may play a role in the L-Ser entry but also in the L-Trp release.[2] The larger bottleneck radius of the ST in comparison with the IT highlights that the ST must be preferred for the L-Ser entry in both systems. The LBCA TrpS(Ain)-O state exhibited a larger bottleneck radius than the LBCA TrpB(Ain)-PC state in both tunnels, which indicates that the open state stabilization driven by the TrpA allosteric communication improves substrate accessibility.

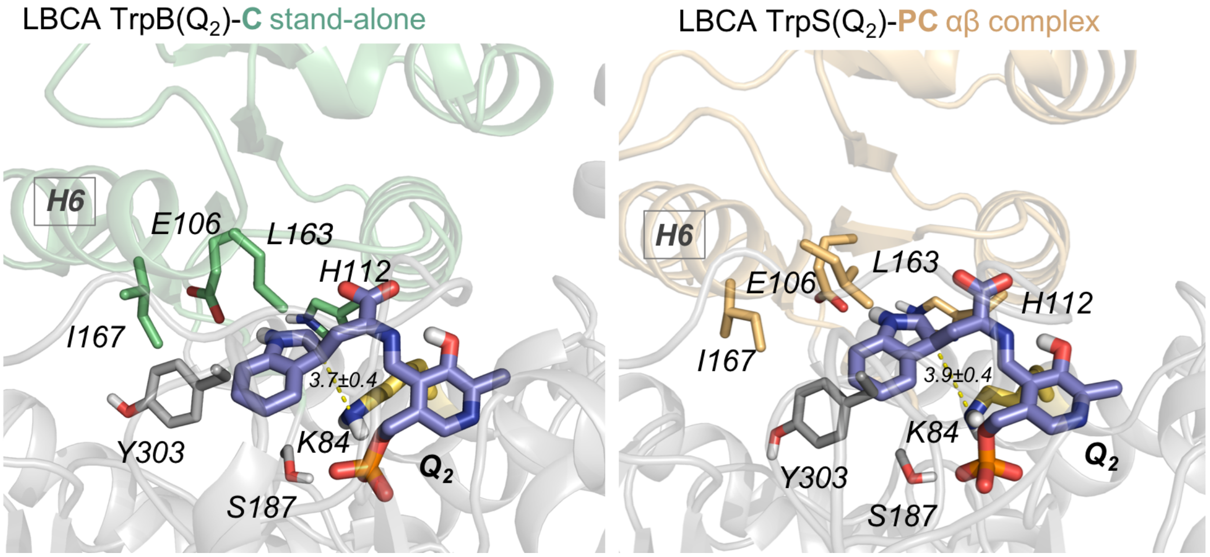

Figure S3. Detailed active site view. Shown are the metastable conformations of the C state of LBCA TrpB (left) and PC state of LBCA TrpS (right) at the Q_2 reaction intermediate (violet) present in both structures. Active site residues are depicted in stick representation, the COMM domain is colored green (LBCA TrpB) or orange (LBCA TrpS), and catalytic K84 is highlighted in yellow. The number next to the yellow dashed line indicates the catalytic proton transfer distance (in Å) between K84 and the reaction intermediate

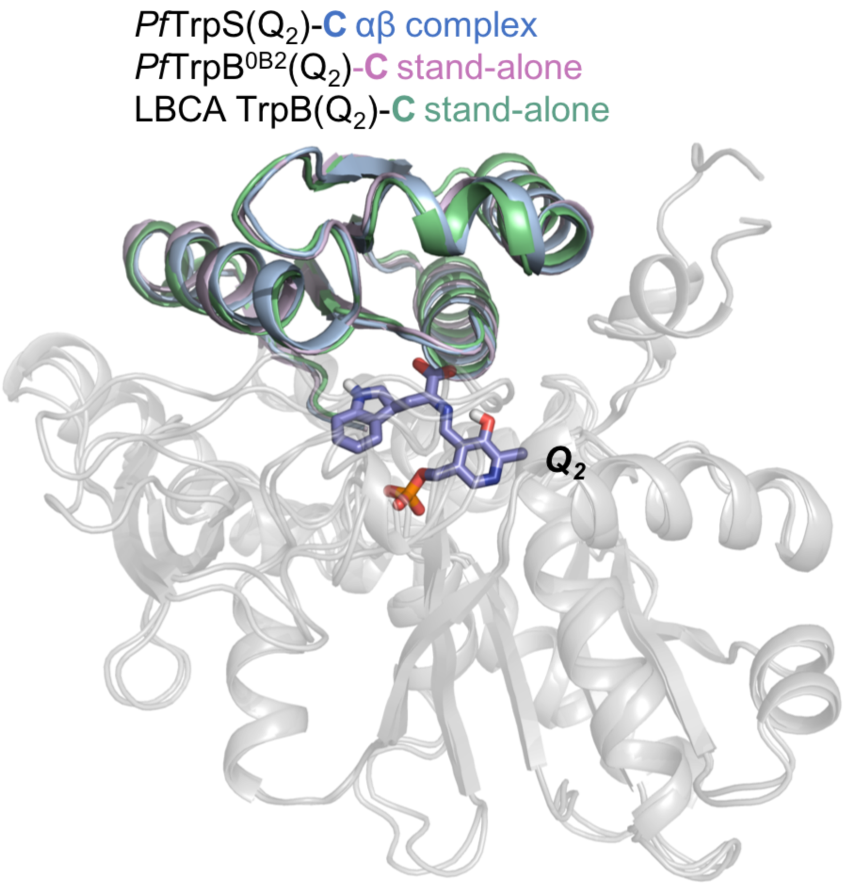

Figure S4. Overlays of the metastable conformations of the closed (C) states at Q_2 intermediate. The COMM domain is highlighted for *pf*TrpS (blue), the *pf*TrpB^{0B2} (pink) and LBCA TrpB (green). The *pf*TrpS and *pf*TrpB^{0B2} metastable structures were obtained from reference [2].

The FEL of the LBCA TrpB(Q_2) shows an energy minimum in the region that corresponds to a closed COMM conformations with large and low MSD distances equal in energy (Fig. **2a**). In this context, we previously identified an energy minimum corresponding to low deviated closed conformations for the allosterically regulated *pf*TrpS complex and also for the stand-alone evolved variant *pf*TrpB^{0B2}.[2] These closed conformational ensembles displayed efficient active site preorganization by means of optimized non-covalent interactions networks and short catalytic distance between the Q_2 intermediate and the catalytic K84 that acts as proton acceptor. In particular the H6 was found to play an important role in the closure of the COMM domain and to form non-covalent interactions with the indole moiety of Q_2. Structural comparison between the metastable structure from the *pf*TrS(Q_2)-C state, the *pf*TrB^{0b2}(Q_2)-C and the LBCA TrpB(Q_2)-C from the low deviated closed ensemble obtained here showed that LBCA TrpB displays a highly similar degree of closure and also similar K84-Q_2 proton transfer distances. This indicates that LBCA TrpB has stand-alone properties due to the fact that it explores a stable catalytically competent closed conformation in the absence of TrpA.

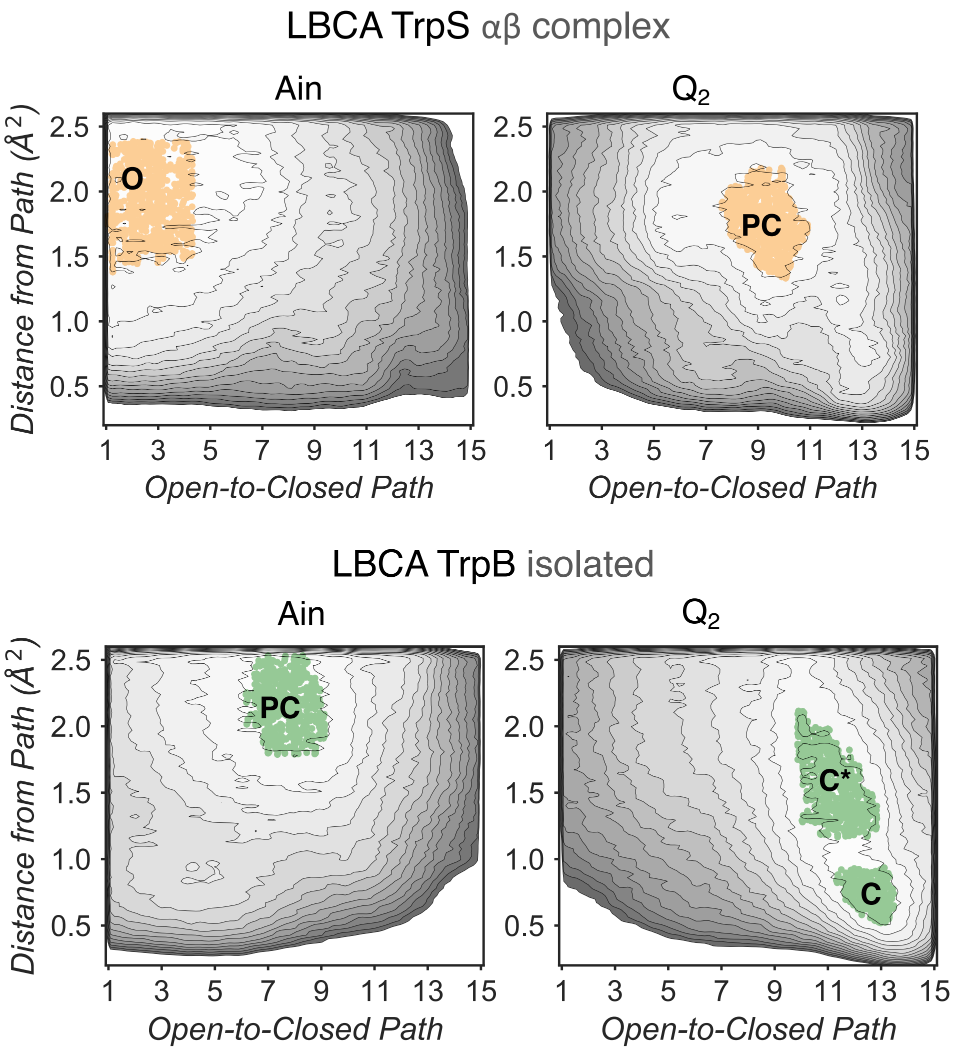

Figure S5. Projection of the conformations that correspond to the local energy minima coordinates from the metadynamics simulations on the FEL. The conformations projected are depicted as orange (LBCA TrpS complex) and green (LBCA TrpB isolated) dots. The representative metastable conformations of each local energy minimum presented in the main text were obtained clustering these set of structures.

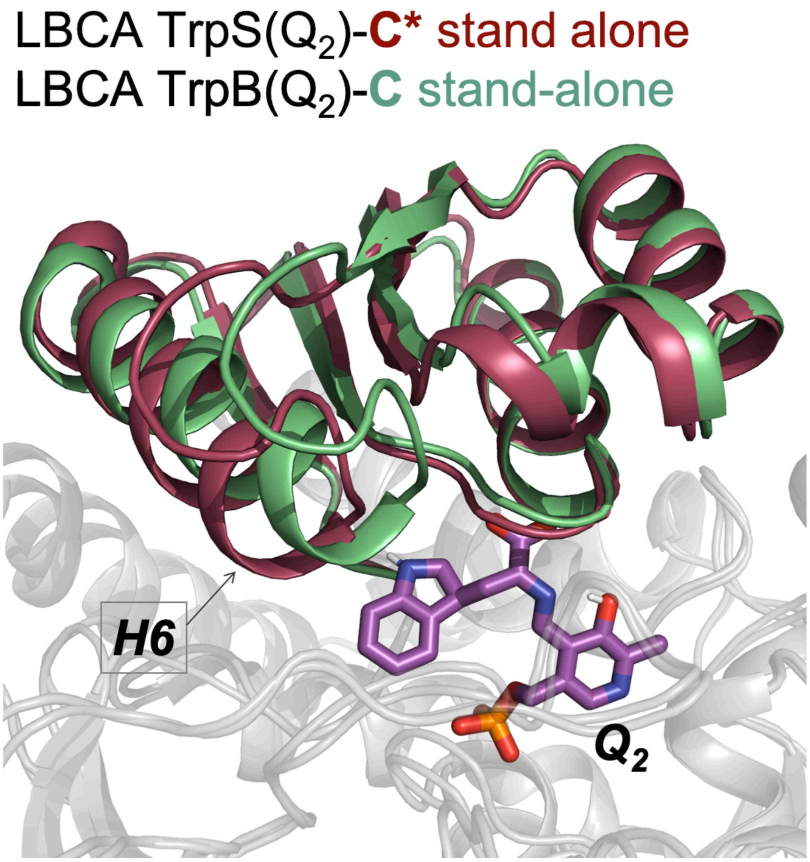

Figure S6. Overlays of the metastable conformations of the deviated closed (C*) state (garnet) and the productive (C) state (green) of LBCA TrpB at Q_2 intermediate.

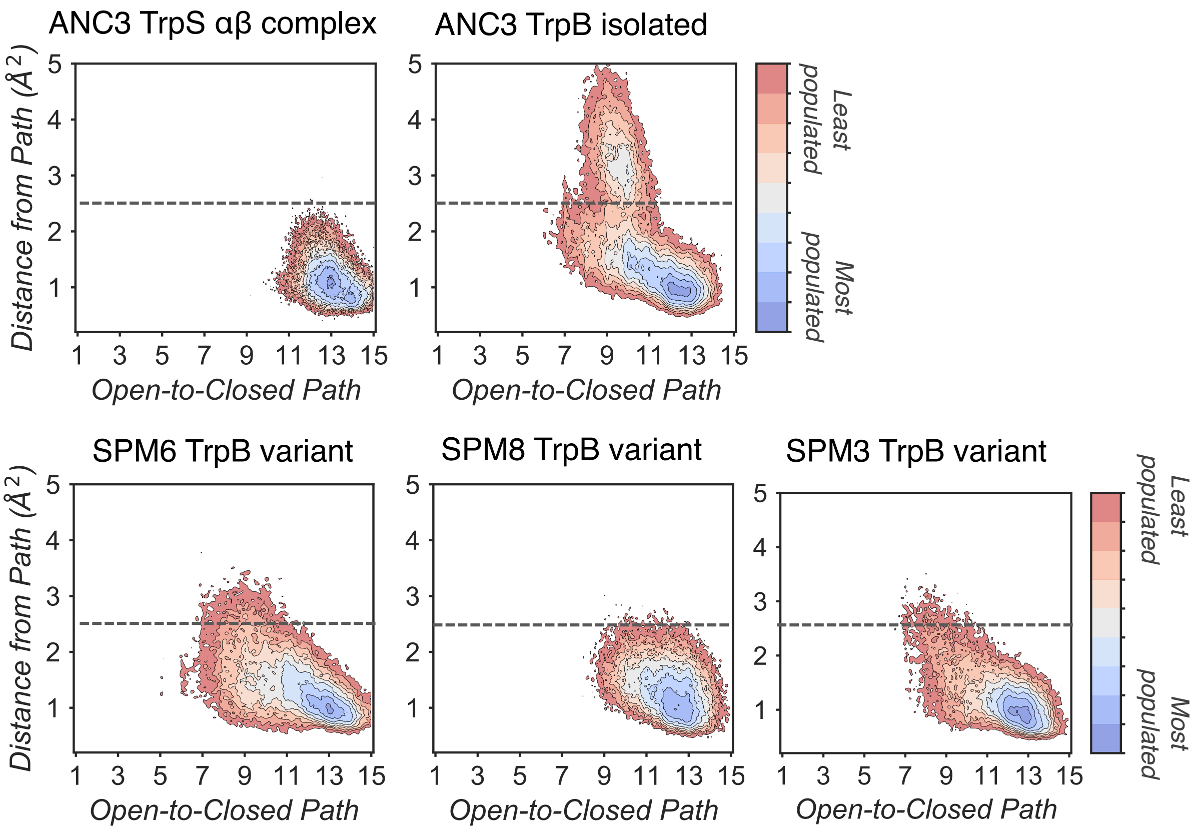

Figure S7. Population analysis of the conventional molecular dynamics simulations. The MD data is plotted as a function of the same collective variables used for the metadynamics simulations (i.e. Open-to-Closed conformational transition from the X-ray data (reference path) and the MSD distance from the reference path). The dashed gray line indicates the y-axis top deviation value monitored in the metadynamics calculations (i.e. 2.5 $Å^2$). Note that the inefficient ANC3 TrpB isolated samples non-productive COMM domain conformations (above the dashed line, high MSD distances), while the ANC3 TrpS in complex and the designed SPM enzyme variants better retain catalytically productive closed conformations (below the dashed line, low MSD distances). All the systems were simulated at the Q_2 intermediate.

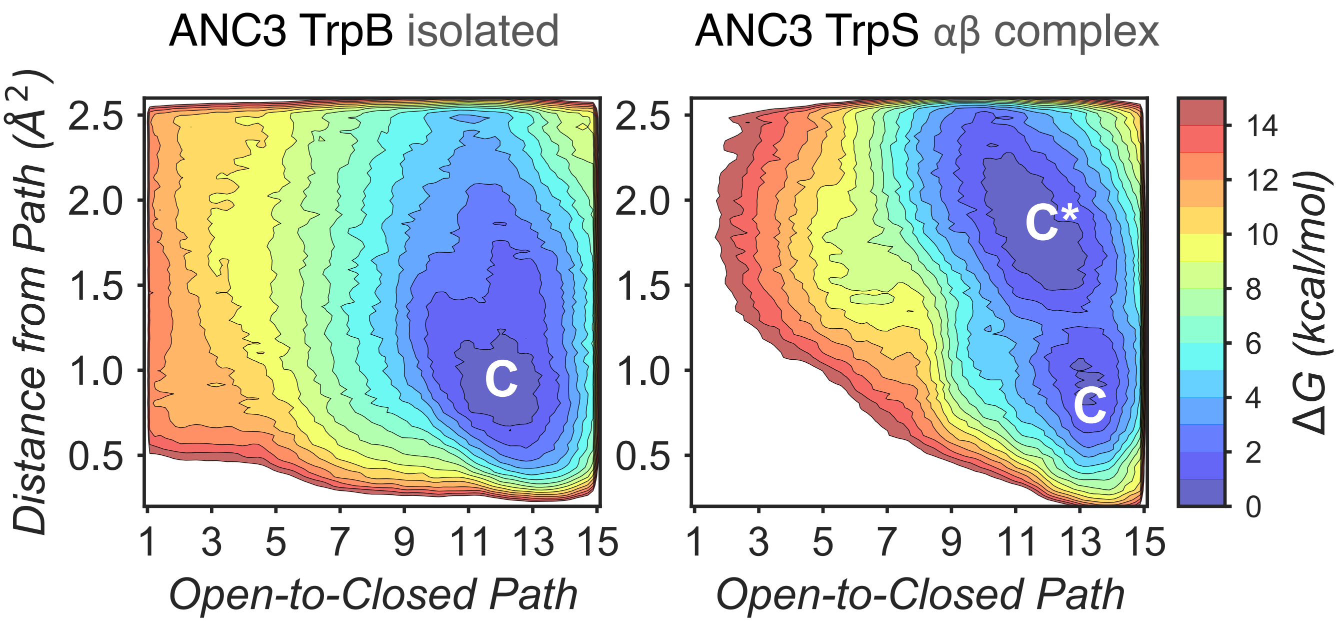

Figure S8. Computational exploration of the ANC3 conformational ensemble. Free energy landscape (FEL) associated with the COMM domain open-to-closed (O-to-C) conformational transition of ANC3 TrpB and ANC3 TrpS at Q_2 reaction intermediate. The x-axis corresponds to the progression along the reference O-to-C path generated from X-Ray data, while the y-axis corresponds to the mean square deviation (MSD) distance from the reference path.

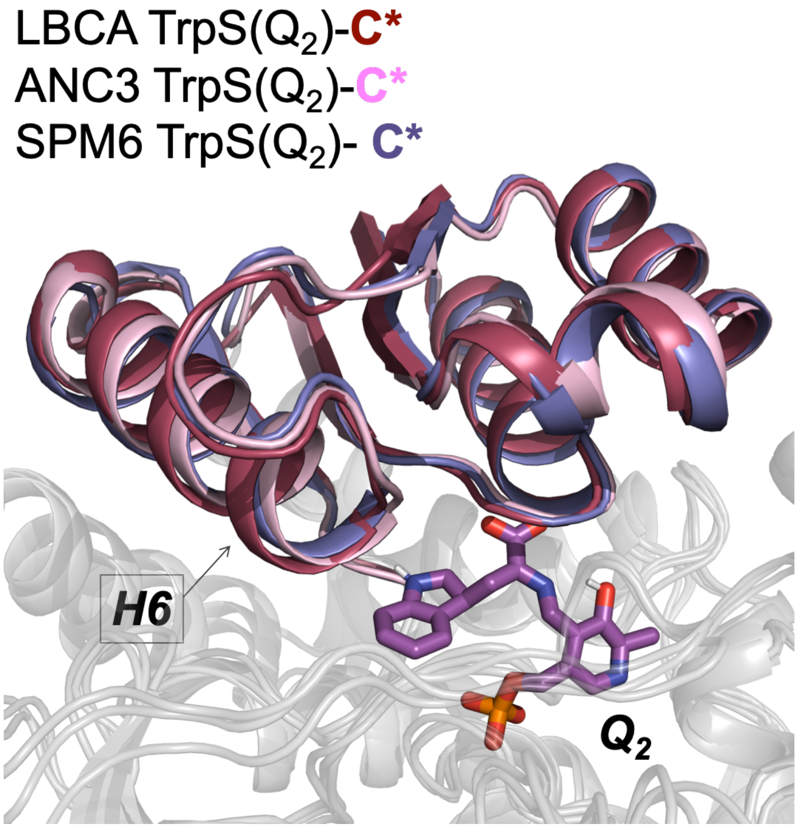

Figure S9. Overlays of the metastable conformations of the deviated closed (C*) state for the LBCA TrpS (garnet), ANC3 TrpS (pink) and SPM6 TrpS (purple) at Q_2 intermediate.

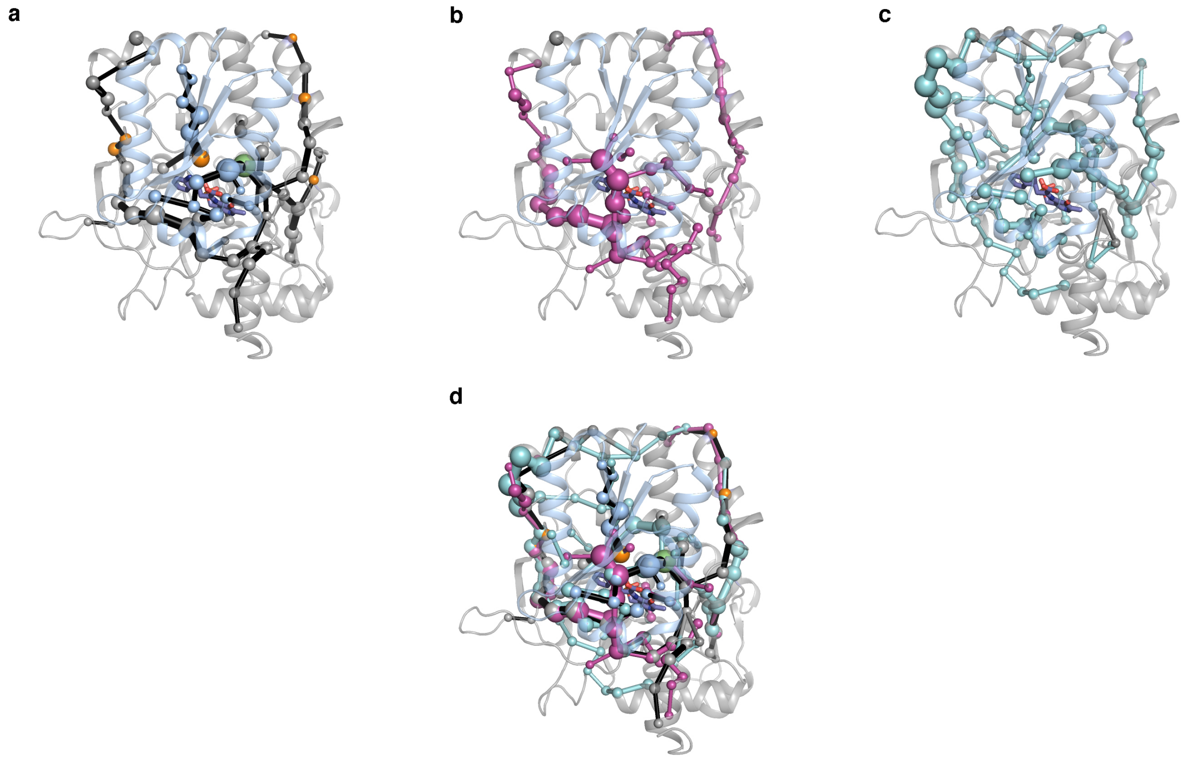

Figure S10. Computed SPM for the different TrpB systems: (a) LBCA TrpB (shown in gray/blue/orange spheres and black edges), (b) ANC3 TrpB (in pink), (c) *pf*TrpB in complex (in cyan). An overlay of all computed SPM is shown in panel (d).

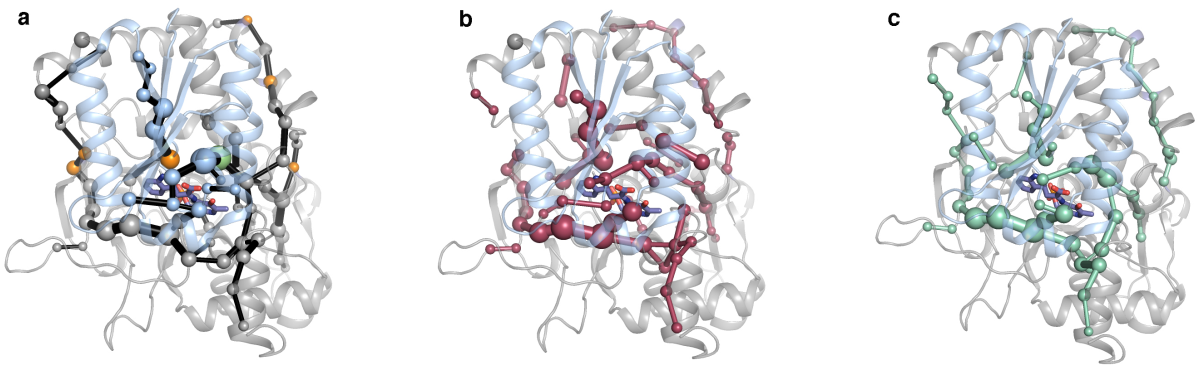

Figure S11. Calculation of SPM of LBCA using different thresholds: (a) default distance of 6 Å, (b) 6.5 Å and (c) 7 Å. Although some additional positions are identified with the longer distance thresholds, the generated SPMs are rather similar and indeed contain the 6 hotspots targeted for SPM6 variant generation (marked in orange).

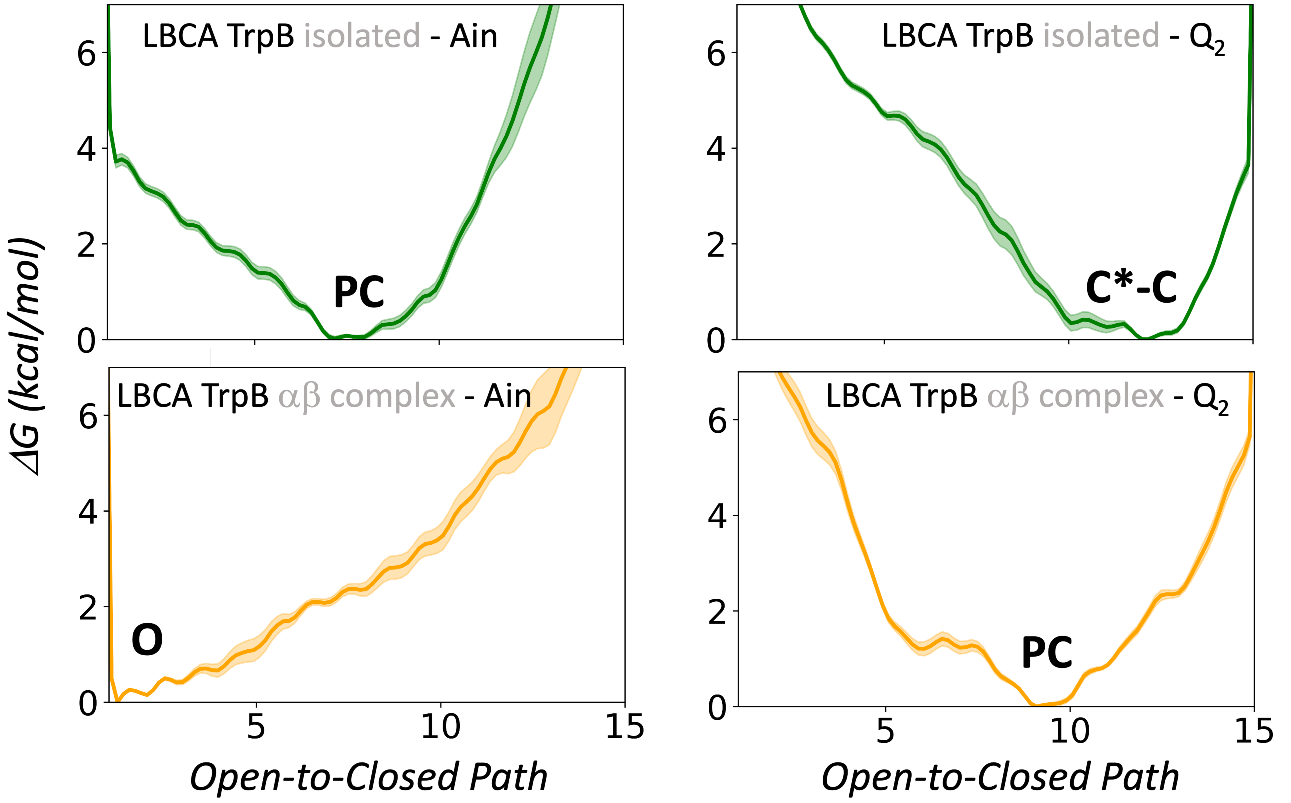

Figure S12. 2D Free energy landscapes (FELs) associated with the COMM domain open-to-closed (O-to-C) conformational transition with associated errors, for LBCA TrpB and LBCA TrpS at the Ain and Q_2 reaction intermediates. The x-axis corresponds to the progression along the reference O-to-C path generated from X-Ray data, while the y-axis corresponds to free energy differences associated with conformational progression. Average free energies are shown with a solid line, whereas shaded areas correspond to the associated errors.

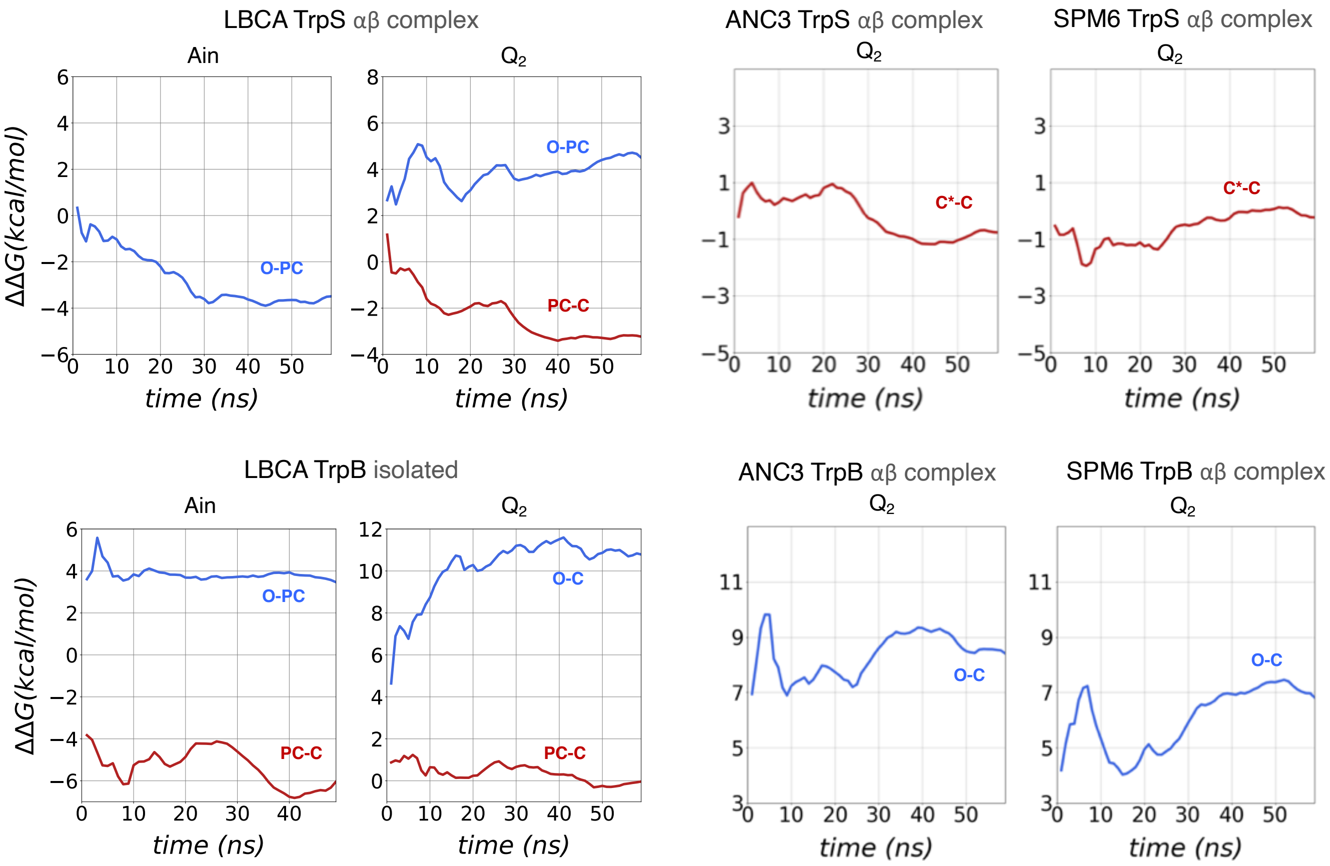

Figure S13 Estimate of the differences in energy between selected regions of the FEL surface along the metadynamics simulations**.** The lines represent the mean ΔΔG value of the 10 walker replicas along the simulation time for LBCA TrpS and LBCA TrpB at Ain and Q_2 intermediates together with ANC3 TrpS, ANC3 TrpB, SPM6 TrpS and SPM6 TrpB at Q_2 intermediate. The line labels indicate the regions of the energy landscape that have been computed. With increasing simulation time, all lines tend to flatten, which is indicative of FEL convergence.

References:

1. Case, D. A.; Darden, T. A.; Cheatham, T. E.; Simmerling, C. L.; Wang, J.; Duke, R. E.; Luo, R.; Crowley, M.; Walker, R. C.; Zhang, W.; Merz, K. M.; Wang, B.; Hayik, S.; Roitberg, A.; Seabra, G.; Kolossváry, I.; Wong, K. F.; Paesani, F.; Vanicek, J.; Wu, X.; Brozell, S. R.; Steinbrecher, T.; Gohlke, H.; Yang, L.; Tan, C.; Mongan, J.; Hornak, V.; Cui, G.; Mathews, D. H.; Seetin, M. G.; Sagui, C.; Babin, V.; Kollman, P. A., *AMBER 16, University of California, San Francisco, 2016.*
2. Maria-Solano, M. A.; Iglesias-Fernández, J.; Osuna, S., Deciphering the Allosterically Driven Conformational Ensemble in Tryptophan Synthase Evolution. *J. Am. Chem. Soc.* **2019,** *141* (33), 13049-13056.
3. Barducci, A.; Bonomi, M.; Parrinello, M., Metadynamics. *Wiley Interdiscip. Rev.-Comput. Mol. Sci.* **2011,** *1* (5), 826-843.
4. Tribello, G. A.; Bonomi, M.; Branduardi, D.; Camilloni, C.; Bussi, G., PLUMED 2: New feathers for an old bird. *Comput. Phys. Commun* **2014,** *185* (2), 604-613.
5. Pronk, S.; Pall, S.; Schulz, R.; Larsson, P.; Bjelkmar, P.; Apostolov, R.; Shirts, M. R.; Smith, J. C.; Kasson, P. M.; van der Spoel, D.; Hess, B.; Lindahl, E., GROMACS 4.5: a high-throughput and highly parallel open source molecular simulation toolkit. *Bioinformatics* **2013,** *29* (7), 845-54.
6. Chovancova, E.; Pavelka, A.; Benes, P.; Strnad, O.; Brezovsky, J.; Kozlikova, B.; Gora, A.; Sustr, V.; Klvana, M.; Medek, P.; Biedermannova, L.; Sochor, J.; Damborsky, J., CAVER 3.0: a tool for the analysis of transport pathways in dynamic protein structures. *PLoS Comput. Biol.* **2012,** *8* (10), e1002708.
7. Romero-Rivera, A.; Garcia-Borràs, M.; Osuna, S., Role of Conformational Dynamics in the Evolution of Retro-Aldolase Activity. *ACS Catal.* **2017,** *7* (12), 8524-8532.
8. Osuna, S., The challenge of predicting distal active site mutations in computational enzyme design. *Wiley Interdiscip. Rev. Comput. Mol. Sci.* **2020**, e1502.
9. Rohweder, B.; Semmelmann, F.; Endres, C.; Sterner, R., Standardized cloning vectors for protein production and generation of large gene libraries in Escherichia coli. *BioTechniques* **2018,** *64* (1), 24-26.
10. Schupfner, M.; Straub, K.; Busch, F.; Merkl, R.; Sterner, R., Analysis of allosteric communication in a multienzyme complex by ancestral sequence reconstruction. *Proc. Natl. Acad. Sci. U. S. A.* **2020,** *117* (1), 346-354.
